# Supplementary material for: Characterization of Plant-Derived Natural Inhibitors of Dipeptidyl Peptidase-4 as Potential Antidiabetic Agents: A Computational Study
Source: Pharmaceutics. 2024 Apr 1;16(4):483. doi: 10.3390/pharmaceutics16040483 (PMC11053753; doi:10.3390/pharmaceutics16040483)
Supplement: Supplementary file 1 [file pharmaceutics-16-00483-s001.zip › pharmaceutics-2889854-supplementary.pdf]

Supplementary figure

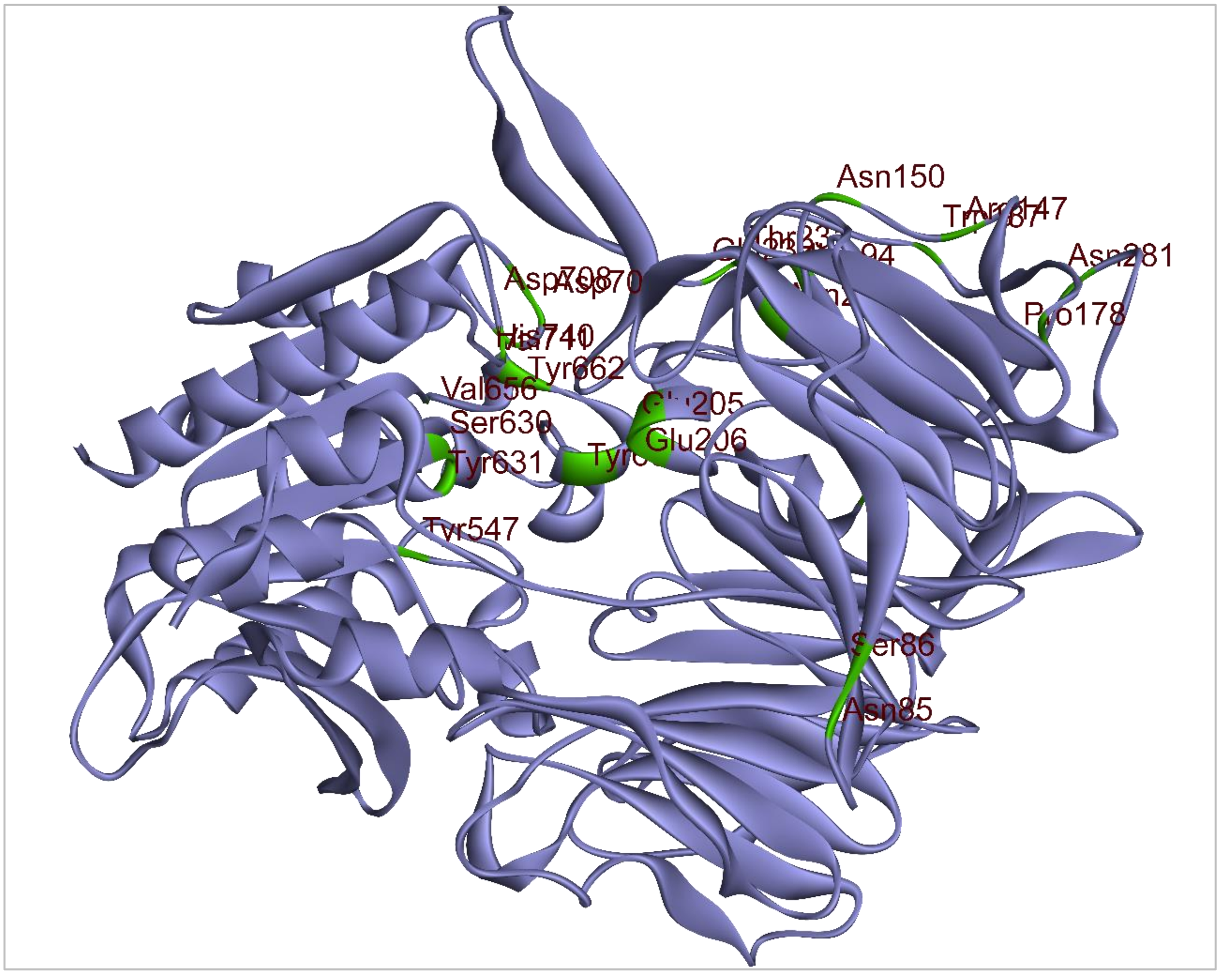

Supplementary Figure S1. Figure shows that the DPP-4 protein has several crucial regions responsible for the catalytic and binding sites and their pockets S1 and S2, including residues.

Supplementary Table S1. Library of a complete list of phytochemicals with their CID obtained from 81 medicinal plants in this study

Plant Name	Phytochemical Name	PubChem CID	References
1. **Atractylodes lancea**	α-Guaiene	348291106	[1]
	Modephene	11030947	
	Berkheyaradulene	14034017	
	Caryophyllene	5354499	
	γ-Elemene	472424234	
	Elemol	92138	
	Atractylon	3080635	
	Hinesol	10878761	
	β-Eudesmol	482040418	
	1-Hexadecanol	2682	
	Atractylodin	5321047	
	α-Pinene	258567152	[2]
	α-Phellandrene	198941074	
	α-Elemene	198974946	
	α-Himachalene	319347531	
	α-Humulene	481115162	
	Undecane	14257	
	Cyclohexane, 1-methyl-3-pentyl-	566169	
	β-Cedrene	102432	
	Cadinene	3032853	
	Isocomene	188113	
	ɣ-Gurjunene	90805	
	Calamene	11298625	
	2,2-Dimethyl-1-(2,4,6-trimethylphenyl)propan-1-one	590157	
	Eudesma-4(14),7(11)-diene	6432497	
	Eudesma-4(14),11-diene	442393	
	β-Sesquiphellandrene	12315492	
	Guaia-3,9-diene	585005	
	Dehydroaromadendrene	91746711	
	β--Elemene	6918391	
	β-Vatirenene	608753	
	Caryophyllene oxide	1742210	
	4-Methylbipheny	1392762	
	Spathulenol	92231	
	Hinesol	10878761	[3]
	Eudesm-4(14)-en-11-ol	91457	
	Atractylon	3080635	
	Bulnesol	90785	
	3-(2-Naphthyl)acrylaldehyde	5374806	

	Longiverbenone	530428	
	Germacrene B	5281519	
	Ledene oxide-(II)	534497	
	Aristolene epoxide	535269	
	Vellereal	178102907	
	Isolongifolene, 4,5-dehydro-	583154	
	Atractylenolide I	5321018	
	Eudesma-5,11(13)-dien-8,12-olide	327378	
	Atractylenolide III	155948	
	Oligosaccharides DP3	439531	[4]
	Oligosaccharides DP8		
	β-Myrcene	31253	[5]
	β-Sesquiphellandrene	12315492	
	β-Vatirenene	6431532	
	β-Eudesmol	91457	
	α-Phellandrene	7460	
	α-Bulnesene	94275	
	α-Bisabolol	442343	
	ɣ-Elemene	6432312	
	ɣ-Eudesmol	6432005	
	δ-Selinene	520383	
	(E)-3-caren-2-ol	576906	
	6,6-Dimethyl-2-methylene-(1S)	12314319	
	1,3,6-Octatriene, 3,7-dimethyl-, (Z)-	5320250	
	(+)-4-Carene	530422	
	4,4,6,6-Tetramethyl-bicyclo[3.1.0]hex-2-ene	578424	
	5-(Hydroxymethyl)-2-furancarboxaldehyde	237332	
	2-Methyl-5-(1-methylethyl)-, monoepoxide1,3-cyclohexadiene	527424	
	2-Methoxy-4-methyl-1-(1-methylethyl)-benzene	14104	
	1-Methoxy-4-methyl-2-(1-methylethyl)-benzene	161716	
	3,7-Dimethyl-, 1,6-cctadien-3-ol	67179	
	(Z)-3-decen-1-ol	5352846	
	Thymol	6989	
	Bornyl acetate	93009	
	3,7-Dimethyl-, methyl ester2,6-octadienoic acid	5365912	
	(−)-Myrtenyl acetate	61262	
	4-(2,2-Dimethyl-6-methylenecyclohexyl)-3-buten-2-one	6437590	
	1R,4R,7R,11R-1,3,4,7-Tetramethyltricyclo[5.3.1.0(4,11)]undec2-ene	600371	

	Cephreine	9017	
	Thujopsene-I3	577252	
	3,7-Dimethyl-, acetate, (Z)-2,6-octadien-1-ol	1549025	
	Longifolene-(V4)	570529	
	3,7-Dimethyl-, acetate, (E)-2,6-octadien-1-ol	1549026	
	3-Decen-1-ol-acetate	5363204	
	Longifolene-I2	565079	
	Bicyclo[2.2.2]octa-2,5-diene,1,2,3,6-tetramethyl	590286	
	Isoledene	530426	
	Caryophyllene	1742210	
	1,6,10-Dodecatriene, 7,11-dimethyl-3-methylene-, (Z)-α Caryophyllene	5281517	
	2-Isopropenyl-4a,8-dimethyl1,2,3,4,4a,5,6,7-octahydronaphthalene	605019	
	Seychellene	519743	
	Aromadendrene	91354	
	3,3,7,11-Tetramethyltricyclo[6.3.0.0(2,4)]undec-8-ene	579031	
	Patchoulene	101731	
	Atractylone	3080635	
	Selina-4(14)-7(11)diene-8-one	13986099	
	4-Styrylpyridazine	5371671	
	1-Pentadecanol	12397	
2. Azadirachta indica	Thiazole, 4,5-dihydro-2-methyl-	16867	[6]
	2-Hexenoic acid	5282707	
	2-Fluoro-5-methoxypyrimidine	581840	
	4H-Pyran-4-one, 2,3-dihydro-3,5-dihydroxy-6-methyl-	119838	
	Isopropyl isothiocyanate	75263	
	N-Aminopyrrolidine	27946	
	Benzofuran, 2,3-dihydro-	10329	
	D-Alanine, N-allyloxycarbonyl-, decyl ester	6422695	
	2(1H)Pyrimidinone,4-amino-1,N-dimethyl-	138689	
	2,6-Octadienal, 3,7-dimethyl-, (Z)-	643779	
	Geraniol	637566	
	benzamide	2331	
	Malic Acid	525	
	2-Methoxy-4-vinylphenol	332	
	1H-Cycloprop[e]azulene, 1a,2,3,4,4a,5,6,7b-octahydro1,1,4,7-tetramethyl-, [1aR-(1a.alpha.,4.alpha.,4a.beta.,7b.alpha.)]-	521243	

	trans-Cinnamic acid	444539	
	gamma.-Elemene OR γ-Elemene	6432312	
	2-Hydroxy-1-(1'-pyrrolidiyl)-1-buten-3-one	57977943	
	L-Proline, 1-acetyl-	66141	
	Dodecanoic acid	3893	
	Cyclohexane, 1-ethenyl-1-methyl-2-(1-methylethenyl)-4-(1- methylethylidene)-	94254	
	Fumaric acid, cyclobutyl ethyl ester	91713060	
	Phosphine, methyl(1-methylethyl)phenyl	580201	
	Carbamic acid, methylphenyl-, ethyl ester	75802	
	Ethyl .alpha.-d-glucopyranoside	91694274	
	.beta.-D-Glucopyranoside, methyl	445238	
	d-Glycero-l-gluco-heptose	21139463	
	2(1H)-Pyrimidinone, 5-methyl	170501	
	Sorbitol	5780	
	Piperidine, 1-(1-pentenyl)-	5371371	
	Galactitol	11850	
	Cyclohexane, 1,5-diisopropyl-2,3-dimethyl	566181	
	Palmitoleic acid	445638	
	n-Hexadecanoic acid	985	
	11-Oxa-tricyclo[4.4.1.0(1,6)]undecan-2-ol	573905	
	Hexadecanoic acid, ethyl ester	12366	
	Heptadecanoic acid	10465	
	3-Heptanol, 3,5-dimethyl-	140546	
	Phytol	5280435	
	9,12,15-Octadecatrienoic acid, (Z,Z,Z)-	5280934	
	Octadecanoic acid	5281	
	Ethyl 9,12,15-octadecatrienoate	5367460	
	Octadecanoic acid, ethyl ester	8122	
	Naphtho[2,1-b:7,8-b']difuran, 1,2,9,10-tetrahydro-2,9- dimethyl	617643	
	1-Heneicosyl formate	545651	
	Benzyl .beta.-d-glucoside	188977	
	Z,Z-8,10-Hexadecadien-1-ol acetate	5363392	
	Eicosanoic acid	10467	
	Methyl 19-methyl-eicosanoate	4580601	
	(1S,15S)-Bicyclo[13.1.0]hexadecan-2-one	13760785	
	Cyclotetradecane, 1,7,11-trimethyl-4-(1-methylethyl)-	15702	
	Eicosane	8222	
	Hexadecanoic acid, 2-hydroxy-1-(hydroxymethyl)ethyl ester	123409	
	Glycerol 1-palmitate	14900	
	Bis(2-ethylhexyl) phthalate	8343	
	Docosanoic acid	8215	
	Nonadecanoic acid, ethyl ester	29008	

	Cyclopentadecanone, 2-hydroxy-	543400	
	9,12,15-Octadecatrienoic acid, ethyl ester, (Z,Z,Z)-	5367460	
	Ethanol, 2-(octadecyloxy)-	75050	
	Linolenic acid, 2-hydroxy-1-(hydroxymethyl)ethyl ester (Z,Z,Z)-	5367459	
	Benzene, 1,2-dimethoxy-4-nitro-	69728	
	Fumaric acid, pent-4-en-2-yl tridecyl ester	91694892	
	Octacosane	12408	
	Squalene	638072	
	Nonacosane	12409	
	Octacosyl acetate	176972	
	1-Nonadecene	29075	
	Tetracosane	12592	
	Triacontyl acetate	3084839	
	.gamma.-Tocopherol	92729	
	Vitamin E	14985	
	Octadecane	11635	
	Pregn-4-ene-3,20-dione, 16-hydroxy-, (16.alpha.)-	243761	
	2,6,10,14-Tetramethyl-7-(3-methylpent-4-enylidene) pentadecane	91694785	
	Campesterol	173183	
	Stigmasterol	5280794	
	4-Cyclohexene-1,2-dicarboximide, N-butyl-, cis	91733922	
	gamma.-Sitosterol	133082557	
	Eicosane	8222	
	4,22-Stigmastadiene-3-one	5364563	
	D:A-Friedoursan-3-one	579869	
	Stigmast-4-en-3-one	5484202	
	Hexahydropyridine, 1-methyl-4-[4,5-dihydroxyphenyl]-	610035	
	Cannabidiol	644019	
	1H-1,2,4-Triazole-5(4H)-thione, 4-allyl-3-(3-furyl)-	723710	
	1,2-Bis(trimethylsilyl)benzene	519794	
	Pyrido[2,3-d]pyrimidine, 4-phenyl-	610177	
	2-(Acetoxymethyl)-3-(methoxycarbonyl)biphenylene	610255	
	Hexadecanoic acid, methyl ester	8181	[7]
	n-Hexadecanoic acid	985	
	9-Octadecenoic acid (Z)-	445639	
	9-Octadecenoic acid (Z)-, methyl ester	5364509	
	Methyl stearate	8201	
	Agaricic acid	12629	
	(E)-9-Octadecenoic acid ethyl ester	5364430	
	6-Octadecenoic acid, (Z)-	5281125	

	9-Octadecenamide	1930	
	Oleic Acid	445639	
	Heneicosane	12403	
	Tetratetracontane	23494	
	9- Octadecenamide	1930	
	Pentacosane	12406	
	Squalene	638072	
	Tetracontane	20149	
	γ-Tocopherol	92729	
	dl. α-Tocopherol	14985	
	4,5-Secocholest-6-en-4-oic acid, 5-oxo-	543971	
	2-[2-(6,6-Dimethylbicyclo[3.1.1]hept-2-en-2-yl) ethyl]-6,6-dimethylbicyclo [3.1.1]hept-2-ene	109294	
	Ergosta-5,7,22-trien-3-ol, acetate, (3. β.,22E)-	6436903	
	16alpha-Hydroxy-4-pregnene-3,20-dione	540627	
	γ-Sitosterol	133082557	
	1-Hydroxy-3,5,5,8,8-pentamethyl-5,6,7,8-tetrahydro-2- naphthalenecarbaldehyde	6195	
	Kryptogenin dioxime	9601637	
	Stigmasta-5,22-dien-3-ol, acetate, (3. β.)-	6437330	
	9-Hexadecenoic acid, 9-octadecenyl ester, (Z, Z)-	5364677	
	1-Eicosanol	12404	
	Lupa-13(18),20(30)-dien-3-yl acetate	585765	
	Methyl-8-methyl-nonanoate	20619411	
	pentadecanoic acid	13849	[8]
	hexadecenoic acid	5282743	
	9-octadecanoic acid (Z)-methyl ester	5364509	
	ç-Linolenic acid, methyl ester	5319706	
	eicosanoic acid, methyl ester	14259	
	docosanoic acid, methyl ester	13584	
	8-Octadecane	5460372	
	3-ethyl-5-(2 ethylbutyl)	292285	
	Gallic acid	370	
	Caffeic acid	689043	
	Syringic acid	10742	
3. Balanites aegyptiaca	Caffeic acid	689043	[9]
	Ferulic acid	445858	
	Gentisic acid	3469	
	p-Coumaric acid	637542	
	Sinapic acid	637775	
	Syringic acid	10742	
	Vanillic acid	8468	
	2-methoxy-4-vinylphenol	332	

	2,6-dimethoxyphenol	7041	
	2-methoxy-3(-2-propenyl)- phenol	596373	
	2-methoxy-4-(1-propenyl)- phenol	1715136	
	2,4-di-tert-butyl-phenol	7311	
	2,6-di-tert-butyl-phenol	31405	
	3-hydroxy-1-(4-hydroxy-3-methoxyphenyl)-1- propanone	75142	
	Kaempferol	5280863	
	Myricetin	5281672	
	Quercetin	5280343	
	Isorhamnetin	5281654	
	Hyperoside	5281643	
	Quercitrin	5280459	
	Quercetin 3-glucoside (isoquercetin)	5280804	
	Quercetin 3-rutinoside (Rutin)	5280805	
	Isorhamnetin-3-O-glucoside	5318645	
	Isorhamnetin 3-rutinoside	5481663	
	Isorhamnetin 3,7-diglucoside	44259357	
	Isorhamnetin 3-O-robinobioside	5491808	
	Isorhamnetin 3-O-galactoside	5318644	
	Epicatechin O-glucoside	122706219	
	Bergapten	2355	
	Marmesin	334704	
	N-trans-Feruloyltyramine	5280537	
	N-cis-feruloyltyramine	6440659	
	Trigonelline	5570	
	Diosgenin	99474	
	Yamogenin	441900	
	6-Methyldiosgenin	91873636	
	Rotenone	6758	
	β-Sitosterol	222284	
	Cholesterol	5997	
	Campesterol	173183	
	Stigmasterol	5280794	
	Pregn-5-ene-3β,16β,20(R)- triol 3-O-(2,6-di-O-α-Lrhamnopyranosyl)-β-Dglucopyranoside	131753107	
	Balanitin 3	101635466	
	Balanitin 4	44576180	
	Balanitin 5	44576181	
	Balanitin 6	44576182	
	Balanitin 7	44576183	
	Deltonin	441884	
	β-D-xylopyranosyl-(1→3)-βD-glucopyranosyl-(1→4)[α-Lrhamnopyranosyl-(1→2)]-βD-glucopyranoside	70699388	

	Balanitesin	02316758	
	Balanitoside	150711	
	β-Sitosterol glucoside	5742590	
	Stigmasterol-3-O-β-Dglucopyranoside	12895778	
	Undecane	14257	[10]
	Dodecane	8182	
	Tetradecane	12389	
	Pentadecane	12391	
	Hexadecane	11006	
	heptadecane	12398	
	Octadecane	11635	
	Nonadecane	12401	
	Eicosane	8222	
	Heneicosane	12403	
	Docosane	12405	
	Tricosane	12534	
	Tetracosane	12592	
	Pentacosane	12406	
	Hexacosane	12407	
	Octacosane	12408	
	Cholesterol	5997	
	Campesterol	173183	
	Stigmasterol	5280794	
	beta-Sitosterol	222284	
	Lauric C12:0	3893	
	Myristic C14:0	11005	
	Palmitic C16:0	985	
	Palmitoleic C16:1	445638	
	Stearic C18:0	5281	
	Oleic C18:1	445639	
	Linoleic C18:2	5280450	
	Arachidic C20:0	10467	
	Arachidonic C20:1	444899	
	Behnic C22:0	8215	
	Erucic C22:1	5281116	
	n-nonanal	31289	[11]
	(*E*)-2-decenal	5283345	
	(*E,Z*)-2,4-decadienal	6427087	
	(*E,E*)-2,4-decadienal	5283349	
	palmitic acid	985	
	ethyl palmitate	12366	
	linoleic acid	5280450	
	(*E*)-9-octadecenoic acid	637517	
	ethyl 9(*E*),12(*E*)-octadecadienoate	5365672	
	ethyl oleate	5363269	

	eicosanoic acid	10467	
	13(*Z*)-docosenoic acid	5281116	
	docosanoic acid	8215	
	13-docosynoic acid	232555	
	diosgenin	99474	
	yamogenin	441900	
	3,5-spirostadiene	337494	
	glycyrrhizic acid	14982	
	hecogenin acetate	101906	
	3,4,5-Trimethoxycinnamic acid	735755	[12]
	Pentadecanoic acid	13849	
	Endo-borneol	6552009	
	Linoleic acid	5280450	
	3,4-Dimethoxy-2, hydroxychalcone	5712116	
	7-Hydroxy-3-(4-methoxyphenyl)-4-methylcoumarin	5357627	
	17-Octadecynoic acid	1449	
	Butanoic acid, 3-hydroxy-	441	
	Undecanoic acid	8180	
	Lactose	6134	
	Oleic acid	445639	
	4-Mercaptophenol	240147	
	Hexa-hydro-farnesol	138824	
	Nonanoic acid	8158	
	Palmitic acid	985	
	Elcosanoic acid	10467	
	a-Methylcaproic acid	20653	
	Stearic acid	5281	
	Heptadecanoic acid	10465	
	Erucic acid	5281116	
	Vitexin	5280441	
	2-Hexadecanol	85779	
	Geranyl isovalerate	5362830	
	Stigmasterol	5280794	
	3-(3,4-Dimethoxyphenyl)-4-methylcoumarin	688794	
	Glyceryl monooleate	5283468	
	1-Tricosanol	18431	
	(S)-(-)-Citronellic acid	6999955	
4. Berberis vulgaris	2-Pentanone, 4-Hydroxy-4-Methyl-	31256	[13]
	Propanal, 3-Ethoxy-	17755	
	trans-2-Pentenoic acid	638122	
	Acetic acid, Phenyl ester	31229	
	6-Nonynoic acid	534311	
	Benzyl alcohol	244	

	Benzene, 1,3,5-Trimethyl-	7947	
	Phenol, 2-Methoxy	460	
	Phenylethyl Alcohol	6054	
	Ribitol	6912	
	3-(2-Hydroxyphenyl) acrylic acid	637542	
	Octadecanoic acid, 3-hydroxy-, methyl ester	538801	
	α-Ylangene	6432119	
	7,10-Pentadecadiynoic acid	30942	
	2-Methoxy-4-vinylphenol	332	
	α -Longipinene	520957	
	2-Propen-1-Ol, 3-Phenyl	308	
	α-Curcumene	442360	
	trans-Sesquisabinene hydrate	6428444	
	α-Bulnesene	520826	
	Sesquicineole	341779	
	2-Propen-1-Ol, 3-Phenyl-, Acetate	5282110	
	Aromadendrene oxide	528759	
	Methyl 5,7-hexadecadiynoate	14957560	
	Guaiol	227829	
	α-Guaiene	107152	
	cis-5,8,11,14,17-Eicosapentaenoic acid	446284	
	2-Pentanone, 4-Hydroxy-4-Methyl-	31256	
	2-Butenedioic acid	444972	
	3-Thujanol	527259	
	4-Heptenal	5283318	
	2,4-Heptadienal, (E,E)	5283321	
	2-Nonen-1-ol, (E)	5364941	
	4-Penten-2-Ol, 3-Methyl-	544701	
	2-Decenal, (E)-	5283345	
	Benzaldehyde, 4-(1-methylethyl)-	326	
	α-Ylangene	6432119	
	1,4-p-Menthadien-7-al	6429112	
	2,4-Decadienal, (E,E)-	5283349	
	2-Undecenal, E-	5283356	
	Caryophyllene	5281515	
	Methyleugenol	7127	
	Benzene,1-(1,5-Dimethyl-4-Hexenyl)- 4-Methyl-	92139	
	2,6,10-Dodecatrien-1-ol,3,7,11- trimethyl-	445070	
	7-epi-cis-Sesquisabinene hydrate	6428435	
	1,2,3-Benzenetriol (Pyrogallic acid)	1057	
	cis-Z-α-Bisabolene epoxide	91753574	
	4-Vinylguaiacol	332	[14]
	D-Mannoheptulose	12600	

	1-Amino-2-(hydroxymethyl)anthraquinone	32208	
	Benzothiazole, 2-methyl	8446	
	Benzaldehyde, 2-{(1-(2-{[1-(2aminophenyl)methylidene] amino}phenyl)methylidene]amino}	629920	
	Tetradecanoic acid	11005	
	Di-(2-ethylhexyl)phthalate	8343	
	1,2-Benzenedicarboxylic acid, diisooctyl ester	33934	
	Tetracosanoic acid, methyl ester	75546	
	1,2-Benzenedicarboxylic acid, diisononyl ester	590836	
	Epi-ligulyl oxide	91748045	
	Resorcinol	5054	[15]
	Gallic acid	370	
	Catechin	9064	
	Chlorogenic acid	1794427	
	Berberine	2353	
	Rosmarinic acid	5281792	
	Syringic acid	10742	
	P-coumaric	637542	
	Ferulic acid	445858	
	O-coumaric	10046064	
	Protocatechuic	287064	
	Caffeic acid	689043	
	Apigenin	5280443	
	Luteolin	5280445	
	Kaempferol	5280863	
	Rutin	5280805	
	Myricetin	5281672	
	Quercetin	5280343	
	Vanillic	8468	
5. Bidens pilosa	Phenylheptatriyne (1-phenylhepta-1,3,5-triyne)	77981	[16]
	6-Phenylhexa-1,3,5-triyn-1-ol	21441162	
	6-Phenylhexa-1,3,5-triyn-1-yl acetate	15590	
	Trideca-1,11-diene-3,5,7,9-tetrayne	5322026	
	Trideca-2,12-diene-4,6,8,10-tetrayn-1-ol	14729085	
	Trideca-2,12-diene-4,6,8,10-tetrayn-1-yl acetate	14729084	
	Tridec-1-ene-3,5,7,9,11-pentayne	441552	
	b-D-Glucopyranosyloxy-3-hydroxy-6(E)-tetradecen-8,10,12-triyne	102506428	
	1,2-Dihydroxytrideca-5,7,9,11-tetrayne	11264113	
	1,3-Dihydroxy-6(E)-tetradecene-8,10,12-triyne	11195290	

	1-Phenyl-hept-5t-ene-1,3-diyne	5281154	
	7-Phenyl-hepta-4,6-diyn-1,2-diol	ChemSpider ID4942347	
	7-Phenyl-hepta-4,6-diyne-2-ol	21593834	
	7-Phenyl-hepta-2,4,6-triyn-2-ol	54376451	
	7-Phenyl-hepta-4,6-diyn-2-ol	11645346	
	5-(2-Phenylethynyl)-2-thiophene methanol	2822242	
	(6E,12E)-3-Oxo-tetradeca-6,12-dien-8,10-diyn-1-ol	21769349	
	1,2-Dihydroxy-5(E)-tridecene-7,9,11-triyne	101414046	
	2-O-b-D-Glucosyltrideca-11E-en-3,5,7,9-tetrayn-1,2-diol (tetrayne)	11264113	
	(R)-1,2-Dihydroxytrideca-3,5,7,9,11-pentayne	15081391	
	(2E)-7-Phenylhept-2-ene-4,6-diyn-1-yl acetate	89297	
	(11E)-Trideca-1,11-diene-3,5,7,9-tetrayne	5322026	
	(2E)-Trideca-2,12-diene-4,6,8,10-tetrayn-1-ol	14729085	
	2E)-Trideca-2,12-diene-4,6,8,10-tetrayn-1-yl acetate	14729084	
	Trideca-3-11-diene-5-7-9-triyne-1-2-diol	6123027	
	Tridec-5-ene-7,9,11-triyne-3-ol	5352706	
	Astragalin	5282102	
	Axillaroside	44259807	
	Apigenin 7-O-glucoside	5280704	
	Rutin	5280805	
	Querciturone	5274585	
	Centaurein	5489090	
	Jacein	11577257	
	Quercetin-3-O-a-L-rhamnosyl (1 ? 6)-b-D-galactoside	44259105	
	Quercetin 3-O-b-D-glucopyranoside	5280804	
	Luteoside	72188972	
	Luteolin 7-O-b-D-glucopyranoside	13093777	
	Quercetin 3-O-glucoside	5280804	
	Quercetin 3-O-b-D-galactopyranoside	5281643	
	Quercetin 3-O-rabinobioside	10371536	
	Quercetin 3-O-rutinoside	124221768	
	Sulfuretin	5281295	
	6,7,3' ,4' -Tetrahydroxyaurone	42607747	
	Apigenin	5280443	
	Butein	5281222	
	Okanin	5281294	
	Centaureidin	5315773	
	Digitoflavone (Luteolin)	5280445	

	Quercetin-3,3' -dimethoxy-7-O-a-L-rhamnopyranosyl- (1 ? 6)-b-D-glucopyranoside	25230796	
	Quercetagetin 3,6,3' -trimethyl ether	5464461	
	5-O-Methylhoslundin	15726099	
	Benzoic acid	243	
	Caffeic acid	689043	
	Chlorogenic acid	1794427	
	3,4-di-O-Caffeoylquinic acid	5281780	
	3,5-di-O-Caffeoylquinic acid	21769778	
	4,5-di-O-Caffeoylquinic acid	6474309	
	Neochlorogenic acid	5280633	
	4-O-Caffeoylquinic acid	9798666	
	Dimethoxyphenol	78828	
	Eugenol	3314	
	Ethyl caffeate	317238	
	Ferulic acid	445858	
	Gallic acid	370	
	iso-Vanillin	12127	
	2-O-Caffeoyl-2-C-methyl-D-erythronic acid	23955864	
	p-Coumaric acid	637542	
	Pyrocatechin	289	
	p-Hydroxybenzoic acid	135	
	Protocatechuic acid	72	
	p-Vinylguaiacol	332	
	Salicylic acid	338	
	Tannic acid	16129778	
	2-Phenyl-ethanol	6054	
	2-Hydroxy-6-methylbenzaldehyde	585174	
	4-Ethyl-1,2-benzenediol	70761	
	3-O-Caffeoyl-2-C-methyl-D-erythrono-1,4-lactone	168356045	
	Camphene	6616	
	(E)-b-Ocimene	5281553	
	m-Cymol	10812	
	Myrcene, b-Myrcene	31253	
	Limonene	22311	
	Perillene	68316	
	Sabinene	18818	
	trans-Pinocarveol	88302	
	Terpinolene	11463	
	(Z)-b-Ocimene	5320250	
	gamma-Terpinene	7461	
	alpha--Pinene	6654	
	alpha--Phellandrene	7460	
	Beta-Pinene	440967	

	Beta-Phellandrene	11142	
	Beta-trans-Ocimene	5281553	
	Beta-cis-Ocimene	5320250	
	3-Carene	26049	
	(4E,6Z)-2,6-Dimethyl-2,4,6-octatriene	5371125	
	Borneol	64685	
	cis-Verbenol	164888	
	Linalool, b-Linalool	6428573	
	p-Cymen-8-ol	14529	
	Terpinen-4-ol	11230	
	Trans-Verbenol	89664	
	a-Terpineol	17100	
	1,8-Cineole	2758	
	4-Terpineol	11230	
	Acorenone B	21674978	
	allo-Aromadendrene	42608158	
	Bicyclogermacrene	13894537	
	E-caryophyllene	20831623	
	(+)-Epi-bicyclosesquiphellandrene	521496	
	cedr-8(15)-en-9-α-ol	119831	
	(+)-Cyclosativene	519960	
	z-c-Bisabolene	91753504	
	Daucene	177773	
	Epi-10- .Gamma.-Eudesmol	6430754	
	epi-Longipinanol	91746617	
	Epoxy alloaromadendrene	91746712	
	Elixene	94254	
	Farnesene, (E)-b-Farnesene	5281517	
	Germacrene A	9548706	
	Germacrene-D	91723653	
	Humulene oxide II	91749531	
	Intermedeol	15560333	
	Isoledene	530426	
	Selina-3,11-Dien-6.Alpha.-Ol	91746575	
	Selina-3,7(11)-diene	522296	
	trans-Calamenen-10-ol	10798883	
	trans-a-Bergamotene	405230201	
	Valencene	9855795	
	(Z)-Gamma-Bisabolene	3033866	
	beta-Cedrene	11106485	
	beta-Selinene	442393	
	Alpha-Cadinol	10398656	
	Alpha-Calacorene	12302243	
	Alpha-Bergamotene	86608	
	Alpha-Copaene	19725	
	Alpha-Caryophyllene	5281520	

	Alpha-Cubebene	442359	
	Alpha-Gurjunene	15560276	
	Alpha-Humulene	5281520	
	Alpha-Muurolene	12306047	
	Alpha-Ylangene	442409	
	beta-Bourbonene	62566	
	beta-Bisabolene	10104370	
	beta-Caryophyllen	5281515	
	beta-Cubebene	93081	
	(-)-beta-Cadinene	10657	
	beta-Elemene	6918391	
	beta-Gurjunene	6450812	
	Gamma-Cadinene	92313	
	Gamma-Muurolene	12313020	
	epsilon-Muurolene	520461	
	epi-α-Cadinol	6432221	
	epsilon-Cadinene	12302130	
	delta-Elemene	12309449	
	delta-Cadinene	441005	
	1-epi-Cubenol	519857	
	14-Oxy-α-muurolene	91746681	
	14-Hydroxy-deltacadinene	14845382	
	Caryophyllene oxide	1742210	
	Epi-cedrol	6713078	
	(E)-nerolidol; trans-Nerolidol)	5284507	
	Precocene 1	28619	
	Spathulenol	92231	
	T-Muurolol	3084331	
	Pimaradiene	440909	
	Phytol	5280435	
	Phytenic acid	12314226	
	Sandaracopimara-8(14),15-diene	443469	
	1-Eicosene	18936	
	Friedelin	91472	
	Friedelan-3 b-ol	101341	
	Lupeol	259846	
	Lupeol acetate	92157	
	Squalene	638072	
	b-Amyrin	73145	
	B-Carotene	5280489	
	Acetal	7765	
	Bornyl acetate	93009	
	Caryophylla-4(14),8(15)-dien-5-ol	6428430	
	cis-3-Hexen-1-ol	5281167	
	cis-3-Hexenyl acetate	5363388	
	cis-Chrysanthenyl acetate	6431301	

	Diphenylenemethane	6853	
	(E)-Geranyl acetone	1713001	
	Hexadecanol	2682	
	Hexahydrofarnesylacetone	10408	
	Hexadecyl acetate	12393	
	Isophorone	6544	
	Megastigmatrienone	5375190	
	Mesitylene	7947	
	Methyl hexadecanoate	8181	
	Methyl linoleate	5284421	
	Muurol-5-en-4-one (cis-14-nor-)	12612678	
	n-Tricosane	12534	
	n-Decane	15600	
	n-Dodecane	8182	
	n-Docosane	12405	
	n-Tetradecane	12389	
	n-Hexadecane	11006	
	n-Heptadecane	12398	
	n-Heneicosane	12403	
	n-Octadecane	11635	
	n-Pentadecane	12391	
	Pentadecanal	17697	
	Octadecadieno	54327406	
	Nonanal	31289	
	Phenylacetaldehyde	998	
	Pseudocumene	7247	
	1-Heptadecene	23217	
	1-Octadecene	8217	
	2,5,9-Trimethylcycloundeca-4,8 dienone	21160296	
	6-Methyl-5-hepten-2-one	9862	
	Decanal	8175	
	Tridecane	12388	
	Aristophyll-C	162941351	
	Pheophytin a	135398712	
	(132 S)-132 -Hydroxypheophytin a	135466950	
	Behenic acid	8215	
	2-Butoxyethyl linoleate	87553426	
	Ethyl linoleate acid	5282184	
	Methyl linolenate	5319706	
	Linolenic acid	5280934	
	Capric acid	2969	
	Elaidic acid	637517	
	Myristic acid	11005	
	Lauric acid	3893	
	Linoleic acid	5280450	
	Palmitic acid	985	

	Palmitoleic acid	445638	
	Campestrol	312822	
	Daucosterol	5742590	
	Stigmasterol	5280794	
	b-Sitosterol	222284	
	5a-Stigmasta-7-en-3 b-o	6432515	
	5a-Stigmasta-7,22t-dien-3 b-ol	6432461	
	Aesculetin	5281416	
	Caffeine	2519	
	Precocene 1	28619	
	(E)-Butenedioic acid	444972	
	Butanedioic acid	1110	
	2-Butoxy ethanol	8133	
	a-Tocopheryl quinone	24205	
	1-(Thiophen-2-yl)-ethanone	5324551	
6. Bougainvillea spectabilis	Toluene	1140	[17]
	Methyl 2-methylbutanoate	13357	
	2-Methylpropanoic acid	6590	
	Butyl formate	11614	
	Hexanal	6184	
	Butyl acetate	31272	
	2-Furfural	7362	
	Ethyl (E)-crotonate	429065	
	(Z)-2-Hexenal	6428782	
	o-Xylene	7237	
	Heptanal	8130	
	cis-Linalool oxide	6428573	
	Terpinolene	11463	
	Linalool	6549	
	Ethyl 3-hydroxy-hexanoate	61293	
	Terpinen-4-ol	11230	
	Methyl salicylate	4133	
	trans-Dihydrocarvone	6432474	
	Verbenone	29025	
	Pulegone	442495	
	Dihydroedulan II	522517	
	α-Copaene	12303902	
	(E)-β-Damascenone	5366074	
	α-(E)-Ionone	638014	
	Aromadendrene	91354	
	Cadina-1.4-diene	6427091	
	Dodecanoic acid	3893	
	(Z)-3-Hexenyl salicylate	5371102	
	α-Santalol	11085337	
	(Z.Z)-Farnesol	1549107	

	Methyl linolenate	5319706	
	2-Heptadecanone	18027	
	Methyl hexadecanoate	8181	
	Ethyl hexadecanoate	12366	
	Isophytol	10453	
	Propyl hexadecanoate	75232	
	Phytol	5280435	
	Flavone	10680	[18]
	6-Methoxyflavone	147157	
	Phytol	5280435	
	1-Docosene	74138	
	1-Tricosene	181154	
	Phenol, 2,4-bis [1,1-dimethylethyl]	93344	
	Hexadecanoic acid, methyl ester	8181	
	1-Eicosene	18936	
	3,3' -Methylenebis(1,5,8,11-tetraoxacyclotridecane)	560722	[19]
	1-Butanol, 2,2-dimethyl	236682	
	3-Methyl-4-piperonyl-5-isoxazolone	584642	
	Oxime-, methoxy-phenyl-_	9602988	
	3-Pentanol, 2,3,4-trimethyl-	520484	
	N-(3-Nitrobenzylidene)-O-toluidine	292086	
	2,4-Di-tert-butylphenol	7311	
	Methyl(methyl 2-O-acetyl-3,4-di-O-methyl-. alpha.-D-mannopyranoside)uronate	91697221	
	Piperidin-4-ol, 2,5-dimethyl-1-(1,1-dimethylethyl)-	598385	
	3-O-Methyl-d-glucose	8973	
	Neophytadiene	10446	
	2-Pentadecanone, 6,10,14-trimethyl-	10408	
	Methyl 10-trans,12-cis-octadecadienoate	5471014	
	Phytol	5280435	
	Oleanitrile	6420241	
	Tetradecanamide	69492	
	9,10-Secoergosta-7,10(19),22-triene-3,5,6- triol, (3.beta.)-	21160013	
	Squalene	638072	
	(R)-6-Methoxy-2,8-dimethyl-2-((4R,8R)-4,8,12- trimethyltridecyl)chroman	67475966	
	Octadecane, 1,10 -oxybis-	129745941	
	16-Deoxokryptogenin	539138	
	.alpha.-Tocopherol	2116	
	.alpha.-Tocopheryl acetate	86472	
	Octadecane, 1,1’-[1,3-propanediylbis(oxy)]bis	624534	
	n-Propyl 9-octadecenoate	5356106	

	Stigmasterol	5280794	
	Sandaracopimar-15-ene-8.beta.,11.alpha.-12.<br>beta.-triol	620176	
	Fucoxanthin	: 5281239	
	Stigmasta-7,25-dien-3-ol, (3.beta.,5.alpha.)-	6321372	
	Stigmast-7-en-3-ol, (3.beta.,5.alpha.,24S)-	5283639	
	Methyl 2-hydroxy-eicosanoic	3472786	
	Eicosane, 1,20-dibromo-	545761	
	2H-Azepin-2-one, hexahydro-1-(3,4,5,6-tetrahydro-2H-azepin-7-yl)-	591990	
7. Brassica juncea	2-Furanmethanol	22310	[20]
	1-Heptadecanol	15076	
	1-Phenyl ethanol	7409	
	Hexenal	10460	
	2-Furancarboxaldehyde	66349	
	Furfural	7362	
	Heptanal	8130	
	Octanal	454	
	9,17-Octadecadienal	6431297	
	Furaldehyde	7362	
	(E,E)-2,4-Nonadienal	129774274	
	Dodecanal	8194	
	Ethyl pentanoate	10882	
	Ethyl dodecanoate	7800	
	Ethyl linoleate	5282184	
	Ethyl hexadecanoic acid	12366	
	3-Deoxy-d-mannoic lactone	541561	
	2-Dodecen-1-yl(-)succinic anhydride	5362708	
	Ethyl hexanoate	31265	
	Ethyl benzoate	7165	
	Pentadecanoic ester	23518	
	Butyl acetate	31272	
	Tetradecanoic acid(Myristic acid)	11005	
	Octadecanoic acid (Stearic acid)	5281	
	n-Hexadecanoic acid(Palmitic acid)	985	
	9,12-Octadecadienoic acid methyl ester (Linoleic acid)	5280450	
	9-Octadecenoic acid (oleic acid)	445639	
	Gamolenic acid	5280933	
	3-Methyl-2-pentyl-2-cyclopenten-1-one	62378	
	1,3-Cyclopentanedione	77466	
	2,4-(1H,3H) pteridinedione	5374589	
	Cyclohexanone	7967	
	Cyclopropaneisothiocyanato	92463	
	4-Methylpentyl isothiocyanate	519452	

	Allylthiocyanate	69816	
	3-Butenyl isothiocyanate	76922	
	Buteneisothiocyanate	76922	
	2-Butyl isothiocyanate	78151	
	Allylisothiocyanate	5971	
	2-Phenylethylisothiocyanate	16741	
	Oxime-, methoxy-phenyl	9602988	
	α-Methyl-D-mannopyranoside	101798	
	β-D-Glucopyranoside	440936	
	α-D-Galactopyranoside	90657911	
	3,4-Altrosan	548229	
	4-Mercaptophenol	240147	
	γ-Sitosterol	457801	
	Stigmasterol	5280794	
	Stigmastan-3,5-diene	525918	
	β-Sitosterol	222284	
	4,5-Epithiovaleronitrile	100582	
	Benzenepropanenitrile	12581	
	Acetic acid	176	
	Tridecane	12388	[21]
	Pentadecane	12391	
	2,6-dimethyl-2-vinyl-5-heptenoic acid	567589	
	Allyl methyl sulfide	66282	
	Dimethyl disulfide	12232	
	Dimethyl trisulfide	19310	
	Dimethyl tetrasulphide	79828	
	2,6-dimethyl-2,6-octadiene	5365898	
	3-methyl-3-(1-naphthyl)-1-butene	71421054	
	Styrene	7501	
	4-phenyl-2-butenal	11008054	
	3-hexenal	643139	
	Hexanal	6184	
	Benzaldehyde	240	
	Benzeneacetaldehyde	998	
	a-ethylidene-benzeneacetaldehyde	20446	
	Octanal	454	
	2-octenal	5283324	
	Nonanal	31289	
	(E)-2-nonena	5283335	
	Decana	2771130	
	(Z)-2-decenal	5354834	
	Undecanal	8186	
	2-undecenal	5283356	
	b-cyclocitral	9895	
	Dodecanal	8194	

	2-dodecenal	5283361	
	Tetradecanal	31291	
	Nerylacetone	1713001	
	6-methyl-5-hepten-2-one	9862	
	2-nonen-4-one	448996	
	3,5-octadiene-2-one	181575	
	(E)-6,10-dimethyl-5,9-undecadien2-one	19633	
	b-ionone	638014	
	5,6-epoxide-b- ionone	5352481	
	5,5-dimethyl-4-phenyl-1,5-2Hpyrrol-2-one	12328969	
	Ethanol	702	
	1-pentanol	6276	
	Isoamylalcohol	31260	
	(Z)-2-penten-1-ol	5364919	
	1-hexanol	8103	
	Cyclohexanol	7966	
	2-ethyl hexanol	7720	
	(Z)-3-hexen-1-ol	5281167	
	1-heptanol	8129	
	1-octanol	957	
	1-dodecanol	8193	
	Phenylethanol	6054	
	4-ethyl-phenol,	31242	
	2-methoxy-4-vinylphenol	332	
	Butyl acetate	31272	
	3-methyl-1-butyl acetate	31276	
	3-hexenyl acetate	5352557	
	2-phenylethyl acetate	7654	
	Ethyl octanoate	7799	
	Methyl nonanoate	15606	
	Ethyl nonanoate	31251	
	Methyl decanoate	8050	
	Ethyl decanoate	8048	
	Ethyl phenylacetate	7590	
	Ethyl 3-phenylpropionate	16237	
	Methyl 10-methyl undecanoate	131982675	
	Methyl hexadecanoate	8181	
	Methyl tetradecanoate	31284	
	Methyl 14-methyl-hexadecanoate	56935907	
	Methyl 7,10,13-hexadecatrienoate	556196	
	Ethyl hexadecanoate	12366	
	Ethyl linoleolate	5282184	
	Methyl (Z,Z,Z)-9,12,15- octadecatrienoate	5319706	
	Dibutyl phthalate	3026	
	Allyl isothiocyanate	5971	

	Butyl isothiocyanate	11613	
	Isobutyl isothiocyanate	68960	
	1-isothiocyanato-3-methyl-butane	79086	
	(2-isothiocyanatoethyl)-benzene	140257	
	4-methyl-pentanenitrile	10956	
	3-butenenitrile	8009	
	Pentanedinitrile	10994	
	Benzenepropanenitrile	12581	
	2-pyrrolidinethione	2775267	
	2-sec-butylthiazole	519539	
	Methylnaphthalene	7002	
	2,6-dimethyl-naphthalene	11387	
	2-methoxy-3-(1-methylpropyl)- pyrazine	6951559	
8. Bridelia ferruginea	Î²-Amyrin	345510	[22]
	Î±-Amyrin	91746489	
	Olean-12-en-3-one	6454747	
	Î±-Amyrin acetate	345510	
	Lupenone	92158	
	Lupeol	259846	
	24-Methylenecycloartenol	91749291	
	Lupeol acetate	92157	
	2,3,6-Trimethylhept-3-en-1-ol	5365945	
	Citronellyl butyrate	8835	
	3,7-Dimethyl-2-octen-1-ol	5365836	
	p-Menth-4-en-3-one	107372	
	3Î²-Hydroxydamascone	92564	
	4-Hydroxy-3-methoxyphenyl-2-propenal	129702259	
	3,5-Dimethoxy-4-hydroxycinnamaldehyde	5280802	
	4-Phenylbenzophenone	75040	
	Cymene	7463	
	Phytol acetate	6428538	
	Phytol	5280435	
	Stigmasta-3,5-dien-7-one	12444466	
	Z-6-octadecenoic acid	91709816	
	4-Phenylbenzophenone	75040	
	3Î²-Acetyloxybufa-14,16,20,22-tetraenolide	634233	
	Lupeol	259846	
	Î²-Amyrin	345510	
	Î±-Amyrin acetate	92156	
	Tocopherol acetate	86472	
	Stigmasterol	5280794	
	Sitosterol	222284	
	Benzoic acid, methylester	7150	

	1-Ethyl-2-methyl benzene	21336804	[23]
	Curcumene	92139	
	Feruloyl	16119330	
	3-Tert-butyl-4-hydroxyanisole	8456	
	Vanillin	1183	
	Myricetin	5281672	
	2,4-di-tert-butylphenol	7311	
	Coumaran	10329	
	Benzene, 1,4-dimethoxy	66639	
	Phytol	5280435	
	Zingiberene	92776	
	Kaempferol	5280863	
	Benzoic acid, 3,4-dimethoxy-, methyl ester	16522	
	Ylangene	20055075	
	Quercetin	5280343	
	Tetradecanoic acid	11005	
	Azulene	9231	
	Caryophyllene oxide	1742210	
	1,2-benzenedicarboxylic acid	1017	
	n-Hexadecanoic acid	985	
	Trans-Octadec-9-enoic acid	637517	
	Tetratriacontane	26519	
	Pentatriacontane	12413	
	lutein	5281243	
	vomifoliol	5280462	
	corilagin	73568	
	kaempferide-3-*O*-*β*-ᴅ-glucoside	90659152	
	myricetin	5281672	
	isomericitrin	5280459	
	myricitrin	5281673	
	quercitrin	5280459	
	rutin	5280805	
	β-sitosterol glucoside	5742590	
	oleic acids	5280590	
	9-Octadecenoic acid	637517	[24]
	n-Hexadecanoic acid	985	
	Methyl-11-octadecenoate	5364432	
	cis-13-Eicosenoic acid	5312518	
	cis-11-Eicosenoic acid, methyl ester	5463047	
	Methylhexadecanoate	520159	
	Eicosanoic acid	10467	
	Octadecanoic acid	5281	
	.gamma.-Sitostero	457801	
	Methyl 18-methylnonadecanoate	530340	

	13-Docosenoic acid, methyl ester, (Z)-	5364423	
	alpha.-Amyrin	73170	
	Methyl stearate	8201	
	Stigmasterol	5280794	
	2,3-Dihydroxypropyl elaidate	5364833	
	Monomethylnonanedioate	75009	
	Erucic acid	5281116	
	Tetradecanoic acid	11005	
	13-Tetradecenal	522841	
	Docosanoic acid	8215	
	Palmitoleic acid	445638	
	Monomethylsuberate	554191	
	Disparlure	205983	
	Dodecanoic acid	3893	
	Stigmast-4-en-3-one	5484202	
	Methyl-3-(4-hydroxy-3-methoxyphenyl)-2-Propenoate	523498	
	Lup-20(29)-en-3-one	92158	
	Docosanoic acid, methyl ester	13584	
	Vanillin	1183	
	Methyl 2-octylcyclopropene-1-octanoate	115261	
	Tricosanoic acid	17085	
	Heptadecanoic acid	10465	
	10-(2-Hexylcyclopropyl) decanoic acid	543833	
	9-Eicosenoic acid, (Z)-	5282767	
	Tetracosanoic acid, methyl ester	75546	
	Friedelan-3-one	91472	
	Methyl-9-Hexadecenoate	14258	
	Methyl-15-methyl-hexadecanoate	522345	
	beta.-Amyrin	73145	
	9-Octadecenamide, (Z)-	5283387	
	Cyclopropaneoctanoic acid, 2-octyl-, methyl ester	543406	
	10-Heptadecenoic acid	5312434	
	1-Hexacosanol	68171	
	Tricosanoic acid, methyl ester	75519	
	9,12-Octadecadienoic acid (Z,Z)-	5280450	
	Campesterol	173183	
	Pentadecanoic acid	13849	
	Dimethyl nonanedioate	15612	
	Triacontyl acetate	3084839	
	Methyl tetradecanoate	31284	
	cis-9-Octadecenoic acid, propyl ester	5356106	
	4-Hydroxy-3,5-dimethoxybenzaldehyde	8655	
	2-Methoxy-1,7,7-trimethyl bicyclo[2.2.1]heptanes	95330	
	Tetracosanoic acid	11197	

	Cholesterol	5997	
	Hexacosanoic acid, methyl ester	22048	
	Dimethyloctanedioate	44597715	
	Methylpentadecanoate	13072095	
	(+)-Sesamin	72307	
	cis-9-Tetradecenoic acid, heptyl ester	87382856	
	Hexacosanoic acid	10469	
	2-(Tetradecyloxy) ethanol	3015324	
	Pantolactone	439368	
	2-Methoxy-4-vinylphenol	332	
	Methyl 22-methyl-tetracosanoate	15047974	
	Diisopropylaminoacrylonitryl	5368921	
	Methyl pentadecyl ether	542191	
	1,1,6-Trimethyl-3-methylene-2-(3,6,9,13-tetramethyl-6-ethenye-10,14-dimethylene-pentadec-4-enyl)cyclohexane	91700552	
	3,4-Dihydro-8-hydroxy-3-methyl-1H-2-Benzopyran-1-one	28516	
	i-Propyl 11-octadecenoate	91692607	
	4-Hydroxy-3-methoxy-benzoic acid methyl este	19844	
	Nonanoic acid	8158	
	6-Octadecenoic acid	5282754	
	Octanoic acid	379	
	Methyl-9-octadecenoate	8202	
	Z-10-Tetradecen-1-ol acetat	5363221	
	13-Octadecenal, (Z)-	5364497	
	Hexanoic acid	8892	
	4,9-Dipropyl-dodecane	283509	
	Methyl-14-methyl-Pentadecanoate	21205	
	,6-Dimethyl-8-(tetrahydropyran-2-yloxy)-octa-2,6-dien-1-ol	5367918	
	Heptadecanolide	3639916	
	2-Decenal, (E)-	5283345	
	is-10-Heptadecenoic acid, methyl ester	6029464	
	6-Isopropenyl-4,8a-dimethyl-4a,5,6,7,8,8a-hexahydro-1H-naphthalen-2-one	594234	
	Nonanal	31289	
	Dimethyl nonanedioate	15612	
	6,10-Dimethyl-9-Undecen-2-one	102604	
	Ethyl chloroacetate	7751	
	2-Butoxyethanol	8133	
	2-Octenal, (E)-	5283324	
	trans-Isoeugenol	853433	
	2,3-Epoxyheptane	26970	
	Cyclohexanone	7967	
	p-Tert-butylphenol	7393	

	1,7,7-Trimethyl-bicyclo [2.2.1]heptan-2-one	3821472	
	1,7,7-Trimethyl-bicyclo [2.2.1]heptane-2,3-Diol	131846009	
	7-(1-Methylethylidene)-bicyclo [4.1.0]heptanes	40707	
	Dimethyl heptanedioate	74416	
	Decanedioic acid, dimethyl ester	7829	
	9-Octadecenoic acid, 2,3-dihydroxypropyl este	5283468	
	Propanenitrile, 3-(5-diethylamino-1-methyl-3-pentynyloxy)-	610055	
	Cyclopentadecanone, 2-hydroxy-	543400	
9. Bunium persicum	α-Thujene	97046700	[25]
	α-Pinene	258567152	
	Sabinene	18818	
	β-Pinene	440967	
	Myrcene	31253	
	α-Terpinene	7462	
	P-Cymene	10908223	
	Limonene	22311	
	1, 8-Cineol	46781028	
	γ-Terpinene	7462	
	Terpinolene	11463	
	Linalool	6549	
	Terpinene-4-ol	11230	
	P-Cymen-8-ol	14529	
	Cuminaldehyde	326	
	Thymol	6989	
	α-Methyl-benzene methanol	7409	
	Pinocarvyl acetate	102553	
	Geranyl acetate	1549026	
	Cuminyl acetate	100990	
	γ-Elemene	472424234	
	Caryophyllene	5281515	
	α-Selinene	10856614	
	β-Bisabolene	86597	
	Croweacin	5316141	
	Spathulenol	: 92231	
	Caryophyllene oxide	1742210	
	Dillapiole	10231	
	α-Bisabolol	442343	
10. Caesalpinia decapetala	α-thujene	97046700	[26]
	α-pinene	258567152	
	β-pinene	440967	
	β-myrcene	31253	

	δ-2-carene	16211587	
	α-phellandrene	11142	
	δ-3-carene	26049	
	p-cymene	7463	
	limonene	22311	
	(*Z*)-β-ocimene	5320250	
	phenyl acetaldehyde	998	
	(*E*)-β-ocimene	5281553	
	(6,7)-epoxymyrcene	122371	
	linalool	6549	
	nonanal	31289	
	trans-mentha-2,8-dien-1-ol	155626	
	(4*E*,6*Z*)-*allo*-Ocimene	5371125	
	trans -limonene oxide	449290	
	verbenol	61126	
	p-mentha-1,5-dien-8-ol	519323	
	cis-linalool oxide	6428573	
	trans-linalool oxide	6432254	
	α-terpineol	17100	
	methyl salicylate	4133	
	verbenone	29025	
	trans-carveol	94221	
	geraniol	637566	
	δ-elemene	6918391	
	α-cubebene	442359	
	α-copaene	12303902	
	geranyl acetate	1549026	
	β-bourbonene	62566	
	β-elemene	6918391	
	β-caryophyllene	1742210	
	β-copaene	21722369	
	trans-muurola-3,5-diene	10632031	
	α-humulene	5281520	
	aromadendrene	91354	
	cis-Muurola-4(14),5-diene	162912993	
	germacrene D	5317570	
	bicyclogermacrene	13894537	
	α-muurolene	12306047	
	(E,E)-α-farnesene	5281516	
	γ-cadinene	6432404	
	δ-cadinene	441005	
	trans-cadina-1,4-diene	6430869	
	α-cadinene	12306048	
	(*E*)-nerolidol	5284507	
	spathulenol	92231	
	caryophyllene oxide	1742210	

	viridiflorol	11996452	
	Salvial-4(14)-en-1-one	42608172	
	khusimone	6428327	
	humulene epoxide	5352470	
	1,10-di-*epi*-cubenol	12304224	
	junenol	6452077	
	1-*epi*-cubenol	519857	
	epi-α-muurolol	3084331	
	α-muurolol	3084331	
	α-cadinol	12302222	
	14-hydroxy-9-*epi*-β-caryophyllene	2300118	
	eudesma-4(15),7-dien-1-ole	6429131	
	(2*E*, 6*Z*)-farnesol	1549109	
	14-hydroxy-δ-cadinene	14845382	
	methyl palmitate	8181	
	palmitic acid	985	
	sandaracopimarinal	620819	
	tricosane	12534	
	pentacosane	12406	
	heptacosane	11636	
	nonacosane	12409	
	Î±-pinene	91508	
	Î²-myrcene	31253	
	Î±-phellandrene	11142	
	limonene	22311	
	(Z)-Î²-ocimene	5320250	
	(E)-Î²-ocimene	5281553	
	linalol	6549	
	nonanal	31289	
	Î±-terpineol	442501	
	geraniol	637566	
	Î²-caryophyllene	1742210	
	Î±-humUlene	5281520	
	(E)-nerolidol	5284507	
	Andrographolide	5318517	
	Quercetin	5280343	[27]
	β-Sitosterol	222284	
	Bergenin	66065	
	Rutin	5280805	
	Emodin	3220	
	Betulin	72326	
	StigmasTerol	5280794	
	Baicalein	5281605	
	Polydatin	5281718	
	Salicin	439503	

	Apigenin	5280443	
	Epicatechin	72276	
	Cinnamic acid	444539	
11. Calendula officinalis	Butanone	6569	[28]
	Pentan-2-on	7895	
	a-Pinene	258567152	
	Alpha-Fenchene	28930	
	Camphene	6616	
	/I-Pinene	91508	
	Sabinene	18818	
	A3-Carene	26049	
	a-Phellandrene	7460	
	Myrcene	31253	
	a-Terpinene	7462	
	Limonene	22311	
	1,8-Cineole	2758	
	Beta-Phellandrene	11142	
	cis-b-Ocimene	5320250	
	y-Terpinene	7461	
	trans-b-Ocimene	5281553	
	p-Cymene	7463	
	Terpinolene	11463	
	Menthone	26447	
	a-Copaen	12303902	
	b-Ylangene	519779	
	Camphor	2537	
	a-Gurjunene	521243	
	b-Cubebene	93081	
	Bornyl acetate	93009	
	Aromadendrene	91354	
	trans-Caryophyllene	5281515	
	Terpinene-4-ol	11230	
	y-Cadinene isomers	92313	
	a-Humulene	5281520	
	y- Amorphene	12313019	
	b-Farnesene	5281517	
	y-Muurolene	12313020	
	a-Patchoulene	521710	
	b-Cubebene	93081	
	y-Cadinene	15094	
	y-Patchoulene	12313020	
	a-Muurolene	12306047	
	y,-Cadinene	15094	
	y-Patchoulene	12313020	
	a-Muurolene	12306047	

	y2-Cadinene	92313	
	6 -Cadi n e n e	441005	
	Cubebene	518814	
	a-Cadinene	12306048	
	Calamenene	6429077	
	Calacorene	14038842	
	Nerolidol	5284507	
	Cubenol	11770062	
	Epicubenol	12046149	
	Bisabolol oxide	72720411	
	T-Cadinol	160799	
	a-Muurolol	12306047	
	T-Muurolol	3084331	
	Eudesmane	193299	
	a-Cadinol	10398656	
	Acetyl eugenol	7136	[29]
	Phenol-4-octyl	15730	
	Guaiol	227829	
	Cedrol	65575	
	Octadecane	11635	
	Tetradecanoic acid	11005	
	Nonadecane	12401	
	Eicosane	8222	
	Heneicosane	12403	
	Docosane	12405	
	Tricosane	12534	
	Tetracosane	12592	
	Pentacosane	12406	
	Hexacosane	12407	
	Heptacosane	11636	
	Octacosane	12408	
	Eicosane-7-hexyl	292289	
	Eicosane-9-octyl	280905	
	Canescegenine	12308767	
	Cholest-4-en-3-one-14-methyl	277841	
	Taraxasterol	115250	
	1-Octadecanol	8221	
	1,16-Hexadecanediol	82184	
12. Camellia sinensis	Verbenone	29025	[30]
	Terpinolene	11463	
	Methyl cyclopentene	103212	
	Hexanoic acid 3-hexenyl ester	5352543	
	6-Methyl-5-hepten-2-one	9862	
	Benzene acetaldehyde	998	
	Oxalic acid, 2-phenylethyl propyl ester	6421907	

	Hexanoic acid, hexyl ester	22873	
	Methyl salicylate	4133	
	Ocimene	5281553	
	Alpha-farnesene	5281516	
	Geraniol	637566	
	Butylated Hydroxytoluene	31404	
	Cyclohexadiene	11605	
	Indole	798	
	propionaldehyde	527	[31]
	acetone	180	
	acetone	180	
	ethyl acetate	8857	
	2,5-dimethylfuran	12266	
	2-butanone	6569	
	3-methylbutanal	11552	
	ethanol	702	
	2-ethylfuran	18554	
	pentanal	8063	
	2,3-butanedione	650	
	ethyl acrylate	8821	
	2,3-pentanedione	11747	
	ethyl 2-methylbutanoate	24020	
	ethyl 3-methylbutanoate	7945	
	propyl acetate M	7997	
	propyl acetate D	7997	
	ethyl propanoate	7749	
	α-pinene	258567152	
	1-propanol	1031	
	ethyl butanoate M	7762	
	ethyl butanoate D	7762	
	hexanal	6184	
	2-methylpropanol	6560	
	β-pinene	440967	
	butyl propionate	11529	
	butanol M	263	
	butanol D	263	
	2-methylbutanol	8723	
	pentyl acetate	12348	
	ethyl valerate	10882	
	2-heptanone	8051	
	heptanal	8130	
	limonene M	440917	
	limonene D	440917	
	limonene T	440917	
	3-methylbutanol M	31260	

	o-xylene	7237	
	3-methylbutanol D	31260	
	ethyl caproate	31265	
	2-pentylfuran	19602	
	2-hexanol	12297	
	cyclohexanone	7967	
	beta-ocimene	18756	
	1-pentanol M	6276	
	1-pentanol D	6276	
	(Z)-3-hexenyl acetate	5363388	
	2-methylpyrazine M	7976	
	2-methylpyrazine D	7976	
	2-octanone	8093	
	octanal M	454	
	octanal D	454	
	1-hydroxy-2-propanone	8299	
	(E)-3-hexenol	5281167	
	3-methylpentanol	11508	
	2,5-dimethylpyrazine	31252	
	(E)-2-heptenal	5283316	
	methyl heptenone	9862	
	dimethyl trisulfide	19310	
	1-hexanol M	8103	
	1-hexanol D	8103	
	ethyl lactate	7344	
	(Z)-3-hexenol M	129776539	
	(Z)-3-hexenol D	129776539	
	nonanal	31289	
	2-cyclohexenone	13594	
	2-butoxyethanol	8133	
	(E)-2-octenal	5283324	
	trimethylpyrazine	26808	
	acetic acid	176	
	1-octen-3-ol	18827	
	diallyl disulfide M	16590	
	diallyl disulfide D	16590	
	ethyl octanoate	7799	
	furfural M	7362	
	furfural D	7362	
	(E,E)-2,4-heptadienal M	91752826	
	(E,E)-2,4-heptadienal D	91752826	
	2-acetylfuran	14505	
	(E)-2-nonenal	5283335	
	benzaldehyde M	240	
	benzaldehyde D	240	
	bornyl acetate	93009	

	5-methylfurfural	12097	
	methyl benzoate	7150	
	Hydrazine, 1,1-dimethyl-	5976	[32]
	Benzoyl bromide	12056	
	1-Propanone, 2-bromo-1-phenyl-	16452	
	1,2,4-Triazine-3,5(2H,4H)-dione,6-benzoylthio-	273249	
	Nonanal dimethyl acetal	87813	
	Dodecane, 1,1-dimethoxy-	84559	
	2-Oxiranecarboxylic acid, 3-(2,2imethoxyethyl)-3-methyl-, methylester	569008	
	Cyclotetrasiloxane, octamethyl-	11169	
	6-Azabicyclo[3.2.1]octane	549419	
	Isoxazole, trimethyl-	25375	
	Imidazole-4-carboxamide	152917	
	Benzofuran, 2,3-dihydro-	10329	
	Benzeneethanamine, N-(3-chloropropyl)-.alpha-methyl-	21777	
	N-Methylhexamethylenimine-2-carboxylic acid, ethyl ester	574447	
	Propargylamine, N-trimethylsilyl-	88436902	
	Phenol, 3-fluoro-	9743	
	1,2,3-Benzenetriol	1057	
	Phenol, 4,4′-methylenebis[2,6-dimethyl-	136880922	
	Ethanone, 1-[4-methoxy-3-(4-methylphenoxy)phenyl]-	617872	
	3,5-Dimethyl-1-dimethylphenylsilyloxybenzene	532641	
	Lethane	8196	
	2-Deoxy-D-galactose	102191	
	1,2-Epithio-3-hexanol	545867	
	Caffeine	2519	
	Bicyclo[4.1.0]heptane, 3-methyl—7pentyl-	557165	
	cis,cis-1,9-Dimethylspiro[5.5]undecane	557163	
	9-Octadecen-1-ol, (Z)-	5284499	
	Hexadecanoic acid, ethyl ester	12366	
	Phytol	5280435	
	Linoleic acid ethyl ester	5282184	
	13-Tetradece-11-yn-1-ol	543337	
	Ethyl 9,12,15-octadecatrienoate	5367460	
	9,12,15-Octadecatrienoic acid, ethyl ester, (Z,Z,Z)-	5367460	
	1,3,7,9-Tetramethyluric acid	75324	
	1H-Purine-2,6-dione, 3,7-dihydro-8-(hydroxymethyl)-1,3,7-trimethyl-	235295	
	3-(3-Indolyl)-5-oxo-3-pyrazoline-4	614241	

	Naphthacene-5,12-dione, 6,11-dihydroxy-2,3,8,9-tetramethyl-	631405	
	8-[1-Adamantyl]-1,3-diamino-5,6-dihydrobenzo[f]quinazoline	631428	
	Ethyl 2-(4-nitrophenyl)-7-oxo-7H-1,3,4-thiadiazolo[3,2-a]pyrimidine-5-carboxylate	631401	
	3-Pyrrolidinecarboxamide, 1-methyl-N-[4-(octyloxy)phenyl]-5-oxo-	5305508	
	Estra-1,3,5(10)-trien-17-ol, 2,3,4-trimethoxy-, (17.beta.)-	22296422	
	3-pyrazolidinone, 1-[3-(dodecyloxy)phenyl]-	20778454	
	Naphthacene-5,12-dione, 6,11-dihydroxy-2,3,8,9-tetramethyl-	631405	
	Estra-1,3,5(10)-trien-17-ol, 2,3,4-trimethoxy-, (17.beta.)-	22296422	
	2-Pyrazoline, 5-(4-fluorophenyl)-3-(4-methoxyphenyl)-1-phenyl-	631402	
	1,4-benzenediamine, N1,N4-bis[4-(dimethylamino)phenyl]-	15247162	
	Tetracosanoic acid, methyl ester	75546	
	Methanol, (1-ethyl-2-benzimidazolyl)(2-methoxyphenyl)-	619714	
	Ethyl 2-cyano-trans-3-(2-naphthyl)acrylate	969031	
	2-Hydrazino-8-hydroxy-4-phenylquinoline	619721	
	Oxazole, 2-(3-methoxyphenyl)-5-phenyl-	520919	
	2-(4-Cyanophenyl)-5-dimethylaminomethylenaminopyrimidine	619622	
13. Carica papaya	Acetone	180	[33]
	Ethyl acetate	8857	
	Ethanol	702	
	Pentanal	8063	
	Methyl butanoate	12180	
	Ethyl butanoate	7762	
	Butyl acetate	31272	
	Hexanal	6184	
	Methyl pentanoate	12206	
	Methyl (E)-2-butenoate	638132	
	B-Myrcene Heptanal	31253	
	Methyl hexanoate	7824	
	Ethyl hexanoate	31265	
	(Z)-B-Ocimene	5320250	
	(E)-B-Ocimene	5281553	
	3-Methylbutyl butanoate	7795	
	Methyl (E)-3-hexenoate	5362782	
	Acetoin	179	
	Octanal	454	

	Methyl (E)-2-hexenoate	5364409	
	6-Methyl-5-hepten-2-one	9862	
	1-Hexanol	8103	
	Methyl 2-hydroxybutanoate	520445	
	Methyl octanoate	8091	
	Nonanal	31289	
	Methyl 2-hydroxy-3-methylbutanoated	552631	
	Methyl 2-(methylthio) acetate	4447651	
	Methyl 2,4-hexadienoate	5323650	
	Ethyl benzyl ether	10873	
	(E)-Linalool furanoxide	6431475	
	Methyl (E,E)-2,4-hexadienoate	5323650	
	Furfural	7362	
	1-Heptanol	8129	
	(Z)-Linalool furanoxide	22310	
	Methyl 3-hydroxybutanoate	15146	
	(E,E)-2,4-Heptadienal"	5283321	
	Decanal	8175	
	1-(2-Furanyl)-ethanone	14505	
	Benzaldehyde	240	
	Methyl 2-hydroxy-4-methylpentanoate	62908	
	Linalool	6549	
	1-Octanol	957	
	5-Methylfurfural	12097	
	Methyl decanoate	8050	
	Methyl benzoate Hotrienol	7150	
	Butanoic acid	264	
	Methyl 3-hydroxyhexanoate	519845	
	4-Methyl-4-vinylbutyro-lactone	7921	
	1-Nonanol +	8914	
	Benzyl isocyanate y-Hexalactone	76639	
	Methyl geranate	5365910	
	a-Terpineol	17100	
	Dodecanal	8194	
	(E)-Linalool pyranoxide	6427788	
	Methyl salicylate	4133	
	(Z)-Linalool pyranoxide	6431477	
	8-Hexalactone	13204	
	Ethyl dodecanoate	7800	
	(E)-Geraniol +	637566	
	Dihydropseudoionone	1549778	
	Benzyl butanoate	7650	
	Hexanoic acid	8892	
	Benzyl alcohol	244	
	Epoxylinalool furanoxide, (isomer 1)	132277303	
	Epoxylinalool furanoxide, (isomer 2)	132277303	

	Geranyl butanoate	5355856	
	γ-Octalactone	7704	
	Benzyl nitrile	8794	
	ß-Ionone	638014	
	8-Octalactone	12777	
	2,6-Dimethyl-3,7-octadiene-2,6-diol	5352451	
	1-Dodecanol	8193	
	Octanoic acid	379	
	1-Tridecanol,d	8207	
	Benzyl isothiocyanate	2346	
	Epoxy Linalool pyranoxide	132277303	
	2,6-Dimethyl-1,7-octadiene-3,6-diol	548927	
	2-Ethylhexyl salicylated	8364	
	Decanoic acid	2969	
	Dodecanoic acid	3893	
	Benzyl salicylate	8363	
	2,2,2-trifluoro-acetamide	67717	[34]
	Methyl 9-methyltetradecanoate	554137	
	Hexadecanoic acid methyl ester	8181	
	Cis-10-nonadecenoic acid methyl ester	14178778	
	Trimethyl silanol	66110	
	Dodecamethyl cyclohexasiloxane	10911	
	Glycerin	753	
	Benzyl nitrile	8794	
	Tetradecanoic acid methyl ester	31284	
	Benzoic acid	243	
	Benzeneacetic acid	999	
	Isothiocyanatomethyl benzene	29350	
	Benzeneacetamide	7680	
	Dodecanoic acid	3893	
	Tetradecanoic acid	11005	
	Hexadecanoic acid methyl ester	8181	
	Methyl stearate	8201	
	Cis-13-octadecenoic acid, methyl ester	12541027	
	Hexadecanoic acid	985	
	9(E)-Octadecenoic acid methyl ester	5280590	
	16-methyl-heptadecanoic acid methyl ester	21859	
	9(Z)-Octadecenoic acid methyl ester	5364509	
	9(Z)-Octadecenoic acid	445639	
	10-Octadecenoic acid methyl ester	25642	
	9(Z),12(Z)-Octadecadienoic acid methyl ester	5284421	
	Cis-6-Octadecenoic acid	5281125	
	2-hydroxy-1-(hydroxymethyl)-hexadecanoic acid ethyl ester	129853056	

	Cis-11-octadecenoic acid, methyl ester	5364505	
	1,1,3,3,5,5-hexamethyl trisiloxane	6327152	
	Benzeneacetamide	7680	
	Trimethylsilyl 2-(2-(2-butoxyethoxy)ethoxy)acetate	31288	
	1-Monooleoylglycerol methyl ether	5283468	
	9(E)-Octadecenoic acid	445641	
	2-hydroxy-1-(hydroxymethyl)-9(Z)-Octadecenoic acid ethyl ester	5365676	
	2,3-Dihydroxypropyl elaidate	5364833	
	Squalene	638072	
	Campesterol	173183	
	Stigmasterol	5280794	
	B-Sitosterol	222284	
	38-9,19-Cyclolanost-24-en-3-ol	129857152	
	Stigmast-4-en-3-one	5484202	
	5a-Stigmastane-3,6-dione	13992092	
	4-hydroxybenzaldehyde	126	
	Cis-9-Azabicyclo Nonane	144593	
	N,O-Bis(trimethylsilyl)trifluoroacetamide	24849493	
	Benzeneacetamide	7680	
	Ethanamine	6341	
	Galactose methoxy- amine	6421155	
	4-hydroxyben- zenemethanol	125	
	md fatty acid methyl este	14259	
	Nonanoic acid	8158	
	Dodecanoic acid"	3893	
	Octanedioic acid	10457	
	Tetradecanoic acid”	11005	
	Hexadecanoic acid methyl ester	8181	
	16-methyl-heptade-canoic acid methyl ester	110444	
	Trans-9-octadecenoic acid methyl ester"	5280590	
	14-methyl-heptade-canoic acid methyl ester	520159	
	Pentadecanoic acid	13849	
	Cis-9-hexadecenoic acid	445638	
	Hexadecanoic acid	985	
	Cis-9-octadecenoic acid	445639	
	Cis-6-octadecenoic acid	5281125	
	Cis-9, cis-12-octa-decadienoic acid methyl ester	5284421	
	Cis-13-octadecenoic acid methyl ester	12541027	
	Cis-10-heptadecenoic acid	5312435	
	Heptadecanoic acid	10465	
	Trans-13-octadece- noic acid	6161490	
	Linoleic acid	5280450	
	Trans-11-octadece- noic acid	5281127	

	Octadecanoic acid	5281	
	Cis-13-octadecenoicacid	5312441	
	Cis-9-octadecenoic acid	445639	
	Nonadecanoic acid	12591	
	11-eicosenoic acid	5282768	
	Eicosanoic acid	10467	
	Heneicosanoic acid	16898	
	Cis-vaccenic acid"	5282761	
	2-hydroxy-1-hexa-	11953951	
	decanoic acid ethyl ester"	8048	
	Octadec-9-enoic acid	965	
	Oleic acid eicosyl ester	6436542	
	9-hexadecenoic acid	5282745	
	Cis-13-docosenoic acid	5281116	
	Docosanoic acid	8215	
	2-hydroxy-1-cis- 9-octadecenoic acid ethyl ester"	5365676	
	Octadecanoic acid 2,3-dihydroxypropyl ester	24699	
	Cis-15-tetracosenoic acid	5281120	
	Tetracosanoic acid	11197	
	n-propyl 9-octadecenoate	5356108	
	Pentacosanoic acid	10468	
	Hexacosanoic acid	10469	
	Cis-13-octadecenal	5364497	
	Cis-9, cis-17-octa-	5364492	
	decadienal Cis-9-octadecenal	5364492	
	Glycerol"	753	
	Propylene glycol	1030	
	Bromazepam	2441	
	2-ethylacridine	610161	
	Isothiocyanatometh- ylbenzene9	2346	
	2-hydroxy-cyclopen- tadecanone	543400	
	a-cyperone	6452086	
	2-phenylacetonitrile"	8794	
	Oleanitrile	6420241	
	Glycolic acid	757	
	Benzoic acid"	243	
	Phosphoric acid	1004	
	Nicotinic acid	938	
	Benzeneacetic acid"	999	
	Succinic acid	1110	
	Methylsuccinic acid	10349	
	Mandelic acid	1292	
	Pyroglutamic acid	7405	
	Pyrogallic acid	1057	
	3-hydroxybenzoic acid,	7420	

	Phenyllactic acid	1303	
	4-hydroxybenzoic acid	135	
	1,4-benzenedicarbox- ylic acid	7489	
	Azelaic acid	2266	
	Phenol 2,4-bis(1,1- dimethylethyl)-phenol	528937	
	2,2'-methylenebis[6-(1,1-dimethylethyl)-4-ethyl-]phenol	6928	
	Octamethyltrisiloxane	24705	
	Sitostenone	5484202	
	5α-stigmastane-3,6- dione	13992092	
	Campesterol	173183	
	Stigmasterol	5280794	
	β-Sitosterol	222284	
	Cycloartenol	92110	
	d -(+)-Arabitol	529415	
	β- d -(+)-xylopyranose	135191	
	α-d-mannopyranose	18950	
	Sucrose	5988	
	d -(+)-trehalose	7427	
	Squalene	638072	
14. Capsicum frutescens	Heptyl isopentanoate	91850	[35]
	Hexyl isohexanoate	530286	
	Benzyl butanoate	7650	
	Heptyl pentanoate	79544	
	Hexyl hexanoate	22873	
	Benzyl isopentanoate	7651	
	(E)-2-Hexenyl hexanoate	5352973	
	Benzyl pentanoate	82584	
	Benzyl hexanoate	23367	
	Hexyl benzoate	23235	
	(E)-2-Hexenyl benzoate	5352456	
	Benzyl benzoate	2345	
	Hexyl decanoate	82635	
	Alcohols Isobutanol	6560	
	1-Penten-3-ol	12020	
	Isopentanol	31260	
	1-Pentanol	6276	
	(Z)-2-Pentenol	5364919	
	Isohexanol	12296	
	(Z)-3-Hexenol	5281167	
	(E)-2-Hexenol	5318042	
	1-Hexanol	8103	
	1-Octen-3-ol	18827	
	Ethyl-2-hexanol	13008217	
	(Z)-2-Octen-1-ol	5364959	
	1-Octanol	957	

	2-Decanol	14254	
	3,3-Dimethylcyclohexanol	79105	
	Pentadecanol	12397	
	Hexadecanol	2682	
	Aldehydes and ketones	405237873	
	Pentanal	8063	
	(E)-2-Pentenal	5364752	
	2,3-Hexanedione	19707	
	3-Methyl-2-butenal	61020	
	Hexanal	6184	
	(Z)-2-Hexenal	6428782	
	2-Heptanone	8051	
	Heptanal	8130	
	(E,E)-2,4-Hexadienal	637564	
	(E)-2-Heptenal	5283316	
	Benzaldehyde	240	
	2,3-Octanedione	11449	
	(E,E)-2,4-Heptadienal	5283321	
	(E)-2-Octenal	5283324	
	(E)-2-Nonenal	5283335	
	(Z)-2-Decenal	5354834	
	(E,E)-2,4-Decadienal	5283349	
	Tetradecanal	31291	
	Pentadecanal	17697	
	Hexadecanal	984	
	Terpenes	5281515	
	Tricyclene	79035	
	a-Pinene	258567152	
	Ethyl acetate	8857	
	Isopentyl acetate	31276	
	Butyl isobutanoate	7353	
	2-Methylbutyl isobutanoate	97883	
	Isobutyl isopentanoate	11514	
	Isopentyl isobutanoate	519786	
	Butyl isopentanoate	7981	
	Pentyl isobutanoate	75554	
	Isopentyl butanoate	7795	
	Pentyl butanoate	10890	
	Methyl benzoate	7150	
	Isopentyl 2-methylbutanoate	520326	
	Isopentyl isopentanoate	12613	
	Pentyl isopentanoate	95978	
	Pentyl 2-methylbutanoate	107059	
	Isobutyl hexanoate	7775	
	Pentyl pentanoate	62433	
	Hexyl isobutanoate	16872	

	Isoprenyl pentanoate	6429322	
	Methyl 2-methyloctanoate	519895	
	(Z)-3-Hexenyl butanoate	5352438	
	Butyl hexanoate	12294	
	Methyl salicylate	4133	
	(Z)-3-Hexenyl	5367681	
	2-methylbutanoate	22253297	
	Hexyl 2-methylbutanoate	24838	
	(Z)-3-Hexenyl isopentanoate	5367681	
	Hexyl isopentanoate	61455	
	Isopentyl hexanoate	16617	
	(Z)-2-Hexenyl isopentanoate	21159478	
	Pentyl isohexanoate	10886	
	Isohexyl tiglate	637523	
	Camphene	6616	
	Sabinene	18818	
	B-Pinene	440967	
	Myrcene	31253	
	Limonene	22311	
	(Z)-ß-Ocimene	5320250	
	(E)-ß-Ocimene	5281553	
	Terpinolene	11463	
	(Z)-Linalool oxide (furanoid)	22310	
	Linalool	6549	
	a-Terpineol	17100	
	(Z)-a-Bergamotene	12300073	
	B-Cedrene	11106485	
	Dihydro-ß-ionone	519382	
	(Z)-a-Bergamotene	12300073	
	a-Himachalene	11830551	
	a-Humulene	5281520	
	(E)-ß-Farnesene	5281516	
	allo-Aromadendrene	42608158	
	Himachalene	11586487	
	(E)-beta-ionone	638014	
	Bicyclogermacrene	13894537	
	B-Himachalene	11586487	
	Cadinene	3032853	
	cis-Calamenene	6429077	
	cis-Cadina-1(2),4-diene	91753200	
	(E)-Nerolidol	5284507	
	a-Cadinol	10398656	
	Hexanoic acid	8892	
	Nonanoic acid	8158	
	Decanoic acid	2969	
	Tetradecanoic acid	11005	

	Pentadecanoic acid	13849	
	(Z)-9-Hexadecenoic acid	445638	
	Hexadecanoic acid	985	
	Acetal	7765	
	1-Methyl-1-pyrrole	7304	
	Methional	18635	
	1-Butoxy-2-propanol	21210	
	2-Pentylfuran	19602	
	o-Guaiacol	460	
	2-Isobutyl-3-methoxypyrazine	32594	
	Tetradecane	12389	
	Pentadecane	12391	
	Hexadecane	11006	
	Heptadecane	12398	
	Nonadecane	12401	
15. Cassia alata	2-Hexenal	5281168	[36]
	2-Hexen-1-ol,	5318042	
	(E)- 1-Hexanol	59390510	
	2-Heptanol	10976	
	Anisole	7519	
	Benzaldehyde	240	
	1-Octen-3-ol	18827	
	Phenol	996	
	5-Hepten-2-one, 6-methyl-	9862	
	3-Octanol	11527	
	5-Hepten-2-ol, 6-methyl-	20745	
	Hexanoic acid, ethyl ester	31265	
	Octanal	454	
	Anisole, o-methyl-	33637	
	Acetic acid, hexyl ester	8908	
	4-Hepten-1-ol, 6-methyl-	13494536	
	3-Ethyl-4-methylpentan-1-ol	549664	
	p-Cymene	7463	
	Limonene	22311	
	Eucalyptol	2758	
	Benzyl alcohol	244	
	Benzeneacetaldehyde	998	
	Ether, benzyl ethyl	10873	
	Fenchone	82229	
	2-Nonanone	13187	
	Ethyl (4E)-4-heptenoate	5362816	
	Linalool	6549	
	Nonanal	31289	
	B-Thujone	91456	
	Phenylethyl alcohol	6054	

	3-Thujanone	11027	
	Veratrol	7043	
	2,6-Nonadienal, (E,Z)-	11196	
	2-Nonenal, (E)-	5283335	
	endo-Borneol	6552009	
	Hexanoic acid, butyl ester	12294	
	benzyl alcohol	244	[37]
	phenyl acetaldehyde	988	
	trans-linalool oxidet	6432254	
	cis-linalool oxidet	102611	
	linalool	6549	
	camphor	2537	
	(Z)-3-hexenyl isobutyrate	5352539	
	linalool oxide (pyran)*	6431476	
	borneol	6552009	
	p-cymen-8-ol	14529	
	(Z)-3-hexenyl butanoate	5352438	
	methyl salicylate	4133	
	a-terpineol	17100	
	1-dodecene	8183	
	nerol	643820	
	hexyl-2-methylbutanoate	24838	
	(Z)-3-hexenyl isovalerate	5367681	
	geraniol	637566	
	linalyl acetate	8294	
	(Z)-3-hexenyl valerate	5367682	
	geranyl formate	5282109	
	hexyl tiglate	637523	
	(Z)-3-hexenyl isohexanoate	5352539	
	Neophytadiene	10446	[38]
	6,10,14-trimethyl-2- pentadecanone	10408	
	Methyl palmitate	8181	
	Palmitic acid	5280531	
	Ethyl hexadecanoate	12366	
	Methyl linoleate	5284421	
	Methyl 11-octadecenoate	5364432	
	Methyl stearate	8201	
	Linoleic acid	5280450	
	(E)-9-Octadecenoic acid	637517	
	Stearic acid	5281	
	4,8,12,16-Tetramethylheptadecan-4-olide	567149	
	tert-Butyl 8-Methyl-10-azabicyclo[4.3.1] deca-3,7- diene-10-carboxylate	11644492	
	Erucylamide	5365371	
	6-fluoro-4,6-cholestadien-3ß-ol	14795191	

	n-Eicosane	8222	
	Vitamin E	14985	
	1,19-Eicosadiene	519006	
	Stigmasterol	5280794	
16. Cassia fistula	3-Hydroxypyridine	92043179	[39]
	Betaine	247	
	Nicotinic acid	938	
	4-Piperidone	33721	
	8-Hydroxyquinoline	1923	
	Adipic acid	196	
	Vanillin	1183	
	Quinine	3034034	
	B-Asarone	5281758	
	(E)-Ferulic acid	13916048	
	Butein	5281222	
	(+)-Naringenin	667495	
	7-Ethoxycoumarin	35703	
	Apigenin	5280443	
	4-Methoxycinnamic acid	699414	
	(E)-4-Methoxycinnamic acid	699414	
	Kaempferol	5280863	
	Aloe-emodin	10207	
	(+)-[6]-Gingerol	442793	
	(+)-ar-Turmerone	160512	
	6-Gingerol	442793	
	10-Gingerol	168115	
	Caffeic acid	689043	
	4-Hydroxycoumarin	54682930	
	(E)-parinaric acid	5282838	
	(+/-)-Methoprene	1711973	
	Oleanolic acid	10494	
	Lup-20(29)-en-28-al, 3beta-hydroxy-	99615	
	Asiatic acid	119034	
	Lupa-12,20(29)-dien-3-one	22216693	
	Erucamide	5365371	
	Betulin	72326	
	(22E)-Stigmasta-5,22-dien3-ol	6432745	
	a-terpineol	17100	[40]
	methyl salicylate	4133	
	nerol	643820	
	tridecane	12388	
	eugenol	3314	
	tetradecene*	14260	
	tetradecane	14260	
	methyl eugenol	7127	

	(E)-α-ionone	638014	
	neryl acetone	1713001	
	(Z)-B-farnesene	5317319	
	cabreuva oxide A	91700484	
	cabreuva oxide B	15677814	
	(E)-ß-ionone	638014	
	pentadecane	12391	
	2-tridecanone	11622	
	B-bisabolene	10104370	
	elemicin	10248	
	(E)-nerolidol	5284507	
	isoelemicin	5318557	
	1-hexadecene	12395	
	hexadecane	11006	
	heptadecane	12398	
	2-hexadecanone	29251	
	1-octadecene	8217	
	octadecane	11635	
	hexahydrofarnesylacetone	10408	
	benzyl salicylate	8363	
	nonadecane	12401	
	2-heptadecanone	18027	
	(E,E)-farnesylacetone	1711945	
	methyl hexadecanoate	8181	
	eicosene*	18936	
	hexadecanoic acid	985	
	eicosane	8222	
	methyl linoleate	5284421	
	methyl linolenate	5319706	
	(E)-phytol	6442409	
	docosene*	74138	
	docosane	12405	
	tricosane	12534	
	pentacosane	12406	
	heptacosane	11636	
	**nonacosane**	12409	
	Decane	15600	[41]
	Benzyl alcohol	244	
	2-Pyrrolidinone, 1-methyl-	13387	
	Benzene, 1,2-diethyl-	8657	
	Aniline,	6115	
	N-methyl-	3034287	
	Benzene, 4-ethyl-1,2-dimethyl-	13629	
	Benzene, 1,2,4,5-tetramethyl-	7269	
	1-Undecanol	8184	

	Naphthalene	931	
	1-Hexadecanol	2682	
	Hexadecane	11006	
	Pentadecane	12391	
	Phenylethanolamine	1000	
	5-Octadecene,	5364598	
	(E)- Heneicosane	12403	
	3-Eicosene, (E)-	5365051	
	Heptacosane	11636	
	Ethylene glycol, O,O-di(pivaloyl)-	88442	
	Hexadecanoic acid, 15-methyl-, methyl ester	522345	
	Trifluoroacetoxy hexadecane	522035	
	Cyclooctene, 1,2-dimethyl-	5362808	
	Heptadecyl trifluoroacetate	14574252	
	Heptadecane	12398	
	Hexacosane	72801	
	Octadecyl trifluoroacetate	522719	
	Tetratetracontane	23494	
	Benzenebutanamine	83242	
	Butanoic acid, methyl ester	12180	
	2-Ethylformanilide	243583	
	1-Docosene	74138	
	1-Hexene-3,5-dione	182705	
17. Catharanthus roseus	glycolic acid,	71771	[42]
	threonic acid,	151152	
	threitol,	169019	
	anhydroglucitol,	445555	
	digalacturonic acid,	439694	
	galactonic acid,	128869	
	gentiobiose,	441422	
	octanal,	454	
	sorbitol,	5780	
	itaconic acid,	811	
	cellobiose,	10712	
	Maleamate,	5460391	
	methyl- galactopyranoside,	76935	
	glucopyranoside,	5988	
	glycocyamine,	763	
	aminooxyacetic acid,	286	
	loganin,	87691	
	Threonine,	6288	
	Threonine,	6288	
	glyceric acid,	752	
	dihydroxybenzoic acid,	3469	
	tartaric acid,	444305	

	quinic acid,	6508	
	glucoheptonic acid,	25588	
	trehalose,	7427	
	Hydroxybutyrate,	3037032	
	Lactitol,	157355	
	levoglucosan,	91696777	
	gallic acid,	370	
	ethanolamine,	700	
	lyxose,	439240	
	aminobutyric acid,	119	
	succinic acid,	1110	
	salicin,	439503	
	Maltotriose,	192826	
	raffinose,	2724100	
	saccharic acid,	33037	
	isoleucine,	6306	
	glucose-1-phosphate,	65533	
	hydroxypropionic acid,	68152	
	methionine,	876	
	aminoisobutyric acid,	6119	
	serine,	5951	
	ornithine,	6262	
	dodecanol,	8193	
	methyl phosphate,	3039	
	erythrose,	94176	
	methoxytryptamine,	1833	
	mono phthalate,	119096	
	fucose,	17106	
	caffeic acid,	689043	
	xylitol,	6912	
	nicotinoylglycine,	101868037	
	malic acid,	525	
	citramalic acid,	439766	
	glycolic acid,	71771	
	pyruvic acid,	1060	
	allothreonine,	99289	
	aspartic acid,	424	
	oxoproline,	7405	
	galactinol,	11727586	
	chlorogenic acid,	1794427	
	ribose,	10975657	
	myo-inositol,	892	
	tagatose,	439312	
	fructose,	2723872	
	talose,	441035	
	mannitol,	6251	

	glucose,	5793	
	Anhydrogalactose,	16069996	
	phosphate,	1061	
	fumaric acid,	444972	
	gluconic lactone,	7027	
	dimethylsuccinic acid,	11701	
	glucose-6-phosphate,	439958	
	valine,	6287	
	tyrosine,	6057	
	Amino-1-phenylethanol,	1000	
	glutamic acid,	33032	
	maleic acid,	444266	
	glutamine,	5961	
	isocitric acid,	1198	
	alpha-ketoglutaric acid,	51	
	acetyl-glutamic acid,	70914	
	galactose,	6036	
	phytol,	5280435	
	norleucine,	21236	
	oxalic acid,	971	
	mannosylglycerate,	443262	
	glutaconic acid,	5280498	
	alanine,	5950	
	lysine,	5962	
	lactic acid,	612	
	methyl-amino-1,2-propanediol,	45202951	
	glycerol,	753	
	proline,	145742	
	hydroxynorvaline	65097	
	Hexadecanoic acid, methyl ester	8181	[43]
	Pentadecanoic acid	13849	
	9,12,15-Octadecatrienoic acid,	5280934	
	Octadecane,	11635	
	3-ethyl-5-(2-ethylbutyl)-	92285	
	9,12,15-Octadecatrienoic acid, methyl ester	5367462	
	L-Glucose	10954115	
	Sucrose;	5988	
	D-Glucose-6-O	219994	
	D-fructose	2723872	
	D-allose	439507	
	Muco-Inositol	892	
	a-D-Glucopyranoside,	7427	
	alpha-D-glucopyranosyl-(1->3)-alpha-D-fructofuranosyl	16058661	
	Myo-Inositol, 4-C-methyl-	244581	

	2,20-Cycloaspidospermidine-3-carboxylic acid Vindolinine	11210355	
	Ascaridole epoxide	584498	
	Phytol	5280435	
	3'5'-Dimethoxyacetophenone	95997	
	4H-Pyran-4-one/Maltol	8369	
	1,2,3-Propanetriol, 1-acetate/acetin	33510	
	5Hydroxymethylfurfural	237332	
	Deoxyspergualin	91272	
	Desulphosinigrin	9601716	
	Catechol/Resorcinol	19354071	
	Benzofuran, 2,3-dihydro	10329	
	1,2,3,5-Cyclohexanetetrol+	548226	
	Condyfolan, 14,19-didehydro-12-methoxy-, (14E)-	5372241	
	(1R,3R,4R,5R)-(-)-Quinic acid	6508	
	Phthalic acid, di(oct-3-yl) ester	15764573	
	3-Methyl-1-butanol(a)	31260	[44]
	2-Methyl-1-butanol(*)	8723	
	1-Pentanol	6276	
	cis-2-Penten-1-ol(*)	5364919	
	cis-3-Hexen-1-ol(4)	5281167	
	trans-3-Hexen-1-ol(4)	5284503	
	1-Hexanol(*)	8103	
	Benzyl alcohol(a)	244	
	1-Phenylethanol()	7409	
	trans-2-Octen-1-ol(a)	5318599	
	2-Nonen-1-ol(a)	61896	
	6-Methyl-2-hepten-2-ol(4)	11240490	
	2-Phenylethanol(*)	6054	
	trans-2-Decen-1-ol(a)	5364942	
	1-Octen-3-one(4)	61346	
	6-Methyl-5-hepten-2-one	9862	
	**cis-Jasmone(4)**	1549018	
	gamma-Decalactone	12813	
	3,6-Dimethyl-5-octen-2-one(4)	5363295	
	Hexahydrofarnesylacetone	10408	
	3-Methylbutanal(4)	11552	
	Pentanal()	8063	
	2-Methylbutanal()	7284	
	n-Hexanal(a)	9603563	
	Methional(4)	18635	
	Benzaldehyde	240	
	Octanal	454	
	Phenylacetaldehyde)	998	
	trans-2-Octenal	5283324	

	cis-2-Nonenal	5354833	
	cis-2-Decenal	5354834	
	p-Hydroxy-benzaldehyde	126	
	Ethyl hexanoate	31265	
	Isopropyllaurate	135400705	
	Methyljasmonate(a)	5281929	
	Isopropyl myristate()	8042	
	Palmitate acid methyl ester	8181	
	Palmitic acid ethyl ester(4)	12366	
	Isopropyl palmitate()	8907	
	Methyl linolenate(*)	5319706	
	Linolenic ethyl ester(4)	9883013	
	B-Pinene(*)	440967	
	Limonene	22311	
	**1,8-Cineol**	2758	
	Linalool	6549	
	Limonene oxide	91496	
	Camphora	159055	
	cis-Menthone(*)	6432469	
	Menthol	1254	
	(+)-a-Terpineol	17100	
	(+)-Carvone(a)	16724	
	1-(-)-Menthol(4)	131738477	
	trans-Geranylacetone	1549778	
	a-Bisabolol(4)	1549992	
	Manoyl oxide(a)	6432025	
	epi-Manoyl oxide	18529657	
	Manool(a)	3034394	
	trans-Phytol (1)	5280435	
	cis, trans-2,6-Nonadienal(*)	12106224	
	Safranal	61041	
	trans, cis-2,4-Decadienal	5283349	
	B-Cyclocitral	9895	
	B-Homocyclocitral	61124	
	Citral	638011	
	trans, trans-2,4-Decadienal()	5283349	
	a-lonone	5363685	
	Dihydro-B-ionone(*)	519382	
	B-lonone	5282108	
	2,3-Epoxy-a-ionone	129826809	
	Dihydroactinidiolide	6432173	
	2-Methyl-pyridine(*)	7975	
	2,6-Dimethylpyridine	7937	
	2,4-Dimethylpyridine(*)	7936	
	3-Ethylpyridine	10823	
	3-Methoxy-2,5- dimethylpyrazine(*)	586728	

	2-Isopropyl-3- methoxypyrazine(4)	33166	
	2-Isobutyl-3- methoxypyrazine(*)	32594	
	1,3-Benzothiazole(*)	39040	
	1H-indole(a)	798	
	Methyl anthranilate(*)	8635	
	Isophytol	10453	
	p-Hydoxycinnamic acid(a)	637542	
	Palmitic acid	985	
	5-Methyl-resorcinol(4)	10436	
	2-Methoxy-4-vinylphenol(a)	332	
	Eugenol	3314	
	3-Ethyl-5- methylpyridine(*)	19901	
	3,7,11,15- Tetramethyl-2- hexadecen-1- ol(4)	5366244	
	Tetradecane	12389	[45]
	Pentadecane	12391	
	Hexadecane	11006	
	Heptadecane	12398	
	Octadecane	11635	
	1-[(E)-1-Hexenyl]-cyclohexanol	5352482	
	Heptanal	8130	
	Octanal	454	
	(E,E)-2,4-Heptadienal	5283321	
	(E)-2-Octenal	5283324	
	Nonanal	31289	
	(E)-2-Nonenal	5283335	
	Decanal	8175	
	(E)-2-Decenal	5283345	
	(E,Z)-2,4-Decadienal	6427087	
	(E,E)-2,4-Decadienal	5283349	
	(E)-2-Undecenal	5283356	
	Pentadecanal	17697	
	6-Methyl-5-hepten-2-one	9862	
	Heptenone	14243	
	2,2,6-Trimethylcyclohexanone	17000	
	3-Octen-2-one	5363229	
	(E,Z)-3,5-octadien-2-one	6427789	
	(E,E)-Octa-3,5-dien-2-one	5352876	
	6-Methyl-3,5-heptadien-2-one	5370101	
	Pentadecane-2-one	495487	
	Caproic acid	8892	
	Methyl heptanoate	7826	
	Methyl caprylate	8091	
	Caprylic acid	379	
	Nonanoic acid	8158	
	**Capric acid**	2969	

	Methyl undecanoate	15607	
	Methyl laurate	8139	
	**Lauric acid**	3893	
	Tridecanoic acid	12530	
	**Methyl myristate**	31284	
	Myristic acid	11005	
	**Methyl pentadecanoate**	23518	
	Pentadecanoic acid	13849	
	Methyl 9-tridecenoate	1778877	
	Methyl palmitate	8181	
	a-Isophorone	6544	
	(Z)-Linalool oxide	22310	
	(E)-Linalool oxide	6432254	
	Linalool	6549	
	Safranal	61041	
	B-Cyclocitral	9895	
	Neral	643779	
	**Geranial**	638011	
	Beta-Pulegone	6988	
	Geranylacetone	1549778	
	Germacrene D	5317570	
	Dihydroactinidiolide	27209	
	(E,E)-Farnesol	445070	
	(E,E)-Farnesyl acetate	638500	
	Hexahydrofarnesylacetone	10408	
	Farnesylacetone	1711945	
	Edulane II	91747161	
	Theaspirane B	61953	
	Theaspirane A	6431226	
	(E)-Beta-Damascenone	5366074	
	(E)-alpha-ionone	5282108	
	(E)-Beta-ionone-5,6-epoxide	6430868	
	(E)-Beta-ionone	638014	
	Megastigmatrienone	6437599	
	(E,E)-Pseudoionone	1757003	
	Eugenol	3314	
	(E)-Anethole	637563	
	Benzaldehyde	240	
	4-Methylbenzaldehyde	7725	
	Ethylbenzaldehyde	123406	
	Methyl salicylate	4133	
	2,6,6-Trimethylcyclohexene-1-carboxylic acid	3013889	
	1-acetaldehyde	61124	
18. Cecropia obtusifolia	isoorientin-2”-O-xyloside	44257921	[46]
	isoorientin-4”-O-xyloside	44257975	

	isoorientin-2”-O-rhamnoside	50993776	
	isovitexin-2”-O-xyloside	44257697	
	isovitexin-2”-O-glucoside	9916590	
	isovitexin-2”-O- rhamnoside	23844078	
19. Cichorium intybus	Undec-3-en-2-ol	71387335	[47]
	2-Nitrohexane	536519	
	2,2-Dimethyl-pentan-1-ol	16911	
	5-Methyl-2- furancarboxaldehyde	12097	
	3-Hexen-2-one	5367744	
	5-Hexen-2-one	7989	
	Maltol	8369	
	5-Hydroxymethylfurfural	237332	
	2-Acetyl-resorcinol	69687	
	5-Acetoxymethyl-2- furaldehyde	66349	
	Hexadecanoic acid	985	
	Ethyl hexadecanoate	12366	
	Methyl linoleate	5284421	
	Ambrosia	16131132	
	Ethyl octadec-9,12- dienoate	5365672	
	5-formyl-2-furyl methyl acetate	66349	
	Squalene	638072	
	2-[1- (Hydroxymethyl)cyclohexyl] ethanol	68274233	
	Stigmasterol	5280794	
	δ-Friedoolean-14-en-3- one	92785	
	Stigmast-5-en-3-ol	20831071	
	β-amyrin	73145	
	α-amyrin	73170	
	Stigmast-4-en-3-one	5484202	
	Lup-20(29)-en-3-yl acetate	323074	
	Clerodol	259846	
	Lupan-3-yl- acetate	604899	
	Lupeol acetate	92157	
	n-Hexanol	8103	[48]
	trans-Linalool oxide	6432254	
	Linalool	6549	
	6-Methyl-3,5-heptadien-2-one	5370101	
	Camphor	2537	
	Borneol	64685	
	4-Terpineol	11230	
	1-Methyladamantane	136607	
	Naphthalene	931	
	α-Terpineol	442501	
	Cuminic aldehyde	326	
	Carvone	7439	
	Cinnamic aldehyde	637511	

	Thymol	6989	
	Carvacrol	10364	
	[3.3.3] Propellane	143555	
	Eugenol	3314	
	Piperitenone oxide	442497	
	p-Methoxy cinnamic aldehyde	41294	
	Ethyl phthalate	6781	
	β-Caryophyllene	5281515	[49]
	trans-α-Bergamotene	6429302	
	Germacrene D	5317570	
	Pentadecane	12391	
	Spathulenol	92231	
	Caryophyllene oxide	1742210	
	Tetradecanol	8209	
	Heptadecane	12398	
	Octadecane	11635	
	6,10,14-Trimethylpentadecan-2-one	10408	
	Hexadecanol	2682	
	Nonadecane	12401	
	Hexadecanoic acid	985	
	Eicosane	8222	
20. Cinnamomum zeylanicum	Ascorbic acid	54670067	
	Epigallocatechin	72277	[50]
	Chlorogenic acid	1794427	
	Fumaric acid	444972	
	Verbascoside	5281800	
	Orientin	5281675	
	Caffeic acid	689043	
	Luteolin-7-rutinoside	10461109	
	Luteolin 7-glucoside	5280637	
	Rutin	5280805	
	Rosmarinic acid	5281792	
	Hyperoside	5281643	
	Apigenin 7-glucoside	5385553	
	Ellagic acid	5281855	
	Quercitrin	5280459	
	Quercetin	5280343	
	Herniarin	10748	
	Salicylic acid	338	
	Naringenin	439246	
	Luteolin	5280445	
	Apigenin	5280443	
	Hispidulin	5281628	
	Isosakuranetin	160481	

	Penduletin	5320462	
	CAPE	5281787	
	Chrysin	5281607	
	Quillaic acid	101810	
	Caryophyllene oxide	1742210	
	heptanal*	8130	[51]
	alpha-pinene	258567152	
	nonanal	31289	
	linalool	6549	
	alpha-copaene	12303902	
	alpha-bergamotene	86608	
	trans-cinnamyl acetate	5282110	
	aromadendrene	91354	
	alpha-cadinene	12306048	
	alpha- humulene	5281520	
	germacrene-D	91723653	
	valencene	9855795	
	viridiflorene	10910653	
	alpha-muurolene	12306047	
	gamma-cadinene	92313	
	1S-cis-calamenene	6429077	
	d-cadinene	441005	
	alpha-calacorene	12302243	
	B-guaiene*	6949	
	ledol	92812	
	spathulenol	92231	
	caryophyllene oxide	1742210	
	globulol	12304985	
	T-cadinol	160799	
	T-Muurolol	3084331	
	torreyol	3084311	
	alpha-cadinol	10398656	
	B-bisabolol	12300146	
	Tetradecanol	8209	
	epi-alpha-bisabolol	1201551	
	benzyl benzoate	2345	
	tetradecanoic acid*	11005	
	hexadecanol	2682	
	hexadecanoic acid*	985	

Plant Name	Phytochemical Name	PubChem CID	Ref
21. *Matricaria chamomilla*	α-Thujene	637518	[52]
	α-Pinene	6654	
	Camphene	6616	
	Sabinene	18818	
	β-Pinene	440967	
	Myrcene	31253	
	α-Phellandrene	7460	
	α-Terpinene	7462	
	o-Cymene	10703	
	Limonene	22311	
	1,8-Cineole	2758	
	(Z)-β-Ocimene	5320250	
	(E)-β-Ocimene	5281553	
	γ-Terpinene	7461	
	Artemisia ketone	68346	
	Terpinolene	11463	
	Linalool	6549	
	allo-Ocimene	5368821	
	Menthone	26447	
	iso-Menthone	6986	
	Menthol	1254	
	Methyl chavicol	8815	
	(3Z)-Hexenyl 2-methyl butanoate	129858869	
	Menthyl acetate	27867	
	Tridecane	12388	
	δ-Elemene	145925530	
	α-Ylangene	6432119	
	α-Isocomene	57501472	
	β-Elemene	482039878	
	(E)-Caryophyllene	5281515	
	β-Copaene	21722369	
	γ-Elemene	12309452	
	dehydro-Sesquicineole	6430797	
	Germacrene D	5317570	
	β-Selinene	519361	
	Bicyclogermacrene	13894537	
	(E,E)-α-Farnesene	5281516	
	γ-Cadinene	92313	
	δ-Cadinene	441005	
	(E)-Nerolidol	5284507	
	Spathulenol	92231	

	α-Bisabolol oxide B	6432283	
	α-Bisabolol	442343	
	α-Bisabolone oxide A	102582076	
	Chamazulene	10719	
	α-Bisabolol oxide A	13092559	
22. *Momordica charantia*	A-Pinene	6654	[53]
	β-Pinene	440967	
	Octanal	454	
	p-Cymene	7463	
	Limonene	22311	
	1,8-Cineole	2758	
	β-Phellandrene	11142	
	Linalool	6549	
	Carvone	7439	
	(E)-Anethole	637563	
	Safrole	5144	
	Methyl eugenol	7127	
	Germacrene D	5317570	
	β-Selinene	519361	
	α-Selinene	10856614	
	δ-Cadinene	441005	
	trans-Nerolidol	5284507	
	Spathulenol	92231	
	Cedrol	65575	
	Apiole	10659	
	3-Decyn-2-ol	536504	[54]
	2,4-Pentanediol	12262	
	Eucalyptol (1,8-Cineole)	2758	
	Methyleugenol	7127	
	Alpha-Humulene	5281520	
	Epiglobulol	11858788	
	Elemol	92138	
	Trimethylbenzene	10686	[55]
	Dichlorobenzene	4685	
	D,L-Limonene	22311	
	Hexanoic acid,2-ethyl-methyl ester	102491	
	2-Hydroxybenzaldehyde	6998	
	Nonanal	31289	
	4-Octenoic acid, methyl ester	5366856	
	2-Aminobenzaldehyde	68255	
	1H-Indole	798	
	Methylanthranilate	8635	
	Isopulegol acetate	94579	[56]
	trans-(+)-Carveol	443178	
	2-Allyl-6-methoxyphenol	347577	
	Aromadendrene	91354	

	Ylangene	20055075	
	Acorenol	11972555	
	1-Hydroxy-cyclohexyl-phenyl-ketone	19989408	
	Cyclopentanetridecanoic acid, methyl ester	554135	
	Hexadecanoic acid, 2,3-dihydroxypropyl ester	14900	
	Caffeine	2519	
	Hexadecanoic acid, methyl ester	8181	
	6-Octadecenoic acid, methyl ester	5366845	
	Octadecanoic acid, methyl ester	8201	
23. *Moringa stenopetala*	Isobutyl isothiocyanate	68960	[57]
	Benzene, 1-isocyano-2-methyl-	140705052	
	Cyclopropane, pentyl-	75640	
	Nonanoic acid	8158	
	Benzyl isothiocyanate	2346	
	δ-Cadinene	441005	
	Myristic acid	11005	
	Methyl palmitate	8181	
	Palmitic acid	985	
	Methyl 9-octadecenoate	8202	
	Oleic acid	445639	
	2,4-Dihydroxy-2,5-dimethyl-3(2H)-furan-3-one	538757	[58]
	p-Dioxane, 2,5-dimethyl-3-methylene	536641	
	4H-Pyran-4-one, 2,3-dihydro-3,5-dihydroxy-6-methyl	119838	
	2,6-Dimethylbenzaldehyde	583841	
	1-Undecanol	8184	
	Phenol, 2,4-bis(1,1-dimethylethyl)	93344	
	Cyclopropanetetradecanoic acid, 2-octyl-, methyl ester	552099	
	1-Hexadecanol	2682	
	2-Propenoic acid, tridecyl ester	18316	
	l-Gala-l-ido-octose	219659	
	Tetradecanoic acid	11005	
	Phytol	5280435	
	Oleic Acid	445639	
	n-Hexadecanoic acid	985	
	Propanoic acid, 3-mercapto-, dodecyl ester	80796	
	2-Methyl-Z,Z-3,13-octadecadienol	5364412	
	Octadecanoic acid	5281	
	Di-(2-Ethylhexyl)phthalate	8343	
24. *Nerium oleander*	2-ethyl-1-Hexanol	7720	[59]
	Nonanoic acid, 9-oxo-, methyl ester	74732	

	Tridecanoic acid, 12-methyl-, methyl ester	21204	
	Tetradecanoic acid	11005	
	Pentadecanoic acid, methyl ester	23518	
	2-Pentadecanone, 6,10,14-trimethyl	10408	
	6-Octen-1-ol, 3,7-dimethyl-, propanoate	8834	
	7,10,13-Hexadecatrienoic acid, methyl ester	5367325	
	7-Hexadecenoic acid, methyl ester, (Z)-	5364431	
	Hexadecanoic acid, methyl ester	8181	
	Isophytol	10453	
	n-Hexadecanoic acid	985	
	Heptadecanoic acid, methyl ester	15609	
	Octadecanoic acid	5281	
	8,11-Octadecadienoic acid, methyl ester	5319737	
	Phytol	5280435	
	Methyl stearate	8201	
	Isopropyl palmitate	8907	
	Oleic Acid	445639	
	cis-Vaccenic acid	5282761	
	9-Octadecenoic acid (Z)-, 2-hydroxy-1-(hydroxymethyl)ethyl ester	5319879	
	7,10,13-Hexadecatrienoic acid, methyl ester	5367325	
	Tricosane	12534	
	Methyl 18-methylnonadecanoate	530340	
	Vitamin E	14985	
	dl- α -Tocopherol	14985	
	Squalene	638072	
	P-cresol	2879	[60]
	4- ethyl-2-methoxy-phenol	62465	
	5-Isopropyl-3,3-dimethyl-2-methylene-2,3-dihydrofuran	586164	
	3-methyl -1H-Indazole	820804	
	2-methoxy-4-vinyl phenol	332	
	2,6-dimethoxyphenyl Benzoate	270567	
	Anobin	538430	
	6,7-epoxypregn-4-ene-9,11,18-triol-3,20-dione, 11,18-diacetate	539836	
	Oleic acid, eicosyl ester	6436542	
	1,15-pentadecanediol	518994	
	Tridecanedial	544162	

25. *Otostegia integrifolia*	2-hexenal	5281168	[61]
	3-hexen-1-ol	5284503	
	1-hexanol	8103	
	α-thujene	17868	
	α-pinene	6654	
	Sabinene	18818	
	1-octen-3-ol	18827	
	3-octanon	246728	
	2-pentylfurane	19602	
	β-myrcene	31253	
	3-octanol	11527	
	p-cymene	7463	
	limonene	22311	
	cis-β-ocimene	5320250	
	phenylacetaldehyde	998	
	trans-β-ocimene	5281553	
	α-terpinolene	11463	
	linalool	6549	
	n-nonanal	31289	
	terpinen-4-ol	11230	
	α-terpineol	442501	
	β-cyclocitral	9895	
	eugenol	3314	
	β-bourbonene	62566	
	trans-caryophyllene	5281515	
	γ-muurolene	6432308	
	germacrene D	5317570	
	bicyclogermacrene	13894537	
	caryophyllene oxide	1742210	
	β-eudesmol	91457	
	α-cadinol	10398656	
26. *Passiflora incarnata*	hexanal	6184	[62]
	benzyl alcohol	244	
	linalool	6549	
	2-phenylethyl alcohol	6054	
	methyl 2-hydroxybenzoate	4133	
	carvone	7439	
	trans-anethole	637563	
	β-ionone	638014	
	α-bergamotol	91749656	
	dodecanoic acid	3893	
	palmitic acid	985	
	Phytol	5280435	
	oleic acid	445639	
	benzene acetonitrile	8794	
	2,3-dihydrobenzofuran	10329	

	isocumene	7668	
	valencene	9855795	
	undecane	14257	
	α-farnesene	5281516	
	neral	643779	
	geranial	638011	
	camphor	2537	
	menthone	26447	
	isomenthone	6432469	
	beta-Cyclocitral	9895	
	α-Angelica lactone	11559	
	4-hydroxybenzaldehyde	126	
	maltol	8369	
	eicosanol	12404	
	linalyl acetate	8294	
	propyl dodecanoate	77255	
	Acetic acid	176	
27. *pastinaca sativa*	Butyl butyrate	7983	[63]
	(Z)-β-Ocimene	5320250	
	Octanal	454	
	Hexyl butyrate	17525	
	Octyl acetate	8164	
	Decanal	8175	
	Octanol	957	
	Octyl butyrate	61030	
	α-Zingiberene	521253	
	Decanol	8174	
	β-Sesquiphellandrene	519764	
	ar-Curcumene	92139	
	Octyl hexanoate	21006	
	Decyl butyrate	229387	
	Benzyl butyrate	7650	
	Phenylethyl butyrate	7658	
	(E)-Nerolidol	5284507	
	Phenylethyl hexanoate	103018	
	Psoralen	6199	[64]
	Isobergapten	68082	
	Methoxsalen	4114	
	Bergapten	2355	
	Pimpinellin	4825	
	Isopimpinellin	68079	
	Imperatorin	10212	
	Heraclenin	458010	
	Phellopterin	98608	
	Byakangelicol	3055167	
	Heraclenol	73253	

	Isobyakangelicin	21826768	
	Byakangelicin	10211	
28. *Pentas schimperiana*	saponins	6540709	[65]
	flavonoids	135332484	
	tannins	16133892	
29. *Phyllanthus emblica*	Hentriacontane	12410	[66]
	Dotriacontane	11008	
	Tetracontane	20149	
	Tritetracontane	522398	
	Pentacosane	12406	
	Octadecanoic acid, Methyl Ester	8201	
	Vitamin E	14985	
	Tetracontane-1,40-diol	557624	
	Hexacosanol, Acetate	3083648	
	Gamma Sitosterol	133082557	
	Citronellyl propionate	8834	[67]
	1-Methyl -4 isopropyl-cyclohexyl 2-hydroperfluorobutanoate	565481	
	Citronellyl acetate	9017	
	Bicyclo(2.2.1) heptane,2,2,3-trimethyl-endo	296894	
	Hexadecanoic acid, methyl ester	8181	
	Tridecanoic acid, methyl ester	15608	
	Pentadecanoic acid, 14- methyl, methyl ester	21205	
	Decanoic acid, methyl ester	8050	
	N-Hexatriacontane	12412	
	Stigmast-5-en-3-ol,oleate	20831071	
	N-Hexacosane	12407	
	Ergost -5-en-3-ol,acetate,(3,beta,24R)-	13019955	
	N-Tetracosane	12592	
	Oleyl alcohol	5284499	
	2(1H)-Benzocyclooctenone,decahydro-10a-methyl-,trans	41325	
	Lanost-8-ene	22296726	
	Ethanone,1,1'-(6-hydroxy-2,5-benzofurandiyl)bis-	104654	
	8-Amino-5-benzyloxy-6-methoxy-4-methylquinoline	609005	
	1-Naphthalenol,decahydro-4a-methyl-8-methylene-2-(1-methylethyl)-,acetate ,(1S-(1.alpha.,2.beta.,4a.alpha.,8a.alpha.))-	101280483	
	Decanoic acid, trimethylsilyl ester	521628	[68]

30. *Pimpinella anisum*	Dodecanoic acid, trimethylsilyl ester	521640	
	n-Pentadecanoic acid, trimethylsilyl ester	523679	
	Fatty acid C16:1	445638	
	Hexadecanoic acid, trimethylsilyl ester (Palmitic acid)	521638	
	Linoleic acid, trimethylsilyl ester	5352430	
	9,12-Octadecadienoic acid (linoleic acid) + Oleic acid	6439696	
	Octadecanoic acid, trimethylsilyl ester	87777	
	Nonadecanoic acid, TMS	522538	
	Octadecadienoic acid,TMS	5352430	
	Eicosanoic (Arachidic) acid, TMS	521648	
	Hexadecanoic acid, 2,3-bis[(trimethylsilyl)oxy]propyl ester	552033	
	Docosanoic (behenic) acid, TMS	522539	
	Octadecanoic acid, 2,3-bis[(trimethylsilyl)oxy]propyl ester	553936	
	Tetracosanoic acid, trimethylsilyl ester	522540	
	Hexacosanoic acid TMS	526089	
	Malic acid, tris (trimethylsilyl) ester	522155	
	Heptanedioic acid TMS	129724577	
	Octanedioic acid	10457	
	Benzaldehyde, 3-methoxy-4-[(trimethylsilyl)oxy]-	522236	
	Benzeneacetic acid, 4-[(trimethylsilyl)oxy]-, trimethylsilyl ester	520335	
	Benzaldehyde, 3,5-dimethoxy-4-[(trimethylsilyl)oxy]-	614155	
	Benzoic acid, 3-methoxy-4-[(trimethylsilyl)oxy]-, trimethylsilyl ester	553664	
	Azelaic acid, bis (trimethylsilyl) ester	519492	
	Inositol, 1,2,3,4,5,6-hexakis-O-(trimethylsilyl)-, scyllo-	520232	
	Sterol	1107	
	Campesterol TMS	22216479	
	Stigmasterol trimethylsilyl ether	91698162	
	β-sitosterol trimethylsilyl ether	582434	
	Sitosterol – glucopyranoside	12309068	
	β-Amyrin TMS	612816	
	Lupeol	259846	
	Betulinic acid	64971	
	Ursolic acid	64945	
	Octadec-9Z-enol TMS ether	5366884	

31. *Pistacia lentiscus*	Isoledene	530426	[69]
	3-carene	26049	
	α-phellandrene	7460	
	Verbenol	61126	
	α-pinene	6654	
	Linalool	6549	
	β-pinene	440967	
	p-cymen-8-ol	14529	
	Terpinolene	11463	
	Limonene	22311	
	Terpinene-4-ol	11230	
	β-caryophyllene	5281515	
	Cis-ocimene	5320250	
	α-farnesene	5281516	
	α-terpineol	17100	
	Camphor	2537	
	Borneol	64685	
	Spathulenol	92231	
	Camphene	6616	
	Myrcene	31253	
	Globulol	12304985	
	Lupeol	259846	[70]
	Lupenone	92158	
	Lupanol	129649741	
	Farnesol	445070	
	β-Sitosterol	222284	
	α-Tocopherol	14985	
	Cubebol	11276107	
	δ-Amorphene	10223	
	δ-Cadinene	441005	
	α-Muurolene	12306047	
	α-Humulene	5281520	
	Germacrene D	5317570	
	trans-muurola-4(14),5- diene	91747125	
	β-Cubebene	93081	
	Tricyclene	79035	[71]
	Camphene	6616	
	Sabinene	18818	
	Eucalyptol	2758	
	Allo-Aromadendrene	42608158	
	Ylangene	20055075	
	α-cadinol	10398656	
	Bornyl acetate	93009	
	Isoamyl butyrate	7795	
32. *Pterocarpus marsupium*	Saponin	198016	[72]
	Phytosterols	12303662	

	Tannins	16133892	
	Cardiac glycosides	439501	
	delta.-Selinene	520383	[73]
	n-Hexadecanoic acid	985	
	9,12-Octadecadienoic acid (Z,Z)-	5280450	
	Furan-2-one,3,4-dihydroxy-5-[1-hydroxy-2- fluoroethyl]	54713375	[74]
	Hydrazine, 1-methyl-1-(2-propynyl)	542407	
	Cyclopentasiloxane,decamethyl	10913	
	Benzoic acid, 2,6-bis[(trimethylsilyl)oxy]- trimethylsilyl ester	520869	
	3',8,8'-Trimethoxy-3-piperidyl-2,2'-binaphthalene-1, 1',4,4'-tetrone	590815	
	9-(2',2'-Dimethylpropanoilhydrazono)-3,6-dichloro-2,7-bis-[2-(diethylamino)-ethoxy]fluorene	590814	
	Phthalic acid, di(oct-3-yl)ester	15764573	
	Rhodopin	5365880	
	Bis(cis-13-docosenamido)methane	5365356	
	9-Octadecenamide,(Z)	5283387	
	Ethyl iso-allocholate	6452096	
	9,12,15-Octadecatrienoic acid, 2,3-bis[(trimethylsilyl) oxy]propyl ester,(Z,Z,Z)	5366384	
	1-Penten-3-one,1-(2,6,6- trimethyl-1-cyclohexen-1- yl)-	5375218	[75]
	Geranyl isovalerate	5362830	
	2-Pentadecanone,6,10,14- trimethyl	10408	
	Octadecanoic acid	5281	
	Phytol	5280435	
	Heptadecanoic acid,16- methyl,-methyl ester	110444	
	4,8,12,16- Tetramethylheptadecan-4-olide	567149	
	Dasycarpidan-1- methanol,acetate	101287780	
	Cyclopropaneoctanal,2-Octyl-	550143	
34. *Ptychotis verticillata*	α-Thujene	637518	[76]
	α-Pinene	6654	
	γ-Terpinene	7461	
	Sabinene	18818	
	β-Pinene	440967	
	Myrcene	31253	
	p-Cymene	7463	

	Limonene	22311	
	1,8-Cineole	2758	
	Linalol	6549	
	Borneol	64685	
	Terpinen-4-ol	11230	
	α-Terpineol	442501	
	Carvacryl methyl ether	80790	
	Thymol	6989	
	Carvacrol	10364	
	α-Terpinyl acetate	111037	
	Geranyl acetate	1549026	
	Caryophyllene oxyde	14350	
	beta.-Phellandrene	11142	[77]
	beta.-Myrcene	31253	
	D-Limonene	440917	
	m-Cymene	10812	
33. *Spondias pinnata*	Hexanal	6184	[78]
	Furfural	7362	
	2-Hexenal	5281168	
	(Z)-3-Hexen-1-ol	5281167	
	Camphene	6616	
	α-Pinene	6654	
	5-Methyl-2-furaldehyde	12097	
	β-Pinene	440967	
	β-Myrcene	31253	
	3-Carene	26049	
	(3E)-Hexenyl acetate	5352557	
	Isocineole	10106	
	p-Cymene	7463	
	Limonene	22311	
	Benzeneacetaldehyde	998	
	(E)-β-Ocimene	5281553	
	γ-Terpinene	7461	
	Linalool oxide	22310	
	2-Furaldehyde diethyl acetal	83553	
	(E)-Linalool oxide, furanoid	6432254	
	Benzoic acid, methyl ester	7150	
	Linalool	6549	
	Nonanal	31289	
	2-Fenchanol	15406	
	1-Terpineol	11468	
	Ocimenol	5368785	
	Isoborneol	6321405	
	Ethyl benzoate	7165	
	Terpinen-4-ol	11230	
	α-Terpineol	442501	

	Methyl salicylate	4133	
	(E)-2-Decenal	5283345	
	β-(E)-Damascenone	5366074	
	Caryophyllene	5281515	
	Ethyl cinnamate	637758	
	γ-Eudesmole	91730386	
	Ethyl hexadecanoate	12366	
	Squalene	638072	[79]
	Tetracosanal	557529	
	n-Nonacosane	12409	
	n-Octacosane	12408	
	n-Heptacosane	11636	
	n-Eicosanol	12404	
	n-Tricosane	12534	
	n-Tetracosane	12592	
	n-Pentacosane	12406	
	Khusimone	6428327	
35. *Tanacetum vulgare*	santolina triene	519872	[80]
	tricyclene	79035	
	α-Thujene	637518	
	α-Pinene	6654	
	Camphene	6616	
	Sabinene	18818	
	β-Pinene	440967	
	2,3-dehydro-1,8-cineole	523035	
	yomogi alcohol	5315406	
	p-Cymene	7463	
	1,8-Cineole	2758	
	α-Terpinene	7462	
	γ-Terpinene	7461	
	α-Thujene	637518	
	artemisia ketone	68346	
	(Z)-sabinene hydrate	62367	
	artemisia alcohol	100197	
	terpinolene	11463	
	β-thujone	91456	
	chrysanthenone	442463	
	sabinol	94147	
	camphor	2537	
	pinocarvone	121719	
	chrysanthenol	527032	
	artemisyl acetate	524254	
	borneol	64685	
	4-terpineol	11230	
	(E)-chrysanthenyl acetate	162747	
	bornyl acetate	93009	

	(E)-sabinyl acetate	6430313	
	alpha-terpineol	17100	
	alpha-Gurjunene	15560276	
	pentadecane	12391	
	germacrene D	5317570	
	alpha-caryophyllene	5281520	
	beta-caryophyllene	5281515	
	(E)-nerolidol	5284507	
	spathulenol	92231	
	δ-cadinene	441005	
	caryophyllene oxide	1742210	
	davanone	519782	
	τ-muurolol	3084331	
	alpha-cadinol	10398656	
36. *Tinospora cordifolia*	n-Hexadecanoic acid	985	[81]
	Heptadecanoic acid	10465	
	Stigmasterol	5280794	
	9,12- Octadecadienoic acid (Z,Z)-	5280450	
	Phytol	5280435	
	1-Heptacosanol	74822	
	Octadecanoic acid	5281	
	Hexadecanoic acid, methyl ester	8181	
	Icosanoic acid	10467	
	Cholest-4-en-3- one	91477	
	Isopinocarveol	10931630	[82]
	α-ylangene	6432119	
	Caryophyllene	5281515	
	trans-Z-α-Bisabolene epoxide	91753504	
	Benzene, 1-(1,5-dimethyl-4-hexenyl)-4-methyl	92139	
	trans-α-Bergamotene	6429302	
	β-Bisabolene	10104370	
	β-Cubebene	93081	
	.(+)-Sativen	530427	

	Methyl 4,7,10,13-hexadecatetraenoate	14122946	
	Alloaromadendrene oxide-(1)	528759	
	α-acorenol	11972555	
	7-epi-cis-sesquisabinene hydrate	6428435	
	2,5-Octadecadiynoic acid, methyl ester	42151	
	Phenol, 2-methyl-5-(1,2,2-trimethylcyclopentyl)-, (S)-	11790670	
	5-Isopropyl-2,8-dimethyl-9-oxatricyclo[4.4.0.0(2,8)]decan-7-one	565717	
	Hexadecanoic acid, ethyl ester	12366	
	17-Octadecynoic acid	1449	
	Z,Z,Z-4,6,9-Nonadecatriene	5362863	
	n-Propyl cinnamate	5270647	
	Dasycarpidan-1-methanol, acetate (ester)	550072	
	Piperine	638024	
37. *Trigonella foenum*	2-Methylpyrrolidine	13003	[83]
	Dodecane	8182	
	α-Terpinene	7462	
	α-Terpinyl acetate	111037	
	Tetradecane	12389	
	Caryophyllene	5281515	
	Pentadecane	12391	
	Trichloroacetic acid, pentadecyl ester	522535	
	Phytol	5280435	
	Nonadecane	12401	
	Palmitic acid or n-Hexadecanoic acid	985	
	Eicosane	8222	

	Linoleic acid methyl ester	5284421	
	Linoleic acid	5280450	
	4-Pentyl-1-(4- propylcyclohexyl)-1-cyclohexene	557007	
	1-Piperidinepropanenitrile	18338	
	Palmidrol	4671	
	Arachidic Acid or Eicosanoic acid	10467	
	Glyceryl 2-linoleate	5365676	
	(R)-(-)-(Z)-14-Methyl-8- hexadecen-1-ol	12487634	
	Quinoline	7047	
	Tetratriacontane	26519	
	Carvone	7439	[84]
	Stigmasterol	5280794	
	Campesterol	173183	
	Nonaethylene glycol	4867	
	Limonene	22311	
	Hexadecanoic acid,2-hydroxy-1-(hydroxymethyl)ethyl ester	123409	
	1-Monolinoleoylglycerol-trimethylsilyl ether	5366692	
	Cedryl propyl ether	591419	
	Methyl stearate	8201	
	Hexadecanoic acid, methyl ester	8181	
	n-Cetyl alcohol	2682	
	Dimethyl phthalate	8554	
	2,4,7,9-Tetramethyl-5-decyn-4,7-diol	31362	
	3,5-Octadiene	5352266	[85]
	p-Xylene	7809	

	δ-3-Carene	26049	
	Heptanal	8130	
	3-Isopropyltoluene	10812	
	Limonene	22311	
	Decanal	8175	
	1-Methoxy-4-(2-propenyl)-benzene	8815	
	2-Methyl-5-isopropylphenol	10364	
	(2E, 4E)-Decadienal	5283349	
	Trans-anethole	637563	
	5-Pentyl-2(5H)-furanone	89559	
	Cis-calamenene	6429077	
	Cadina-1,4-diene	6427091	
	Hexadecanoic acid	985	
	(9E, 12E)-Octadecadienoic acid	5282457	
	β-Thujone	91456	

38. *Abrus precatorius*	3,7,11,15-Tetramethyl-2-hexadecen-1-ol	5366244	[86]
	n-Hexadecanoic acid	985	
	Phytol	5280435	
	9,12,15-Octadecatrienoic acid, (Z,Z,Z)-	5280934	
	Octadecanoic acid	5281	
	Hexanedioic acid, mono(2-ethylhexyl)ester	20342	
	Heneicosane, 11-(1-ethylpropyl)-	292291	
	Squalene	638072	
	Heptadecane, 9-octyl-	292286	
	17-Pentatriacontene	5365022	
	Ergosterol	444679	
	(Cholestane-3,5-diol, 5-acetate, (3beta,5alpha)-)	249903077	
	Stigmasterol	5280794	
	Dimethyl Sulfoxide	679	[87]
	Octadecanal	12533	
	9-OxononanoicAcid	75704	
	Squalene	638072	
	DI-N-Decylsulfone	19089489	
	2H-1-Benzopyran-6-OL, 3,4-Dihydro-2,5,7,8-Tetramethyl-2-(4,8,12-Trimethy	86472	
	1,2-BIS(Trimethylsilyl)Benzene	519794	
	5,17-Dotriacontadiyne	577953	
	Decanal	8175	[88]
	2-Decenal, (E)-	5283345	
	Nonanoic acid	8158	
	Undecanal	8186	
	2-Undecenal	5283356	
	Dodecanal	8194	
	Tridecanal	25311	
	Tetradecanal	31291	
	Pentadecanal	17697	
	Heneicosane	12403	
	Hexadecanoic acid, methyl ester	8181	
	Octadecanoic acid, 2-propenyl ester	80500	
	9-Octadecenoic acid, methyl ester, (E)-	5280590	
	2(3H)-Furanone, 5-dodecyldihydro-	97747	
	Palmitic acid vinyl ester	69658	
	Pentacosane	12406	
	Tetracontane	20149	
	Tetratriacontane	26519	
	Furan, 2-butyltetrahydro	10877221	
	3-Hexanone	11509	
	2-Hexanone	11583	
	3-Hexanol	12178	

	2-Hexanol	12297	
	Octanoic acid	379	
	Nonane, 2-methyl-5-propyl-	545954	
	Vinyl caprylate	13164	
	Trifluoroacetic acid, n-tridecyl ester	536357	
	Decanoic acid, 2-propenyl ester	93831	
	Bis(2-ethylhexyl) phthalate	8343	
	Methyl stearate (methyl octadecanoate	86753301	[89]
	9-Octadecenoic acid	445639	
	9-Octadecenoic anhydride	90647	
	15-Hydroxypentadecanoic acid	78360	
	Oleic anhydride	5369123	
	Octadecanoic acid, 2,3-dihydroxypropyl ester	24699	
	4,22-Stigmastadiene-3-one	5364563	
39. *Acacia arabica*	Hexadecanoic acid, methyl ester	8181	[90]
	n-Hexadecanoic acid	985	
	1H-3A,7-methanoazulen-6-ol	21192052	[91]
	1H-benzocycloheptene	21319341	
	diethyl phthalate	6781	
	1-(4-isopropylphenyl)-2-methylpropyl acetate	154574010	
	13-hexyl-oxa-cyclotridec-10-en-2-one	566650	
	9,12-octadecadienoic acid	3931	
	6-octadecenoic acid, methyl ester	40328	
	methyl stearate	8201	
	E,E,Z-1,3,12-nonadecatriene-5,14-diol	5364768	
	hexahydro-3-butylphthalide	12367055	
	cis-11-eicosenoic acid, methyl ester	5463047	
	cis-13-eicosenoic acid, methyl ester	14122961	
	eicosanoic acid, methyl ester	14259	
	13-docosenoic acid	8216	
	docosanoic acid	8215	
	tetracosanoic acid	11197	
	Cyclopentadecanone	10409	
	Octacosan	12408	
	9-octadecenal	5283381	
	Stigmasterol	5280794	
	Lupeol	259846	
	Ethanol,2-(trimethylsilyl)-	18399	[92]
	1-Methyl-2-tert-butylpyrrole	578990	
	Silane,dimethoxydimethyl-	66187	
	Benzene,(1,2,2-trimethoxyethyl)-	554650	

	Ethanone,2,2-dimethoxy-1,2-diphenyl	90571	
40. *Acacia catechu*	2-Aminobenzophenone	76080	[93]
	1, 3, 5-triphenyl-1, 5-pentanedione	138708	
	3-Aminobenzophenone	17817	
	Benzimidazole	5798	
	Phosphine imide	135674086	
41. *Artocarpus heterophyllus*	Quinoline, 7-propyl-	588472	[94]
	o-Aminobenzohydroxamic acid	79721	
	1,3-Benzodioxole, 2-methoxy	573476	
	N,N-Dimethyl-6-benzyloxyhexylamine	547332	
	6,9-Octadecadiynoic acid, methyl ester	560938	
	1,4-Pentanediamine, N1,N1-diethyl	78953	
	Propane, 1-(2,2-dichloro-1,3,3- trimethyl	6564	
	2-Methoxy-4-vinylphenol	332	
	Oxazolidinecarboxylic acid	13419880	
	Coumarin, 3,4-dihydro-4,4,7-trimethyl-	600374	
	9-Aminofluorene	10671	
	Megastigmatrienone	5375190	
	1-Methyl-3,5-diisopropoxybenzene	580245	
	Pentadecanoic acid, 14-methyl-, methyl	21205	
	Hexadecanoic acid, octadecyl ester	75778	
	Ethyl 5,8,11,14,17-icosapentaenoate	5353613	
	Bis (2-ethylhexyl) phthalate	8343	
	gamma.-Sitosterol	457801	
	Octane, 2,7-dimethyl	14070	[95]
	Germacrene	9548705	
	1-Dodecene	8183	
	trans-Caryophyllene	5281515	
	alpha-amorphene	12306046	
	Farnesol	445070	
	Undecane	8182	
	γ-Cadinene	6432404	
	3,7-Dimethyl-octa-1,7-dien-3,6-diol	548927	
	(-)-Caryophyllene oxide	1742210	
	1-Undecene	13190	
	11-tetradecen-1-ol,acetate, (Z)-	5367692	
	2-Pentadecanone,6,10,14-trimethyl	10408	
	Octadecanoic acid	5281	
	Cyclopropanepentanoicacid, 2-undecyl-, methyl ester, tran	91691232	
	9-Octadecenamide, (Z)	5283387	
	Phenol, 3,4,5-trimethoxy-	69505	[96]
	4-((1E)-3-Hydroxy-1-propenyl)-2-methoxyphenol	1549095	
	Scopoletin	5280460	
	10,12-Octadecadienoic acid	5280802	
	Hexadecanoic acid, ethyl ester	12366	

	8-Oxohexadecanoic acid	5363269	
	Ethyl oleate	5363269	
	Hexadecanamide	69421	
	Ethyl 9,12-hexadecadieno-ate	5280450	
	1H-Pyrido[3,4-b]indol-1-one, 2,3,4,9-tetrahydro-	87371	
	Hexadecanoic acid, 2-hydroxy-1-(hydroxymethyl) ethyl ester	129853056	
	Pyrazolo[3,4-b]thiopyrano[4,3-d]pyridin-1-1-amine, 3,6,8,9-tetrahydro-8,8-dimethyl-5-phenyl-	16757237	
	10,11-Dihydro-10-hydroxy-2,3-dimethoxydibenz(b,f) oxepin	623334	
	Butyl 9,12-octadecadienoate	6436910	
	Octadecanoic acid, 2-hydroxy-1-(hydroxymethyl) ethyl ester	79075	
	Stigmast-4-en-3-one	5484202	
	Methanone, (5-hydroxy-3-benzofuryl)(2,5-dimethoxyphenyl)	626866	
	2,4a,8,8-Tetramethyldecahydrocyclopropa[d]naphthalene	579617	
	2,2,4-Trimethyl-3-(3,8,12,16-tetramethyl-heptadeca- 3,7,11,15-tetraenyl)-cyclohexanol	5366008	
42. *Aerva lanata*	3-O-methyl-D -glucose	8973	[97]
	1,2-Benzenedicarboxylic acid, diisooctyl ester	33934	
	Hydroquinone monobenzyl ether	7638	[98]
	1-(2-Hydroxyethoxy) tridecane*	38049	
	Pent-4-enal	16418	
	2-Methylene-3-(2-methylpropyl)cyclopentanone	84107266	
	(2R,6R)-2(tert-Butyl)-6-methyl-1,3-dioxane-4-one	11052102	
	1,10-Decanediol	37153	
	Propanal, oxime	641641	
	5-t-Butyl-2-(5H)-furanone	12374806	
	Pentadecane,2,6,10-trimethyl-	19775	
	Docosane	12405	
	Bis-(3,5,5-trimethylhexyl)ether	57048725	
	6H-Dibenzo[b,d]thiopy ran	308189	
	Dotriacontane	11008	
	2-Oxetanone	2365	
	3-Tert-butyl-5-methylhex-4-en-2-ol	85365089	
	(R,Z)-12- Hydroxy-9- octadecenoic acid	643684	
	Nonanoic acid, methyl ester	15606	
	1-(2,3- Epoxypropionyl) cyclohexanol	10103578	
	Methyl 2- Cyanohept-6- ynoate	15179027	
	Phenyl 2- butenoate	549509	
	3-Methyl-3-butenyl acetate	78879	
	Tetradecanoic acid, methyl ester	31284	
	2-Hydroxyethyl cycloheptane carboxylate	85977994	
	Hexanedioic acid, monomethyl ester	12328	
	1,6 - anhydro - beta - D - gluco - furanose	13037722	

43. *Aegle marmelos*	Hexatriacontane	12412	[99]
	Oxalic acid, Cyclobutyl nonyl ester	6420614	
	Sulfurous acid, 2-Propyl tridecyl ester	6420355	
	Eicosane, 2-Methyl	519146	
	Guanidine, Methyl	10111	
	Eicosane, 7-Hexyl	292289	
	N-Propyl Decyl Ether	17785621	
	1-Butanol, 4-(1-Methylethoxy)-	537092	
	1-Iodo-2-Methylundecane	545590	
	Nonadecane	12401	
	Octadecane, 3-ethyl-5-(2-Ethylbutyl)-	292285	
	Heptacosane	11636	
	Tetracosane, 11-Decyl	294707	
	Tetratetracontane	23494	
	Octadecane, 3-Ethyl-5-(2-Ethylbutyl)	292285	
	2-Bromotetradecane	12798926	
	Eicosane, 9-Octyl	280905	
	PENTAN-2-ONE	7895	[100]
	1-penten-3-ol	12020	
	Cyclopentanol	7298	
	p-mentha-1 (7),3-diene	66841	
	8-hexadecenal	6430170	
	1-dodecanol	8193	
	Cyclooctasiloxane	18993663	
	Tetradecamethylcycloheptasiloxane	7874	
	Cyclononasiloxane	53438479	
	Methyl linolelaidate	5362793	
	9,17-octadecadienal	6431297	
	Hentriacontane	12410	
	Oleyl alcohol	5284499	
	cis-13-eicosenoic acid	5312518	
	Nonahexacontanoic acid	38626	
	Benzoic acid	243	
	1-heptadec-1-ynyl-cyclopentanol	536340	
	7-pentadecyne	549063	
	Tetracosamethyl-cyclododecasiloxane	167767	
	15-tetracosenoic acid	5281120	
	Retinoic acid	444795	
	Cyclodecasiloxane	519601	
	Farnesyl acetone	1711945	
44. *Aframomum melegueta*	(S)-2-Heptanol	2724897	[101]
	a-Pinene	11240513	
	b-Pinene	14896	
	(S)-2-Heptyl acetate	13141748	
	(E)-Ocimene	5281553	
	(R)-Linalool	443158	

	(E)-4,8-Dimethyl-1,3,7-nonatriene	6427110	
	b-Caryophyllene	5281515	
	Humulene	5281520	
	Germacrene-D	91723653	
	Bisabolene	3033866	
	Gingerol	442793	[102]
	Caryophylene	1742210	
	Humulene	5281520	
	DL-Arabinose	854	
45. *Ageratum conyzoides*	2-propenoic acid 3-(2-hydroxyphenyl)- (E)-	637540	[103]
	Precocene 1	28619	
	2(4H)-Benzofuranone,5,6,7,7a-tetrahydro-4,4,7a-trimethyl-	157995	
	Cadala-1(10)3,8-triene	593889	
	Fumaric acid, ethyl-2-methylallyl ester	5461492	
	Caryophyllenyl alcohol	91704770	
	Cubenol	11770062	
	Isoaromadendrene epoxide	534398	
	Globulol	12304985	
	4-(1,3,3-trimethyl-bicyclo[4.1.0]hept-2-yl-but-3-en-2-one	5370102	
	Rotundene	25203405	
	Phthalic acid cyclohexyl pentyl ester	6423869	
	Terephthalic acid phenyl undecyl ester	91741775	
	Terephthalic acid 3,4-dichlorophenyl undecyl ester	91741773	
	Pregnan-16-one	57488115	
	Tricosane	12534	
	2-pentacosanone	547856	
	4,8,12,16-tetramethylheptadecan-4-olide	567149	
	4(1H)-Pyrimidinone 6-ethoxy-2-phenyl	608431	
	2-Undecanone 6,10-dimethyl-	95495	
	13-Docosenamide	5365371	
	a-Amyrin	73170	
	Friedelan-3-on	469617998	
	1-Hexadecanol, 2-methyl-	17218	[104]
	11-Octadecenoic acid, methyl ester	5364432	
	Morphinan, 7,8-didehydro-4,5-epoxy-3,6-dimethoxy-17-methyl-, (5.alpha, 6.alpha)-	5492619	
	1,2-Benzenedicarboxylic acid, mono(2-ethylhexyl) ester	20393	
	chondrillasterol 3	5283663	[105]

46. *Albizia adianthifolia*	9,12-octadecadienoic acid (Z,Z)-, methyl ester 6	5284421	
47. *Albizia lebbeck*	2,4-Dimethyl hexane	11511	[106]
	Ethyl cyclohexane	15504	
	Ethyl benzene	7500	
	9Hy	129626294	
	Propylcyclohexane	15505	
	Ethyl-3-methyl benzene	12100	
	Methyl benzene	1140	
	n-Decane	15600	
	1H-Indene	7219	
	Linalool	6549	
	Isopropyl benzene	7406	
	n-Octyl formate	8176	
	α-Gurjunene	481115168	
	2,5-Pentadecadien-1ol	5364952	
	Hexadecanal	984	
48. *Allium cepa*	methyl pyruvate	11748	[107]
	3-hydroxymethylfuran	20449	
	propylene glycol	1030	
	catechol	289	
	5-hydroxymethylfurfural	237332	
	2,4-Dimethylthiophene	34296	[108]
	3,4-Dimethylthiophene	79089	
	Dimethyl trisulfide	19310	
	Dipropyl disulfide	12377	
	Propenyl propyl disulfide	5320722	
	Methyl propyl trisulfide	5319765	
	Dipropyl trisulfide	22383	
	Ergost-8-en-3-ol	6452868	[109]
	Cholesta-4,6-dien-3-ol	53996943	
	Myristic acid	11005	
	Ergost-8-en-3-ol, 14-methyl-, (3 beta,5 alpha.)-	22296740	
	Fumaric acid	444972	
	Oxalic acid	971	
	2-Mercapto-3,4-dimethyl-2,3-dihydrothiophene	529389	
	3(2H)-Furanone	529536	
	Thiophene	8030	
	Ethanone	643972	

	1,2-Dimethoxy-4-(2-methoxyethenyl)benzene	71341351	
	7,9-Di-tert-butyl-1-oxaspiro(4,5)deca-6,9-diene-2,8-dione	545303	
	9,19-Cyclolanost-24-en-3-ol	129660864	
	1-Hexadecyne	12396	
	Dibutyl phthalate	3026	
	Heptadecanoic acid	10465	
	Allicin	65036	
49. *Allium sativum*	Morellinol	5364072	[110]
	Estrone0	5870	
	Phentolamine	5775	
	Nonivamide	2998	
	Gallic acid	370	
	m-Toluic acid	7418	
	p-Toluic acid	7470	
	Butylated hydroxytoluene	31404	
	1-(4-Hydroxy-3-methoxyphenyl)dec-3-en-5-one	73064716	
	3,4-Dihydroxymandelic acid	85782	
	1,2 Benzenediol	289	
	Alliin	87310	[111]
	Allyl methyl disulphide	62434	
	Allyl methyl sulphide	66282	
	Dimethyl disulfide	12232	
	Diallyl ether	11194	
	Trichloroethylene	6575	
	Diallyl sulfide	11617	[112]
	trans-Propenyl methyl disulfide	5366552	
	Methyl allyl trisulfide	61926	
	Diallyl trisulfide	16315	
	Eugenol	3314	
	Aromadendrene	91354	
	Diallyl tetrasulfide	75552	
	Elemicin	10248	
50. *Aloe vera*	p-Xylene	7809	[113]
	1,5-Heptadien-4-one, 3,3,6-trimethyl-	68346	
	1-Heptanol, 2-propyl-	24847	
	Tridecane	12388	
	7-Tetradecene, (Z)-	5364650	
	Tetradecane	12389	
	Hexadecane	11006	
	12,15-Octadecadiynoic acid, methyl ester	538453	

	(4,7-Dinitronaphthalen-1-yl)- (4-methoxyphenyl) diazene	572336	
	9-Octadecenoic acid, (2-phenyl-1,3-dioxolan-4-yl)methyl ester,cis-	21160048	
	9,12,15-Octadecatrienoic acid, 2-[(trimethylsilyl)oxy]-1-[[(trimethylsilyl)oxy]methyl]ethyl ester,(Z,Z,Z)-	5362857	
	1,2-Benzenedicarboxylic acid, butyl octyl ester	66540	
	11,14-Eicosadienoic acid, methyl ester	5365566	
	Eicosane	8222	
	4-Mercaptophenol	240147	[114]
	5-Acetoxymethyl -2-furaldehyde	66349	
	4,5-Dimethyl-4-hexene-3-one	519408	
	Alpha-D- Glucose	79025	
	Tridecanoic Acid	12530	
	Maltotriose	439586	
	Pentadecanoic Acid	13849	
	Palmitic anhydride	69339	
	Glyceryl 1, 3- Dipalmitate	68149	
	12-Hydroxy-8-(1- Hydroxyethyl) Heptadecanoic Acid	20322272	
	Triarachine	522017	
	6,9,12,15-Octadecatetrae-noic Acid, Ethyl Ester	6450798	
	Ethyl stearate	8122	
51. *Ammi visnaga*	Hexanoic acid, methyl ester	7824	[115]
	Capric acid, methyl ester	8050	
	Lauric acid, methyl ester	8139	
	Palmitoleic acid, methyl ester	643801	
	Margaric acid, methyl ester	15609	
	Oleic acid, methyl ester	5364509	
	Elaidic acid, methyl ester	5280590	
	Behenic acid, methyl ester	13584	
	Tetracosanoic acid, methyl ester	75546	
	Nonacosane	12409	[116]
	Octadecyl icosanoate	89710	
	Methoxsalen	4114	
	Octadecyl stearate	17720	
	Pimpinellin	4825	
	Heptadecane	12398	
	Marmesin	334704	
	Tetracosane	12592	
	Icosyl stearate	89703	
	Oleoylglycerol	5283468	
	Tocopherol=vitamin E	14985	

	Cetyl oleate	5377655	
	9Z-9-Tricosene	5365075	
	Isopimpinellin	68079	
	Psoralen	6199	
	Bergapten	2355	
	Arachidic Acid	10467	
52. *Anacradium occidentale*	Methyl but-2-enoate	12181	[117]
	Toluene	1140	
	Hexyl acetate	8908	
	Isobutyl acetate	8038	
	4,4-Dimethylheptane	136827	
	Furfural	7362	
	(E)-Hex-2-enal	5281168	
	Z)-Hex-3-en-1-ol	87244822	
	1-Hexanol	8103	
	Tricyclen	79035	
	Ethyl hexanoate	31265	
	Octanal	454	
	Phenylacetaldehyde	998	
	Acetophenone	7410	
	3-Methylbutyl 3-methylbutanoate	12613	
	1-Nonanol	8914	
	Methyl salicylate	4133	
	cis-3-Hexenyl 3-methylbutanoate	37065	
	a-Cubebene	442359	
	a-Copaene	12303902	
	Phenylmethyl pentanoate	82584	
	(E)-Cinnamic acid	444539	
	2-Methylbutyl benzoate	103653	
	allo-Aromadendrene	42608158	
	Germacrene D	5317570	
	b-Selinene	519361	
	Valencene	9855795	
	Bicyclogermacrene	13894537	
	Benzyl tiglate	250096	
	Cadina-1,4-diene	6427091	
	a-Cadinene	12306048	
	Selina-3,7(11)-diene	522296	
	Spathulenol	92231	

53. *Cola nitida*	Caffeine	2519	[118]
	Palmitic acid	985	
	Linoleic acid	5280450	
	Oleic acid	445639	
	Stearic acid	5281	
	10,12-Octadecadienoic acid	5282801	
	7-(Tetrahydro-2H-pyran-2-yloxy)-2-octyn-1-ol	558995	
	2-Octylcyclopropene-1-heptanol	534620	
	8-Oxohexadecanoic acid	5283001	
	Tetrahydropyran-2-yl ether of 7-dodecynol	86051	
	9,10-Methylene-9-octadecenoic acid	12921	
	Stigmastan-3,5-diene	525918	
	Sitosterol	222284	
54. *Coptis chinensis*	Acetone diethyl dithioacetal	525464	[119]
	Hexanal dimethyl acetal	74137	
	5-Methyl furfural	12097	
	Cyclopentenone	13588	
	1-Acetoxyacetone	11593	
	2,3-Pentanedione	11747	
	1-Propionylethyl acetate	537235	
	1-Acetoxy-2-butanone	15302	
	3-Methyl-2-cyclopenten-1-one	17691	
	2,3-Dimethyl-2-cyclopenten-1-one	14270	
	2,2-Dimethyl-3(2H)-furanone	147604	
	3-Ethyl-2-hydroxy-2-cyclopenten-1-one	62752	
	4,5-Dimethyl-4-hexen-3-one	519408	
	2-Hydroxycyclopent-2-en-1-one	82674	
	Methyl cyclopentenolone	6660	
	(E)-3,4-Dimethyl-3-hexen-2-one	5363236	
	Maltol	8369	
	Acetosyringone	17198	
	1-(2-Hydroxy-5-methoxyphenyl)ethanone	69714	
	3-Butyl-2-methylenecyclohexanone	348533261	
	Vanillyl methyl ketone	17262	
55.*Cornus officinalis*	Glycidol	11164	[120]
	Acetone	180	
	Formic acid	284	
	1,3-Cyclohexadiene	11605	
	Methacrolein	6562	
	Acetic acid, cyclohexyl ester	12146	
	1,2-Ethanediol	174	
	Propanoic acid	1032	

	2-Vinylfuran	73881	
	Propanoic acid, 2-oxo-, methyl ester	11748	
	1-Ethoxypropan-2-yl acetate	171378	
	3-Pyrrolidinol	98210	
	Furfural	7362	
	2-Cyclopenten-1-one	13588	
	4-Cyclopentene-1,3-dione	70258	
	2-Furanmethanol	7361	
	4-Cyclopentene-1,3-dione	70258	
	1,3,5,7-Cyclooctatetraene	637866	
	2-Cyclohexen-1-ol	13198	
	2-Cyclohexen-1-one	13594	
56. *Croton macrostachyus*	Lupeol	259846	[121]
	Crotepoxide	161314	
	Methyl laurate	8139	
	Trachyloban-19-oic acid	44560009	
	Trachyloban-18-oic acid	101282656	
57. *Curcuma longa*	Benzeneacetaldehyde	998	[122]
	3-Hydroxy-.beta.-damascone	5366075	
	n-Hexadecanoic acid	985	
	Hexadecanoic acid, ethyl ester	12366	
	Phytol	5280435	
	9,12,15-Octadecatrienoic acid, methyl ester, (Z,Z,Z)-	5319706	
	Cyclododecyne	136909	
	Z-6,17-Octadecadien-1-ol acetate	5363378	
	9,12,15-Octadecatrienoic acid, ethyl ester, (Z,Z,Z)-	5367460	
	2-Ethylacridine	610161	
	4H-Pyran-4-one, 2,3-dihydro-3,5-dihydroxy-6-methyl-	119838	
	Hexadecanoic acid, ethyl ester	12366	
	9-Octadecenamide, (Z)-	5283387	
	9,12-Octadecadienoic acid (Z,Z)-	5280450	
	9,12-Octadecadienoic acid, ethyl ester	5365672	
	2,3,5-Trimethylfuran	517859	
	Elixene	94254	
	Tumerone	558173	
	Acoradiene	90351	
	Dicumyl peroxide	6641	
	Benzeneacetaldehyde	998	[123]

58. *Cyamopsis tetragonoloba*	3-Hydroxy-.beta.-damascone	5366075	
	n-Hexadecanoic acid	985	
	Hexadecanoic acid, ethyl ester	12366	
	Phytol	5280435	
	9,12,15-Octadecatrienoic acid, methyl ester, (Z,Z,Z)-	5319706	
	Cyclododecyne	136909	
	Z-6,17-Octadecadien-1-ol acetate	5363378	
	9,12,15-Octadecatrienoic acid, ethyl ester, (Z,Z,Z)-	5367460	
	2-Ethylacridine	610161	
	4H-Pyran-4-one, 2,3-dihydro-3,5-dihydroxy-6-methyl-	119838	
	Hexadecanoic acid, ethyl ester	12366	
	9-Octadecenamide, (Z)-	5283387	
	9,12-Octadecadienoic acid (Z,Z)-	5280450	
	9,12-Octadecadienoic acid, ethyl ester	5365672	
59. *Cynara scolymus*	Benzeneacetaldehyde	998	[124]
	3-Hydroxy-.beta.-damascone	5366075	
	n-Hexadecanoic acid	985	
	Hexadecanoic acid, ethyl ester	12366	
	Phytol	5280435	
	9,12,15-Octadecatrienoic acid, methyl ester, (Z,Z,Z)-	5319706	
	Cyclododecyne	136909	
	Z-6,17-Octadecadien-1-ol acetate	5363378	
	9,12,15-Octadecatrienoic acid, ethyl ester, (Z,Z,Z)-	5367460	
	2-Ethylacridine	610161	
	4H-Pyran-4-one, 2,3-dihydro-3,5-dihydroxy-6-methyl-	119838	
	Hexadecanoic acid, ethyl ester	12366	
	9-Octadecenamide, (Z)-	5283387	
	9,12-Octadecadienoic acid (Z,Z)-	5280450	
	9,12-Octadecadienoic acid, ethyl ester	5365672	
60. *Dalbergia sissoo*	Undecane	14257	[125]
	Dodecane	8182	
	Tridecane	12388	
	Tetradecane	12389	
	Pentadecane	12391	
	Hexadecane	11006	

	Heptadecane	12398	
	Octadecane	11635	
	Nonadecane	12401	
	Eicosane	8222	
	Heneicosane	12403	
	Docosane	12405	
	Tricosane	12534	
	Tetracosane	12592	
	Pentacosane	12406	
	Hexacosane	12407	
	Heptacosane	11636	
	Octacosane	12408	
	Nonacosane	12409	
	Triacontane	12535	
	Untriacontane	12410	
	Tritriacontane	12411	
	Lupenone	92158	
61. *Datura stramonium*	Hygrine	440933	[126]
	6,7-Dehydrotropine	564940	
	Cyclotropine	86063784	
	Tropinone	79038	
	Tropine	8424	
	Pseudotropine	449293	
	Scopoline	261184	
	Scopine	1274465	
	3-Acetoxytropane	103008	
	3,6-Dihydroxytropane	68871695	
	Methylecgonine	251884	
	3-(Hydroxyacetoxy)tropane	91750085	
	3-Acetoxy-6-hydroxytropane	565059	
	3-Hydroxy-6-acetoxytropane	14413733	
	3,7-Dihydroxy-6-propionyloxytropane	129846306	
	6,7-Dehydro-3-tigloyloxytropane	129846299	
	3,6-Diacetoxytropane	129846182	
	3-Hydroxy-6-isobutyryloxytropane	91750084	
	3-Tigloyloxy-6,7-epoxytropane	91700481	
	3-Tigloyloxy-6-hydroxytropane	91746695	
	3-Tigloyloxy-6-acetoxytropane	129662795	
	3-Tigloyloxy-6-propionyloxy-7-hydroxytropane	91700480	
	3-Phenylacetoxytropane	11086474	
	Aponorscopolamine	250155805	
	Littorine	443005	
62. *Emblica phyllanthus*	Citronellyl propionate	8834	[127]
	1-Methyl -4 isopropyl-cyclohexyl 2-hydroperfluorobutanoate	565481	
	Citronellyl acetate	9017	
	Hexadecanoic acid, methyl ester	8181	

	Pentadecanoic acid, 14- methyl, methyl ester	21205	
	Capric acid methyl ester	8050	
	Hexacontanoic acid	545975	
	N-Hexatriacontane	12412	
	N-Dotriacontane	11008	
	Stigmast-5-en-3-ol,oleate	20831071	
	N-Dotriacontane	11008	
	N-Hexacosane	12407	
	N-Tetracosane	12592	
	Oleyl alcohol	5284499	
	Stigmast-5-en-3-ol,oleate	20831071	
	Lanost-8-ene	22296726	
	Manganese,.pi.-cyclohexadienyl(hexamethylbenzene)	51052656	
	8-Amino-5-benzyloxy-6-methoxy-4-methylquinoline	609005	
	Ergost -5-en-3-ol,acetate,(3,beta.,24R)-	13019955	
63. *Eriobotrya japonica*	Hexanal	6184	[128]
	Undecane	14257	
	Dodecane	8182	
	Butyl butyrate	7983	
	(3E)-3-Hexenal	643139	
	2-Pentylfuran	19602	
	Ethyl caproate	31265	
	Styrene	7501	
	Hexyl acetate	8908	
	Tridecane	12388	
	1-Hexanol	8103	
	Tetradecane	12389	
	Hexyl butyrate	17525	
	Ethyl caprylate	7799	
	Benzaldehyde	240	
	Acetophenone	7410	
	Ethyl benzoate	7165	
	Naphthalene	931	
	Methyl salicylate	4133	
	1-Phenyl-1,2-propanedione	11363	
	Benzyl alcohol	244	
	2-Phenylethanol	6054	
	Biphenyl	7095	
	Cinnamaldehyde	637511	
	Mandelic acid	1292	
	Benzoic acid	243	
64. *Limonia acidissima*	(3-Methyl-oxiran-2-yl)-methanol	234485	[129]
	Ethylbenzene	7500	
	Hexadecanoic acid, methyl ester	8181	

	n-Hexadecanoic acid	985	
	11,14-Eicosadienoic acid	5282805	
	9-Octadecenoic acid (Z)-, methyl ester	5364509	
	Oleic acid	445639	
	Octadecanoic acid	5281	
	Stigmasterol	5280794	
	Bis(2-ethylhexyl) phthalate	8343	
	Tris(2,4-di-tert-butylphenyl) phosphate	14572930	
	N-Decanoylmorpholine	94801	
	Cholest-4-en-3-ol	227042	
	Stigmasterone	14807783	
	Lanosterol	246983	
	Lupeol	259846	

65. Bunium persicum	α-Thujene	17868	[130]
	α-Pinene	6654	
	Sabinene	18818	
	β-Pinene	440967	
	α-Terpinene	7462	
	P-Cymene	10908223	
	Limonene	22311	
	1, 8-Cineol	46781028	
	γ-Terpinene	7461	
	Terpinolene	11463	
	Linalool	6549	
	Terpinene-4-ol	11230	
	P-Cymen-8-ol	14529	
	Cuminaldehyde	326	
	Thymol	6989	
	α-Methyl-benzene methanol	10732586	
	Pinocarvyl acetate	102553	
	Geranyl acetate	1549026	
	Cuminyl acetate	100990	
	γ-Elemene	12309452	
	Caryophyllene	5281515	
	α-Selinene	10856614	
	β-Bisabolene	10104370	
	Croweacin	5316141	
	Caryophyllene oxide	1742210	
	Spathulenol	92231	
	Dillapiole	10231	
	α-Bisabolol	442343	
	α- Thujene	12444324	

			[131]
	α- Pinene	6654	
	Sabinene	18818	
	β -Pinene	14896	
	Myrcene	31253	
	ρ -Cymene	7463	
	α-Terpinene	7462	
	σ-Cymene	7463	
	Limonene	22311	
	γ- Terpinene	7461	
	Terpinene-4-ol	11230	
	α- Terpineol	17100	
	Cuminaldehyde	326	
	Cuminyl alcohol	325	
	β- Caryophyllene	1742210	
	γ-Eleman	11107211	
	β- Bisabolene	10856614	
	β- Selinene	10856614	
	Myristicin	4276	
	Germacrene B	5281519	
	Beta Dillapiol	377681	
	γ-Terpinene	7461	[132]
	Limonene	22311	
	p-Cymene	7463	
	β-Pinene	14896	
	α-Pinene	6654	
	Cuminaldehyde	326	
	Myrcene	31253	
	**α -Thujene**	12444324	

	**α -Pinene**	6654	[133]
	**Camphene**	6616	
	**Sabinene**	18818	
	**ß-Pinene**	14896	
	**Myrcene**	31253	
	**δ -2-Carene**	78249	
	**Isosylvestrene**	10354	
	**ρ -Cymene**	7463	
	**Limonene**	22311	
	**1,8-Cineole**	2758	
	**Z-ß-Ocimene**	5320250	
	**γ -Terpinene**	7461	
	**3-methylbenzaldehyde**	12105	
	**cis-Sabinene hydrate**	62367	
	**Terpinolene**	11463	
	**Linalool**	6549	
	**trans-Sabinene hydrate**	12315151	
	**Borneol**	64685	
	**Terpinen-4-ol**	11230	
	**α -Terpineol**	17100	
	**Perillaldehyde**	16441	
	**Bornyl acetate**	93009	
	**α -Terpinen-7-al**	526762	
	**Thymol**	6989	
	**ar-Curcumene**	92139	
	**Germacrene D**	5317570	
	**α -Zingiberene**	92776	
	**ß-Sesquiphellandrene**	519764	
66. Caesalpinia decapetala	α-pinene	6654	[134]

	β-phellandrene	11142	
	Camphene	6616	
	β-pinene	440967	
	(Z)-hex-3-enyl acetate	5363388	
	β-Myrcene	31253	
	α-phellandrene	7460	
	α-thujene	17868	
	α-pinene	6654	
	β-pinene	440967	
	β-myrcene	31253	
	δ-3-carene	26049	
	p-cymene	7463	
	limonene	22311	
	(Z)-β-ocimene	5320250	
	phenyl acetaldehyde	998	
	(E)-β-ocimene	5281553	
	linalool	6549	
	nonanal	31289	
	trans-mentha-2,8-dien-1-ol	155626	
	trans -limonene oxide	449290	

	verbenol	61126	
	p-mentha-1,5-dien-8-ol	519323	
	cis-linalool oxide	6428573	
	trans-linalool oxide	6432254	
	α-terpineol	17100	
	methyl salicylate	4133	
	verbenone	29025	
	trans-carveol	94221	
	geraniol	637566	
	δ-elemene	12309449	
	α-cubebene	442359	
	α-copaene	12303902	
	geranyl acetate	1549026	
	β-bourbonene	62566	
	β-elemene	6918391	
	β-caryophyllene	5281515	
	β-copaene	21722369	
	trans-muurola-3,5-diene	10632031	
	α-humulene	5281520	
		91354	

	aromadendrene		
	bicyclogermacrene	13894537	[135]
	bicyclogermacrene	13894537	
	α-muurolene	12306047	
	(E,E)-α-farnesene	5281516	
	γ-cadinene	6432404	
	δ-cadinene	6432404	
	trans-cadina-1,4-diene	6430869	
	α-cadinene	12306048	
	(E)-nerolidol	5284507	
	spathulenol	92231	
	caryophyllene oxide	1742210	
	viridiflorol	11996452	
	khusimone	6428327	
	humulene epoxide	5352470	
	junenol	6452077	
	1-epi-cubenol	519857	
	epi-α-muurolol	3084331	
	α-muurolol<br>α-cadinol	3084331<br>10398656	

	14-hydroxy-9-epi-β-caryophyllene	12300118	
	(2E, 6Z)-farnesol	1551481	
	methyl palmitate	8181	
	palmitic acid	985	
	sandaracopimarinal	620819	
	tricosane	12534	
	pentacosane	12406	
	heptacosane	11636	
	nonacosane	12409	
	monoterpene	10282	
	sesquiterpene hydrocarbons	6473767	
	sesquiterpene alcohols	481177769	
67. Calendula officinalis	Propane, 1,1,2,3,3- pentachloro	27027	[136]
	1H-Indole-2,3-dione, 5- heptyl-1-(trimethylsilyl)-, 3-(O-methyloxime)	9604164	
	1-Propanone, 1-(1- cyclohexenyl)-3-(8- quinolinylamino)-	5301329	
	.tau.-Muurolol	3084331	
	.alpha.-Cadinol	6431302	
	1H-3a,7-Methanoazulen-6- ol, octahydro-3,6,8,8-tetramethyl-, acetate, [3R- (3.alpha.,3a.beta.,6.alpha.,7 .beta.,8a.alpha.)]-	162002	
	Cyclodecasiloxane, eicosamethyl	519601	

	l-(+)-Ascorbic acid 2,6- dihexadecanoate	54722209	
	Stigmasta-4,22-diene	91697635	
	9,12-Octadecadienoic acid (Z,Z)-	5284421	
	Octadec-9-enoic acid	965	
	2,6-Dichlorobenzyl ether	52735	
	Octadecanoic acid	5281	
	.alpha.-D-Ribofuranoside phenyl-2,3-Oisopropylidene-1-thio	91726189	
	Methyl 2,4,6-trichloro-3,5- dimethoxybenzoate	533419	
	Quinuclidine-2-carboxylic acid, 2,3-dehydro-3-amino- , ethyl ester	534367	
	9-Octadecenoic acid, 1,2,3- propanetriyl ester, (E,E,E)-	5364673	
	Hinesol	289964	
	11- Oxatetracyclo[4.2.1.1(2,5).1(7,10)]undec-3-ene, 9-methoxy-9-methyl	605926	
	11-Dodecyn-1-ol acetate	538082	
	Acetoxyacetic acid, undec-2-enyl ester	605926	
	Benz[d]oxatiolane-2- thione, 7-(4- chlorophenyl)-5-hydroxy	626386	
	Pyridine, 2,6-diamino-3- ((2,5-dichloropenyl)azo)	97206	
	4-[1-Adamantyl]-3- thiosemicarbazone 4-bromoacetophenone	9603018	
	3,6-Dodecadienoic acid, methyl ester	5367408	

	2,4-Dimethyl- nonanedioic acid<br>3-(3,4-Dichlorophenyl)-1- (5-methyl-2-thiazolin-2- yl)-1-(3,4-xylyl)urea	560311	
	7,9-Di-tert-butyl-1- oxaspiro(4,5)deca-6,9- diene-2,8-dione	545303	
	Hexadecanoic acid, methyl ester	8181	
	l-(+)-Ascorbic acid 2,6- dihexadecanoate	54722209	
	Tetracyclo[6.6.1.0(2,7).0(9,14)]pentadeca-4,11- diene	556732	
	E,Z-1,3,12- Nonadecatriene	5365680	
	2,3-Nonadecanediol	566291	
	9,12-Octadecadienoic acid (Z,Z)-	5280450	
	6-Octadecenoic acid	5282754	
	1-(4-Amino-furazan-3- yl)-5-methoxymethyl-1H-[1,2,3]triazole-4- carboxylic acid	1201476	
	Octadecanoic acid	5281	
	1,5,7-Octatrien-3-ol, 2,6-dimethyl-	5362854	
	Cyclohexane, azido	88153	
	4H-Pyran-4-one, 2,3- dihydro-3,5-dihydroxy-6- methyl	119838	
	3-Cyclohexene-1- methanol, .alpha.,.alpha.,4- trimethyl-, acetate	111037	
	Ethyl (E)-2-octenoate	5364399	
	Benzaldehyde, 2- hydroxy-6-methyl-	64982856	
	1-Decyloxy-2- nitrobenzene	25058971	

	Pentanoic acid, 5- hydroxy-, 2,4-di-tbutylphenyl esters	605777	
	1H-Pyrrole, 2-(2,4,6- cycloheptatrienyl)-	593678	
	.tau.-Cadinol	6429185	
	.alpha.-Cadinol	6431302	
	Phenol, 4-propoxy	29352	
	Perfluoroheptyl iodide	67633	
	8-Acetoxymethyl-3-fluoroadenine	539375	
	Androst-4-ene-3,11-dione	22216238	
	3-Hydroxy-2,3,4,4-tetramethyl-pentanoic acid, ethyl ester	567503	
	D-Erythro-Hex-2-enonic acid, 2,3-di-O-methyl-5,6-bis-O-(trimethylsilyl)-, .gamma.- lactone	90473664	
	Nicotinic acid, 2-ethyl-1,6-dihydro-4-hydroxy-6-oxo-, ethyl ester	54697839	
	o-(2-Benzylidenehydrazino)benzoic acid	9561044	
	l-(+)-Ascorbic acid 2,6-dihexadecanoate	54722209	
	9,12-Octadecadienoic acid (Z,Z)-	5280450	
	6-Octadecenoic acid	5282754	
	Octadecanoic acid, 2-(2-hydroxyethoxy)ethyl ester	7788	
	N-[1-(2,4-Difluoroanilino)-2,2,2-trifluoro-1-(trifluoromethyl)ethyl]isovaleramide	546400	
	Methyl 15-hydroxy-9,12-octadecadienoate	91692548	
	9-Octadecenoic acid, 1,2,3-propanetriyl ester, (E,E,E)-	5364673	

	Phthalic acid, 6-ethyloct-3-yl 2-ethylhexyl ester	6423901	
	Ethyl (E)-2-octenoate	5364399	
	Benzaldehyde, 2-hydroxy-6-methyl-	585174	
	1-Decyloxy-2-nitrobenzene	545677	
	Pentanoic acid, 5-hydroxy-, 2,4-di-t-butylphenyl esters	605777	
	1H-Pyrrole, 2-(2,4,6-cycloheptatrienyl)-	593678	
	syn-Tricyclo[5.1.0.0(2,4)]oct-5-ene, 3,3,5,6,8,8-hexamethyl-	600264	
	.tau.-Cadinol	6429185	
	.alpha.-Cadinol	10398656	
	Phenol, 4-propoxy-	29352	
	2,4-Dimethyl-nonanedioic acid, dimethyl ester	560311	
	3-(3,4-Dichlorophenyl)-1-(5-methyl-2-thiazolin-2-yl)-1-(3,4-xylyl)urea	613034	
	7,9-Di-tert-butyl-1-oxaspiro(4,5)deca-6,9-diene-2,8-dione	545303	
	Hexadecanoic acid, methyl ester	8181	
	l-(+)-Ascorbic acid 2,6-dihexadecanoate	54722209	
	Boron, di-1,5-cyclooctanediyl[.mu.-(3,5-dimethyl-1H-pyrazolato-N1:N2)]-.mu.-hydrodi-	250085223	
	Tetracyclo[6.6.1.0(2,7).0(9,14)]pentadeca-4,11-diene	556732	
	E,Z-1,3,12-Nonadecatriene	5365680	
	2,3-Nonadecanediol	566291	

	9,12-Octadecadienoic acid (Z,Z)-	5280450	
	6-Octadecenoic acid	1201468	
	1-(4-Amino-furazan-3-yl)-5-methoxymethyl-1H-[1,2,3]triazole-4-carboxylic acid	1201468	
	Octadecanoic acid	5281	
	Z,Z-11,13-Hexadecadien-1-ol acetate	5363385	
	Thiophene, 2,5-dibromo-	18453	
	3,6-Dodecadienoic acid, methyl ester	5367408	
	4-[1-Adamantyl]-3-thiosemicarbazone 4-bromoacetophenone	9603018	
	Pyridine, 2,6-diamino-3-((2,5-dichloropenyl)azo)-	97206	
	Benz[d]oxatiolane-2-thione, 7-(4-chlorophenyl)-5-hydroxy-	626386	
	Acetoxyacetic acid, undec-2-enyl ester	5353110	
	11-Dodecyn-1-ol acetate	538082	
	9-Octadecenoic acid, 1,2,3-propanetriyl ester, (E,E,E)-	5364673	
	Hinesol	10878761	
	Acetyl eugenol	7136	[137]
	Phenol-4-octyl	15730	
	Guaiol	227829	
	Cedrol	65575	

	Octadecane	11635	
	Tetradecanoic acid	11005	
	Nonadecane	12401	
	Eicosane	8222	
	Heneicosane<br>Docosane	12403<br>12405	
	Tricosane	12534	
	Tetracosane	12592	
	Pentacosane	12406	
	Hexacosane	12407	
	Heptacosane	11636	
	Octacosane	12408	
	Eicosane-7-hexyl	292289	
	Eicosane-9-octyl	280905	
	Canescegenine	12308767	
	Cholest-4-en-3-one-14-methyl	277841	
	Taraxasterol	115250	
	1-Octadecanol	8221	
	1,16-Hexadecanediol	82184	
	Alloaromadendrene	10899740	[138]

	b-selinene	519361	
	Germacrene D	5317570	
	a-Cubebene	442359	
	a-muurolene	12306047	
	g-Cadinene	92313	
	d-Cadinene	441005	
	Cadina-1,4-diene	6427091	
	a-cadinene	12306048	
	Nerolidol	5284507	
	Palustrol	110745	
	Calarene	28481	
	Oplopenone	6429350	
	α-Pinene	6654	
	6-Methyl-5-heptene-2-one	9862	
	2-Pentylfuran	19602	
	α-Terpinene	7462	
	p-Cymene	7463	
	Limonene	22311	
	1,8-Cineol	2758	

	Benzeneacetaldehyde	998	
	γ-Terpinene	7461	
	4-Methylbenzaldehyde	7725	
	Terpinolene	11463	
	Linalool	6549	
	n-Nonanal	31289	
	2,6-Dimethylcyclohexanol	21428	
	cis-p-Mentha-2-en-1-ol	13918681	
	Camphor	2537	
	Terpinen-4-ol	11230	
	Safranal	61041	
	n-Decanal	8175	
	β-Cyclocitral	9895	
	Carvone	7439	
	Dihydrojasmone	62378	
	p-Thymol	18597	
	(E,E)-2,4-Decadienal	5283349	
	α-Cubebene	442359	
	α-Copaene	19725	
	β-Cubebene	93081	

	trans-β-Caryophyllene	5281515	
	α-Ionone	5282108	
	Bicyclosesquiphellandrene	521496	
	α-Humulene	5281520	
	Eudesma-4(14),7(11)-diene	6432497	
	γ-Muurolene	12313020	
	Epizonarene	595385	
	β-Ionone	638014	
	α-Elemene	80048	
	Ledene	10910653	
	α-Muurolene	12306047	
	δ-Cadinene<br>α-Terpineol	441005	
	Calamenene	6429077	
	Cadina-1,4-diene	6427091	
	α-Cadinene	12306048	
	Cadala-1(10),3,8-triene	593889	
	Elemol	92138	
	Longifolenaldehyde	565584	
	Spathulenol	92231	

	Caryophyllene oxide	1742210	
	Germacrene D-4-ol	5352847	
	Viridiflorol	11996452	
	Selina-6-en-4-ol	527220	
	10-Epi-γ-eudesmol	6430754	
	Ledol	92812	
	Carotol	442347	
	τ-Muurolol	3084331	
	δ-Cadinol	3084311	
	α-Cadinol	10398656	
	n-Heptadecane	12398	
	Caryophyllene alcohol,	61125	
	Myristic acid	11005	
	Palmitic acid	985	
	n-Heneicosane	12403	
	α-Pinene	6654	
	γ-Muurolene	12313020	
	α-Muurolene	12306047	
	γ-Cadinene	6432404	
	α-Ionone	5282108	

	β-Ionone	638014	
	Germacrene	9548705	
	Viridiflorol	11996452	
	Ledol	92812	
	Carotol	442347	
	δ-Cadinol	3084311	
	p-Thymol	18597	
	α-Cadinol	6431302	
	γ-Cadinene	6432404	
	a-Thujene (M)	520384	[139]
	a-Pinene (M)	6654	
	Sabinene (M)	18818	
	b-Pinene (M)	14896	
	b-Myrcene (M)	31253	
	a-Phellandrene (M)	7460	
	Estragole (O)	8815	
	Tridecane (H)	12388	
	6,7-Dimethyl 1,2,3,5,8,	570683	
	8a-hexahydronaphthalene (S)	101708	
	a-Copaene (S)	442359	

	b-Bourbonene (S)	62566	
	b-Cubebene (S)	93081	
	b-Caryophyllene (S)	5281515	
	cis-Muurola-3,5-diene	51351708	
	trans-Muurola-3,5-diene	10632031	
	a-Caryophyllene (S)	5281520	
	cis-Muurola-4(14),5-diene	51351709	
	trans-Cadina-1(6),4-diene (S)	10798255	
	Germacrene D (S)	5317570	
	b-Selinene (S)	519361	
	cis-Cadina-1,4-diene (S)	6431126	
	Bicyclogermacrene	13894537	
	a-Muurolene (S)	101708	
	d-Cadinene (S)	441005	
	trans-Cadina-1,4-diene (S)	6430869	
	a-Cadinene (S)	12306048	
	Caryophyllene oxide (SO)	1742210	
	(3R)-3-phenyl-2,3-dihydro-1H-isoindol-1-one	12158069	[140]
	ethyl 2-methyl butanoate	24020	

	ethyl β-methylvalerate	560255	
	1-octene-3-ol	18827	
	L-fenchone	82229	
	L-linalool	443158	
	(-)-camphor	444294	
	α-terpineol	17100	
	ethyloctanoate	7799	
	(-)-myrtenol	88301	
	p-allylanisole	8815	
	ethyloctanoate	7799	
	α-terpinenyl acetate	111037	
	eugenol	3314	
	2,6,6-trimethyl-2-hydroxycyclohexylidene)acetic, acid lactone	27209	
	ethyl palmitate	12366	
	9-(methylthio)-8H-acenaphtho[1,2-c]pyrrole-7-carboxylic acid	11346545	
	eucalyptol	2758	
	α-thujone	261491	
	β-thujone	49742744	

	1-borneol	129011821	
	4-terpineol	11230	
	(-)-bornyl acetate	93009	
	2-methoxy-3-methylhydroquinone	3787218	
	1,1-dimethoxycyclobutane	13049977	
	n-hexanol	8103	
	phenol	996	
	phenyl acetaldehyde	998	
	α-terpinolen	11463	
	(-)-camphor	444294	
	α-terpineol	17100	
	2-isopropyl-2,5-dihydrofuran	15128402	
	ethyl o-hydroxybenzoate	8365	
	(+)-ledene	10910653	
	δ-cadinene	92313	
	methyl-4-hydroxy-4-methyl-2-pentynoate	4277621	
	N,N-dimethyltryptamine	6089	
	ethyl (2E)-3-(4-hydroxy-3-methoxyphenyl)-2-propenoate	736681	
	α-Pinene	6654	

	Sabinene	18818	
	Myrcene	31253	
	α-phellandrene	11142	
	Limonene	22311	
	(Z)-(β)-ocimene	5320250	
	(E)-(β)-ocimene	18756	
	Dihydrotagetone	102706	
	Terpinolene	11463	
	Linalool	6549	
	P-mentha-1,3,8-triene	176983	
	Allo-ocimene	5368821	
	(E)-tagetone	5368938	[141]
	(Z)-tagetone	5368956	
	Borneol	64685	
	Terpinen-4-ol	11230	
	p-cymen-8-ol	14529	
	α- terpineol	17100	
	(Z)-ocimenone<br>(E)-ocimenone	6428432<br>6428431	
	Piperitone	6987	
	Thymol	6989	

	Piperitenone	381152	
	Piperitenone oxide	442497	
	β-caryophyllene	5281515	
	Germacrene-D	91723653	
	Bicyclogermacrene	13894537	
	(E)-β-farnesene/(E)-E-farnesene	5281516	
	(E)-nerolidol	5284507	
	Spathulenol	92231	
	Caryophyllene oxide	1742210	
	2,4-Di-tert-butylphenol	7311	[142]
	Hexadecane	11006	
	Adipic acid	196	
	Di-epi-alpha-cedrene	10878276	
	Zaragozic acid	6438355	
	Di-n-decylsulfone	19089489	
	Docosane	12405	
	Hentriacontane	12410	
	Pentadecane	12391	
	Nonadecane	12401	
	n-Tetracosanol-1	10472	

	Propanediol	134919	
	Isopropyl myristate	8042	
	Lauric acid	3893	
68. Camellia sinensis	Lactic acid (LA), 2TMS	612	[143]
	L-Alanine (Ala), 2TMS	5950	
	Oxalic acid (OA), 2TMS	971	
	Hydroxylamine (HX), 3TMS	787	
	L-Valine (Val), 2TMS	6287	
	L-Leucine (Leu), 2TMS	6106	
	Silanol, trimethyl-phosphate(3:1) (Sil)	25317	
	L-Isoleucine (Isol), 2TMS	6306	
	Butanedioic acid (BA), 2TMS	1110	
	Serine (Ser), 3TMS	5951	
	L-Threonine (Thr), 3TMS	6288	
	Malic acid (MA), 3TMS	525	
	L-5-Oxoproline (Oxo), 2TMS	7405	
	L-Aspartic acid (Asp), 3TMS	5960	
	4-Aminobutanoic acid (AA), 3TMS	119	
	2,3,4-Trihydroxybutyric acid tetrakis (TAT)	439535	
	L-Glutamic acid (LGA), 3TMS	33032	

	L-Glutamine (Glut), 3TMS	5961	
	2-keto-L-gluconic acid (KLGA)	10198044	
	Shikimic acid (SA), 4TMS	8742	
	Citric acid (CA), 4TMS	311	
	Quinic acid (QA), 5TMS	6508	
	D-Glucose (Glu)	5793	
	Myristic acid (MyA), TMS	11005	
	Oleanitrile (ON)	6420241	
	Myo-Inositol (Myo), 6TMS	892	
	Oleamide (OM), TMS	5283387	
	Sucrose (Suc), 8TMS	5988	
	Linalool	6549	[144]
	Benzyl acetate	8785	
	β-Ionone	638014	
	Methyl salicylate	4133	
	Olivetol	10377	
	Geraniol	637566	
	Phenethyl alcohol	6054	
	Nerolidol	5284507	
	β-Cyclocitral	9895	

	(2,6,6-Trimethyl-2-hydroxycyclohexylidene) acetic acid lactone	27209	
	Benzaldehyde	240	
	7,11,15-Trimethyl-3-methylidene-hexadec-1-ene	10446	
	Farnesene	5281516	
	Geranylacetone	1549778	
	Delta-cadinene	441005	
	cis-Hex-3-enyl hexanoate	61895	
	Jasmone	1549018	
	Indole	798	
	2,3-Dihydro-2,2,6-trimethylbenzaldehyde	61041	
	3,5-Octadien-2-one	5352876	
	trans,trans-2,4-Heptadienal	5283321	
	Linalool oxide II	6432254	
	(–)-Alpha-terpineol	6432254	
	2-Acetyl pyrrole	14079	
	Cedrol	65575	
	cis-3-Hexenyl acetate	5363388	
	(E,E)3, 5-Octadien-2-one	5352876	
	Linalool oxide I	6428573	

	Benzyl alcohol	244	
	1-octen-3-ol	18827	
	1-Nonanal<br>Hexyl hexanoate	31289<br>22873	
	6-Methyl-5-hepten-2-one	9862	
	(E)-3,7-Dimethylocta-1, 3, 6-triene	5281553	
	2-Pentanone, 4-hydroxy-4-methyl-	618244	[145]
	2′,6′Dihydroxyacetophenone, bis(trimethylsilyl) ether	91740707	
	n-Heptadecanol-1	15076	
	1-Hexadecanol	2682	
	Heptane, 1-bromo-6-methyl	104270	
	2-Pentanone, 4-hydroxy4methyl		
	2′,6′Dihydroxyacetophenone, bis(trimethylsilyl) ether	91740707	
	N(Trifluoracetyl)O,O′,O″tris(trimethylsilyl)epinephrine	553891	
	Tetracosamethyl cyclododecasiloxane	167767	
	Benzoic acid, 2(dimethylamino)ethyl ester	75158	
	Hexasiloxane, tetradecamethyl	7875	
	Heptasiloxane, hexadecamethyl<br>4-Hydroxyphenylacetic acid, ethyl ester<br>616985	10912	
	1-Octen-3-ol	18827	[146]
	Myrcene	31253	

	Limonene	22311	
	Ocimene	5281553	
	Linalool	6549	
	Nonanal	31289	
	Phenylethyl alcohol	6054	
	Linalool oxide (pyranoid)	6432254	
	Naphthalene	931	
	Methyl salicylate	4133	
	Safranal	61041	
	Dodecane	8182	
	Decanal	8175	
	Geraniol Hexanoic	5365992	
	2-Methyl naphthalene	7055	
	α-Cubebene	442359	
	α-Ionene	68057	
	Jasmone	1549018	
	Tetradecane	12389	
	Longifolene	289151	
	Cedrene	521207	
	β-Caryophyllene	5281515	
	α-Ionone	5282108	

	Geranyl acetone	1549778	
	β-Farnesene	5281517	
	Alkane	8182	
	γ-Muurolene	12313020	
	α-Curcumene	92139	
	β-Ionone	638014	
	Pentadecane	12391	
	α-Farnesene	5281516	
	γ-Cadinene	92313	
	δ-Cadinene	441005	
	Nerolidol	5284507	
	1-Hexadecene	12395	
	Hexadecane	11006	
	Heptadecane	12398	
	Octadecane	11635	
	Caffeine	2519	
	1-Pentanol	6276	[147]
	(Z)-3-Hexen-1-ol	5281167	
	Benzaldehyde	240	
	1-Octen-3-ol	18827	

	2-Pentyl-furan	19602	
	α-Phellandrene	7460	
	α-Terpinene	7462	
	D-Limonene	440917	
	Benzyl alcohol	244	
	(E)-3,7-Dimethyl-1,3,6-octatriene	5281553	
	Phenyl acetaldehyde	998	
	Ocimene	5281553	
	γ-Terpinene	7461	
	(E)-2-Octen-1-ol	5318599	
	Linalool oxide II	6432254	
	Linalool	6549	
	Hotrienol	5366264	
	Phenylethyl alcohol	6054	
	Benzene acetonitrile	8794	
	Linalool oxide III	6427787	
	Linalool oxide IV	6427788	
	Naphthalene	931	
	α-Terpineol	17100	
	Methyl salicylate	4133	

	Safranal<br>Dodecane<br>8182	61041	
	β-Cyclocitral	9895	
	Nerol	643820	
	Geraniol	637566	
	2-Methyl-naphthalene	7055	
	Indole	798	
	Tridecane	12388	
	1-Methyl-naphthalene	7002	
	Isopropyl salicylate	11838	
	1,2,3-Trimethoxybenzene	12462	
	2,6-Dimethoxyphenol	7041	
	Butanoic acid butyl ester	7983	
	(Z)-3-Hexenyl hexanoate	5352543	
	cis-Jasmone	1549018	
	Tetradecane	12389	
	6,10-Dimethyl-2-undecanone	95495	
	α-Cedrene	6431015	
	β-Caryophyllene<br><br>α-Ionone<br>5282108	5281515	

	1,2-Benzopyrone	1871	
	Geranyl acetone	1549778	
	(E)-β-Farnesene		
	β-Ionone	5282108	
	Pentadecane	12391	
	Dibenzofuran	568	
	α-Farnesene	5281516	
	Dihydroactinidiolide	27209	
	α-Calacorene	528708	
	Nerolidol	5284507	
	Fluorene	6853	
	Cedrol	65575	
	Hexadecane	11006	
	α-Cadinol	10398656	
	2,2′,5,5′-Tetramethyl-1,1′-biphenyl	14923757	
	2-Methyl-hexadecane	14923757	
	Heptadecane<br>2,6,10,14-Tetramethyl pentadecane<br><br>15979	12398	
	Anthracene	8418	
	Octadecane	11635	

	2,6,10,14-Tetramethyl hexadecane	12523	
	Isopropyl myristate	8042	
	Caffeine	2519	
	Phytone	10408	
	Farnesyl acetone	1711945	
	Hexadecanoic acid methyl ester	8181	
	Isophytol	10453	
	Hexadecanoic acid	985	
	Methyl linoleate	5284421	
	Phytol	5280435	
	1-Hexanol	8103	[148]
	1-(1-Cyclohexen-1-yl)ethanone	13612	
	2-Heptanone	8051	
	Heptanal	8130	
	2,5-Dimethylpyrazine	31252	
	Ethylpyrazine	31252	
	(E)-2-Heptenal	5283316	
	Benzaldehyde	240	
	5-Methyl-2-furaldehyde	12097	
	6,6-Dimethyl-undecane	28474	

	3,5,5-Trimethyl-2-hexene	5365055	
	1-Hepten-3-one	520420	
	1-Octen-3-ol	18827	
	2-Pentyl-furan	19602	
	(E,E)-2,4-Heptadienal	5283321	
	2-Ethyl-5-methylpyrazine	25915	
	Decane	15600	
	Octanal	454	
	α-Terpinene	7462	
	2-(2-Propenyl)-furan	530402	
	D-Limonene	440917	
	Benzyl alcohol	244	
	(E)-4-Oxohex-2-enal	6365145	
	3-Octen-2-one	5363229	
	Benzeneacetaldehyde	998	
	1-Ethyl-1H-pyrrole-2-carbaldehyde	579338	
	β-Ocimene	18756	
	(E)-2-Octenal	5283324	
	Acetophenone	7410	
	1-(1H-pyrrol-2-yl)ethanone	14079	

	1-Octanol	957	
	3-Ethyl-2,5-dimethylpyrazine	25916	
	2,6-Diethylpyrazine	83101	
	3,5-Octadien-2-one	5352876	
	Undecane	14257	
	Linalool oxide	102611	
	Nonanal	31289	
	Phenylethyl Alcohol	6054	
	1-Ethyl-2,5-pyrrolidinedione	16842	
	(E,E)-2,6-Nonadienal	636687	
	(E)-2-Nonenal	5283335	
	3-Methyl-undecane	521960	
	Methyl salicylate	4133	
	Dodecane	8182	
	Decanal	8175	
	β-Cyclocitral	9895	
	1-Phenyl-2-butanone	13879	
	n-Valeric acid cis-3-hexenyl ester	5367682	
	Geraniol	637566	
69. Capparis spinosa	9,12,15-Octadecatrienoic acid ,(Z,Z,Z)-	5280934	[149]

	18-Nonadecenoic acid	543857	
	3-Azabutyl-1-ol, 4-cyclopropyl-3,3-dimethyl bromide	544213	
	Methyl 6-oxoheptanoate	288022	
	Paromomycin	165580	
	5-Oxohexanethioic acid ,S-t-butyl ester	574740	
	1-Methyl-2-pyrrolidineethanol	93363	
	DL-Leucine , N-glycyl-	102468	
	4-Hexenal,6-hydroxy-4-methyl-,dimethyl acetal , acetate , (Z)	5366877	
	1-Methyl-pyrrolidine-2-carboxylic acid	557	
	Pyrrolizin-1,7-dione-6-carboxylic acid , methyl (ester)	558485	
	2-Cyclohexylpiperidine	92439	
	3,6-Diazahomoadamantan-9-one Hydrazone	547221	
	17-Octadecynoic acid	1449	
	n-Hexadecanoic acid	985	
	Diphenyl-1,6-dioxopyridazino [4,5:2',3']pyrrolo[4',5'—d]pyrico	135833395	
	D-Glucose , 6-O-α-D-galactopyranosyl-	11458	
	Dimethyl fumarate	637568	
	2-Pyridineacetic acid ,hexahydro-	3164325	
	DL-Leucine , N-glycyl-	102468	

	2H-Benzotriazole , 2-methyl-	85498	
	Acetate , [3-(acetyloxy)-4,5-dihydro-5-isoxazolyl]methyl		
	2,5-Octadecadiynoic acid , methyl ester	42151	
	9-Hexadecenoic acid	445638	
	Oxacyclododecan-2-one	74409	
	Spiro[2.4]heptan-4-one	258618	
	Isopropyl isothiocyanate	75263	[150]
	p-Cymene	7463	
	Methyl isothiocyanate	11167	
	Thujone	261491	
	Caryophyllene	5281515	
	Camphor	2537	
	Humulene	5281520	
	1,2,3,5,6,8a-Hexahydro-4,7-dimethyl-1-(1-methylethyl)-naphthalene	12306055	
	3-Cyclohexen-1-one	77727	
	3-Methyl-4-isopropylphenol	18597	
	Spathulenol	92231	
	Muurolol	3084311	
	6,10-Dimethyl-2-undecanone	95495	

	Tetracosane	12592	
	Eicosane	8222	
	Heptacosane	11636	
	1-Naphthalenepropanol	14711518	
	2-Cyclohexen-1-ol	13198	
	1,2-Benzenedicarboxylic acid	1017	
	Isobutyl 2-methylpent-3-yl ester	91701588	
	Phenanthrene	995	
	Dibutyl phthalate	3026	
	3-Penten-1-yne, 3-methyl-, (Z)	5367413	[151]
	Isopropyl alcohol	3776	
	Furfural	7362	
	Pyrrole, 4-ethyl-2-methyl	521768	
	5-Methylfurfural	12097	
	Phenol	996	
	Benzyl alcohol	244	
	Proline, N-methyl-, butyl ester	527835	
	Guaiacol	460	
	Benzoic acid	243	
	Coumaran	10329	

	1-Methoxycyclohexane	13607	
	2-Methylpiperidine	7974	
	Isonaline 70	5372174	
	Indole	798	
	Proline, N-methyl-, butyl ester	527835	
	5-Methoxy-1H-indole-3- carbaldehyde	82758	
	Phenol, 2-methoxy-4-(1-propenyl)	6437176	
	n-Hexadecanoic acid	985	
	Phytol	5280435	
	Nonacosane	12409	
	Hexadecane, 1-iodo	11292722	
	Octadecane, 1-iodo	12402	
	Hentriacontane	12410	
	Docosane	12405	
	Eicosane	8222	
	Tetratriacontane	26519	
	Beta-Sitosterol	222284	
	ButylatedHydroxytoluene	14863	
	Pyrrolidine, 1-methyl	8454	
	Pyrrolidine-5-one, 2-[3-hydroxypropyl]	558386	[152]

	1-Methyl-pyrrolidine-2-carboxylic acid	557	
	2-Methoxy-4-vinylphenol	332	
	Cholestan-3-amine, N,N,4,4-tetramethyl- , (3.beta.,5.alpha.)	22214748	
	Benzyl benzoate	2345	
	Neophytadiene	10446	
	Hexadecanoic acid, methyl ester	8181	
	Hexadecanoic acid	985	
	9,12,15-Octadecatrienoic acid, methyl ester, (Z,Z,Z)-	5319706	
	Phytol	5280435	
	9,12,15-Octadecatrien-1-ol, (Z,Z,Z)-	6436081	
	Adipic acid, bis(2-ethylhexyl) ester	7641	
	1H-Indole, 2-methyl-	7224	
	Butane, 2-phenyl-3-(trimethylsilyloxy)-	575922	
	1,1,1,3,5,5,5-Heptamethyltrisiloxane	6327366	
	Cyclotrisiloxane, hexamethyl-	10914	
	(23S)-ethylcholest-5-en-3.beta.-ol	91750025	
	Gibberellin A3	6466	
	Ethanol, 2,2-diethoxy-	12129	
	1,3-Disilacyclobutane, 1,1,3,3-tetramethyl-	137123	
	4,7-Methano-1H-inden-1-one,2,3,3a,4,7,7a-hexahydro-	261675	

	2-propyl cyclopentanone	40425580	
	DL-Proline, 5-oxo-, methyl ester	500249	
	Cholestan-3-amine, N,N,4,4-tetramethyl-,(3.beta.,5.alpha.)-	22214748	
	4-Hydroxy-3-methoxyphenyl) ethyl methyl ketone	31211	
	n-Hexadecanoic acid	985	
	Hexadecanoic acid, ethyl ester	12366	
	9,12,15-Octadecatrien-1-ol, (Z, Z, Z)-	6436081	
	9,12,15-Octadecatrienoic acid, ethyl ester,(Z, Z, Z)-	5367460	
	4,7-Methano-1H-inden-1-one,2,3,3a,4,7,7a-hexahydro-	261675	
	2-Methoxy-3-isopropylpyrazine	33166	
	n-Hexadecanoic acid	985	
	Ethyl palmitate	12366	
	9,12-Octadecadienoic acid (Z, Z)-	5280450	
	Octadec-9-enoic acid	965	
	Linoleic acid ethyl ester	5282184	
	Diisooctyl adipate	66932	
	Campesterol	173183	
	(23S)-ethylcholest-5-en-3.beta.-ol	91750025	
	Pentacosane	12406	
	Hexacosane	12407	

	Heptadecane	12398	
	Eicosane	8222	
	(E)-5,10-secocholest-1(10)-en-3,5-dione	15227029	
	(23S)-ethylcholest-5-en-3.beta.-ol	91750025	
	N-Methyl-L-prolinol	643492	
	4H-Pyran-4-one, 2,3-dihydro-3,5-dihydroxy-6-methyl	119838	[153]
	Hexadecanoic acid, ethyl ester	12366	
	2-Furancarboxaldehyde, 5-methyl-	12097	
	4H-Pyran-4-one, 2,3-dihydro-3,5-dihydroxy-6-methyl	119838	
	2-Furancarboxaldehyde, 5-methyl-	12097	
	Hexadecanoic acid, ethyl ester	12366	
70. Capsicum frutescens	fatty acid		[154]
	2-acetylfuran	14505	
	hexanal	6184	
	6-methyl-5-hepten-2-one a	9862	
	2-pentyl-furan	19602	
	3-carene b	26049	
	p-cymene b	7463	
	5-ethylfurfural	89989	

	phenylacetaldehyde	998	
	(E.E)-3.5-octadien-2-one	5352876	
	4-methyl-benzaldehyde	7725	
	(E.E)-3.5-nonadien-2-one	5368916	
	6-methyl-3.5-heptadien-2-one a	5370101	
	2.6-dimethyl-cyclohexanol	21428	
	3-hydroxy-2.3-dihydromaltol	119838	
	1-acetyl-pyrrolidine	77650	
	2.4-dimethyl-benzaldehyde	61814	
	safranal a	61041	
	m-cumenol	12059	
	b-cyclocitral a	9895	
	trans-9-decalol	137141	
	estragole	8815	
	2-(2-methylpropylidene)-cycloheptanone	5365596	
	a-copaene b	12303902	
	b-elemene	6918391	
	tetradecane	12389	
	a-ionone a	10846059	
	dihydro-b-ionone a	519382	

	dihydropseudoionone a	1549778	
	methyl dodecanoate	8139	
	dihydroactinidiolide a	6432173	
	heptadecane	12398	
	methyl tetradecanoate	31284	
	Alachlor	2078	[155]
	Ametoctradin	15604010	
	Benfuresate	3034378	
	Chlorantraniliprole	11271640	
	Chlorfluazuron	91708	
	Cyprodinil	86367	
	Dimethylvinphos	6433329	
	EPN	16421	
	Fenothiocarb	44178	
	Fenoxanil	11262655	
	Fenthion	3346	
	Flumioxazine	92425	
	Fluopyram	11158353	
	Imibenconazole	93483	

	Metolachlor	4169	
	Metribuzin	30479	
	Pendimethalin	38479	
	Penthiopyrad	11388558	
	Phorate	4790	
	Phosalone	4793	
	Picoxystrobin	11285653	
	Prochloraz	73665	
	Prometryn	4929	
	Propiconazole	43234	
	Silafluofen	92430	
	Simeconazole	10085783	
	Simetryn	13905	
	Terbutryn	13450	
	Tolclofos-methyl	91664	
	Alachlor	2078	
	Ametoctradin	15604010	
	Benfuresate	3034378	
	Chlorantraniliprole	11271640	
	Chlorfluazuron	91708	

	Cyprodinil	86367	
	Difenoconazole	86173	
	Dimethoate	3082	
	Dimethylvinphos	6433329	
	EPN	16421	
	Fenothiocarb	44178	
	Fenoxanil	11262655	
	Fenthion	3346	
	Flumioxazine	92425	
	Fluopyram	11158353	
	Imibenconazole	93483	
	Metolachlor	4169	
	Metribuzin	30479	
	Pendimethalin	38479	
	Phosalone	4793	
	Picoxystrobin	11285653	
	Prochloraz	73665	
	Prometryn	4929	
	Propiconazole	43234	
	Silafluofen	92430	

	Simeconazole	10085783	
	Simetryn	13905	
	Spiromesifen	9907412	
	Tebupirimfos	93516	
	Terbutryn	13450	
	Tolclofos-methyl	91664	
	Phenylalanine	6140	[156]
	3-phenyl Butane-2-ol	10642088	
	Butane, 2,3 diol	262	
	Lactic acid	612	
	ethyl malonate	7761	
	Acetamide	178	
	Pentanoic acid, 4-oxo-	11579	
	alpha.-D-Galactopyranose	439357	
	Butanedioic acid	1110	
	Benzoic acid	243	
	2-Hydroxy-4-methylpentanoic acid	92779	
	2,5-Furandicarboxylic acid	76720	
	Hexadecanoic acid, ethyl ester	12366	
	Pentanoic acid, 4-methyl-	12587	
	Benzeneacetic acid,	162368088	

	2,5-Furandicarboxylic acid	76720	
	Hexadecanoic acid, ethyl ester	12366	
	1H pyrazole, 4, 5 dihydro 1 phenyl		[157]
	Cyclopentadecanone, 2-hydroxy	543400	
	Z, E-2-methyl-3, 13-octadecadien-1-ol	5364521	
	4H-1-benzopyran-4-one, 5,7-dimethoxy-2-phenyl	88881	
	Cyclopropaneoctanoic acid 2-octyl-methyl ester,trans	91692412	
	1-Tetradecene, 2-decyl	544737	
	Methyl dihydrohydnocarpate	535041	
	13, 16-octadecadienoic acid methyl ester	5364511	
	16-Octadecenoic acid methyl ester	5364513	
	5-Methoxycarbonyltubercidin	272091	
	13-Docosenoic acid methyl ester	5363109	
	Cyclopentaneundecanoic acid methyl ester	535041	
	Ergosta-5, 22-dien-3-ol (Brassicasterol)	5281327	
	Piperidine, 4-(4-methylphenyl)-	544751	
	Phytol	5280435	
	Z, E-2-methyl-3, 13-octadecadien-1-ol	5364521	
	2,2,2-trifluoro-acetamide	67717	[158]

71.<br>Carica papaya			
	Methyl 9-methyltetradecanoate	554137	
	Hexadecanoic acid methyl ester	8181	
	Cis-10-nonadecenoic acid methyl ester	14178778	
	Trimethyl silanol	66110	
	Dodecamethyl cyclohexasiloxane	10911	
	Glycerin	753	
	Benzyl nitrile	8794	
	Tetradecanoic acid methyl ester	31284	
	Benzoic acid	243	
	Benzeneacetic acid	999	
	Isothiocyanatomethyl benzene		
	Benzeneacetamide	7680	
	Dodecanoic acid	3893	
	Tetradecanoic acid	11005	
	Hexadecanoic acid methyl ester	8181	
	Methyl stearate	8201	
	Cis-13-octadecenoic acid, methyl ester	12541027	
	9(Z)-Octadecenoic acid	445639	
	10-Octadecenoic acid methyl ester	25642	
	Cis-6-Octadecenoic acid	5281125	

	Cis-11-octadecenoic acid, methyl ester	5364505	
	1,1,3,3,5,5-hexamethyl trisiloxane	6327152	
	Benzeneacetamide	7680	
	Trimethylsilyl 2-(2-(2-butoxyethoxy)ethoxy)acetate	91696332	
	Squalene	638072	
	2,3-Dihydroxypropyl elaidate	5364833	
	Campesterol	173183	
	Stigmasterol	5280794	
	β-Sitosterol	222284	
	Stigmast-4-en-3-one	5484202	
	5α-Stigmastane-3,6-dione	13992092	
	4-hydroxybenzaldehyde	126	
	N,N’-Bis(trimethylsilyl)trifluoroacetamidine	91696742	
	Benzeneacetamide9	7680	
	Ethanamine	6341	
	Galactose methoxyamine	6421155	
	4-hydroxybenzenemethanol	125	
	Nonanoic acid	8158	
	Dodecanoic acid9	3893	
	Octanedioic acid	10457	

	Tetradecanoic acid9	11005	
	Hexadecanoic acid methyl ester	8181	
	Trans-9-octadecenoic acid methyl ester9	5280590	
	Pentadecanoic acid	13849	
	Cis-9-hexadecenoic acid	445638	
	Hexadecanoic acid9	985	
	Cis-9-octadecenoic acid	445639	
	Cis-6-octadecenoic acid9	5281125	
	Cis-9, cis-12-octadecadienoic acid methyl ester	5284421	
	Cis-13-octadecenoic acid, methyl ester	12541027	
	Cis-10-heptadecenoic acid	5312435	
	Heptadecanoic acid	10465	
	Trans-13-octadecenoic acid9	6161490	
	Linoleic acid	5280450	
	Trans-11-octadecenoic acid	5281127	
	Octadecanoic acid	5281	
	Cis-13-octadecenoic acid9	5312441	
	Cis-9-octadecenoic acid9	445639	
	Nonadecanoic acid	12591	
	11-eicosenoic acid	5282768	

	Eicosanoic acid	10467	
	Heneicosanoic acid	16898	
	Cis-vaccenic acid9	5282761	
	2-hydroxy-1-hexadecanoic acid ethyl ester9	18942997	
	Octadec-9-enoic acid	965	
	Oleic acid eicosyl ester	6436542	
	9-hexadecenoic acid	5282745	
	Cis-13-docosenoic acid	5281116	
	Docosanoic acid	8215	
	Octadecanoic acid 2,3-dihydroxypropyl ester	24699	
	Cis-15-tetracosenoic acid	5281120	
	Tetracosanoic acid	11197	
	Pentacosanoic acid	10468	
	Hexacosanoic acid	10469	
	Cis-13-octadecenal	5364497	
	Cis-9-octadecenal	5364492	
	Glycerol9	753	
	Propylene glycol	1030	
	Uracil	1174	
	Bromazepam	2441	

	2-ethylacridine	610161	
	Glycolic acid	757	
	Benzoic acid9	243	
	Phosphoric acid	1004	
	Nicotinic acid	938	
	Benzeneacetic acid9	999	
	Succinic acid	1110	
	Methylsuccinic acid	10349	
	Mandelic acid	1292	
	Pyroglutamic acid	7405	
	Pyrogallic acid	1057	
	3-hydroxybenzoic acid	7420	
	Phenyllactic acid	1303	
	1,4-benzenedicarboxylic acid	7489	
	Azelaic acid	2266	
	2-keto-l-gluconic acid	10198044	
	Phenol	996	
	2,4-bis(1,1-dimethylethyl)-phenol	7311	
	Octamethyltrisiloxane	24705	
	Sitostenone9	5484202	

	5α-stigmastane-3,6-dione	13992092	
	Campesterol9	173183	
	Stigmasterol	5280794	
	β-Sitosterol9	222284	
	Stigmastanol9	241572	
	Cycloartenol9	92110	
	β- d -(+)-xylopyranose	125409	
	4-hydroxybenzoic acid	135	
	Sucrose	5988	
	d -(+)-trehalose	7427	
	Dianhydromannitol	23619611	[159]
	Hexadecanoic acid, methyl ester	8181	
	Octadecanoic acid, methyl ester	8201	
	1,1,3,3,5,5,7,7,9,9,11,11-Dodecamethylhexasiloxane	6329090	
	Ergosta-5,22-dien-3-ol acetate (3β, 22E)	13889456	
	10-Octadecenoic acid, methyl ester	12830109	
	Saponins	6540709	
	n-Hexadecanoic acid	985	
	Phytol	5280435	

	Linolenic acid	5280934	
	Oleoyl chloride	5364783	
	Carpaine	442630	
	2,4 -Di-tert-butylphenol	7311	[160]
	Dihydroactinidiolide	27209	
	(-)-Loliolide	100332	
	Carpaine	442630	
	2(4H)-Benzofuranone, 5,6,7,7a-tetrahydro -6-hydroxy-4,4,7a-trimethyl-	14334	[161]
	4-Heptafluorobutyryloxyhexadecane	543578	
	Cyclotetradecane	67524	
	Neophytadiene	10446	
	2-Pentadecanone, 6,10,14-trimethyl	10408	
	cis-5-Decen-1-yl acetate	5363513	
	Trans-Farnesol	445070	
	Hexadecanoic acid, methyl ester	8181	
	n-Hexadecanoic acid	985	
	9,12-Octadecadienoic acid (Z,Z)-,methyl ester	5284421	
	Cardiac glycosides	439501	
	Anthraquinone	6780	

	Saponins	6540709	
	Cyclooctene, 3-ethenyl-	5365641	
	Phytol	5280435	
	Heptadecanoic acid, 16-methyl-, methyl ester	110444	
	9,12-Octadecadienoic acid (Z,Z)-	5280450	
	4,8,12,16-Tetramethylheptadecan-4- olide	567149	
	(2E,6E)-3,7,11-Trimethyldodeca-2,6 ,10-trienyl propionate	6431104	
	1-Chloroeicosane	39150	
	Mono(2-ethylhexyl) phthalate	20393	
	Ethanol, 2-(dodecyloxy)-	24750	
	Squalene	638072	
	Carbonic acid, decyl tetradecyl ester	91693142	
	Delta.-Tocopherol	92094	
	1-(1,4-cyclohexadienyl)-2-methylaminopropane	57487843	[162]
	Formamide, TMS derivative	13159475	
	p-Xylene	7809	
	Benzenepropanoic acid, .alpha.-(hydroxyimino)-	6416112	
	Melezitose	92817	
	4-Mercaptophenol	240147	
	Cyclohexanone, 2-(2-butynyl)-	584585	

	1-Tetradecyl acetate	12531	
	Melezitose	92817	
	2,4-Di-tert-butylphenol	7311	
	1-Dodecanol, 3,7,11-trimethyl-	138824	
	Tetradecanoic acid	11005	
	Acetic acid, 3,7,11,15-tetramethyl-hexadecyl	545558	
	Neophytadiene	10446	
	2-Hexadecene, 3,7,11,15-tetramethyl	5366161	
	Phytol, acetate	6428538	
	3,7,11,15-Tetramethyl-2-hexadecen-1-ol	5366244	
	n-Hexadecanoic acid	985	
	Hexadecanoic acid, ethyl ester	12366	
	Phytol	5280435	
	9,12,15-Octadecatrienoic acid,	860	
	Ethyl 9.cis.,11.trans.-octadecadienoate	9963693	
	3-trsns-(1,1-dimethylethyl)-4-trans-methoxycyclohexanol	545980	
	D-Limonene	440917	
	9,12-Octadecadienoic acid	3931	
	Butyl 9,12,15-octadecatrienoate	5743460	
	Squalene	638072	

	.gamma.-Tocopherol	92729	
	Octadecanoic acid, 4-hydroxy-, methyl	118556750	
	Campesterol	173183	
	Stigmasterol	5280794	
	.gamma.-Sitosterol	457801	
	9-Octadecenoic acid, (E)-	637517	
	17-Octadecynoic acid	1449	
	N-Ethyl-2-phenethylamine	547324	
	l-Alanine, N-methoxycarbonyl-, heptyl ester	6421859	
	p-Xylene	7809	
	1-(3,3,3-Trifluoro-2-hydroxypropyl)piperidine	566669	
	2-Azido-2,4,4,6,6-pentamethylheptane	546203	
	D-Limonene	440917	
	beta.-D-Glucopyranose, 1-thio-,1-[N-hydroxy-5-(methylthio)pentanimidate]	90658389	
	Melezitose	92817	
	4-Mercaptophenol	240147	
	Cyclohexanone, 2-(2-butynyl)-	584585	
	2,4-Difluorobenzene, 1-benzyloxy-	561269	
	2,4-Di-tert-butylphenol	7311	

	1-Dodecanol, 3,7,11-trimethyl-	11005	
	Tetradecanoic acid	11005	
	Neophytadiene	10446	
	Phytol, acetate	6428538	
	n-Hexadecanoic acid	985	
	Hexadecanoic acid, ethyl ester	12366	
	1-Heptatriacotanol	537071	
	Phytol	5280435	
	Oleic Acid	445639	
	1-Heptatriacotanol	537071	
	Hexadecanoic acid, 2-hydroxy-1-(hydroxymethyl)ethyl ester	123409	
	9,12-Octadecadienoic acid (Z,Z)-, 2-hydroxy-1-(hydroxymethyl)ethyl ester	5365676	
	Linolenic acid, 2-hydroxy-1-(hydroxymethyl)ethyl ester (Z,Z,Z)-	5367459	
	Squalene	638072	
	dl-.alpha.-Tocopherol	2116	
	Stigmasterol	5280794	
	.gamma.-Sitosterol	457801	
	Cholest-5-en-3-ol, 24-propylidene-, (3.beta.)-	6443745	
	13,27-Cycloursan-3-one	634605	

	9,19-Cyclolanost-24-en-3-ol, acetate, (3.beta.)-	518616	
	Campesterol	173183	
	Dasycarpidan-1-methanol, acetate (ester)	550072	
	9-Octadecenoic acid, 1,2,3-propanetriyl ester, (E,E,E)-	5364673	
72. Cassia alata	Neophytadiene	10446	[163]
	6,10,14-trimethyl-2-pentadecanone	10408	
	Methyl palmitate	8181	
	Palmitic acid	985	
	Ethyl hexadecanoate	12366	
	Methyl linoleate	5284421	
	Methyl 11-octadecenoate	5364432	
	Methyl stearate	8201	
	Linoleic acid	5280450	
	(E)-9-Octadecenoic acid	637517	
	Stearic acid 4,8,12,16-	101273371	
	azabicyclo[4.3.1] deca-3,7-diene-10-carboxylate	73050129	
	Erucylamide	5365371	
	n-Eicosane	8222	
	Hexadecyleneoxide	14985	
	Vitamin E	519006	

	Stigmasterol	5280794	
	Methylpentadecanoate	13072095	[164]
	n-Hexadecanoic acid	985	
	Methyloctadec-9-enoate	5280590	
	6-Octadecenoic acid	5282754	
	Octadecanoic acid	5281	
	Glycerol-1,3-dipalmitate	102142839	
	9-Octadecenoyl chloride	66987	
	9-Octadecenal	5283381	
	4-Dimethylsilyloxypentadecane	6328913	
	2,3-Dihydroxypropyl-9-octadecenoate	33022	
	8,11,14 Eicosatrienoic acid,	5280581	
	Phytol	5280435	
	Oleic acid	445639	
	trans -Farnesol	445070	
	Behenic alcohol	12620	
	1-Heptatriacotanol	537071	[165]
	Citronellic acid	10402	

	Nerolidol	5284507	
	Stigmasterol	5280794	
	1-Heptatriacotanol	537071	
	Palmitoleic acid	445638	
	Limonene<br>6-Dodecanone	22311<br>80165	
	2-Butylhydrazone	39671	
	β-D-Glucopyranoside	440936	
	Glycolaldehyde dimer	186078	
	α-d-Mannofuranoside	57369919	
	Decanoic acid	2969	[166]
	n-Hexadecanoic acid	985	
	11,14,17-Eicosatrienoic acid	5282827	
	Oleic acid	445639	
	3-Decen-1-ol	5352493	
	Propanoic acid, 2-oxo-,methyl ester	11748	
	Furfural	7362	
	2-Furanmethanol	7361	
	2-Cyclopentene-1,4-dione	70258	
	Butanoic acid, 4-hydroxy	10413	

	1,2-Cyclopentanedione	566657	
	2- Furancarboxaldehyde, 5-methyl-	12009549	
	2-Methoxy-4-vinylphenol	332	[167]
	Phenol,2,6-dimethoxy-	5352905	
	D-Allose	439507	
	3-O-Methyl-d-glucose	8973	
	n-Hexadecanoic acid	985	
	6-Octadecenoic acid,(Z)	21776357	
	α-D-Glucopyranoside, α-D-glucopyranosyl	131864692	
	Phenyl carbamate	69322	
	Xylitol	6912	
	Succinamic acid	12522	
	Cyclohexene-3,5-diol	565903	
	2-methoxy-4-vinyl phenol	332	
	2,3-bis-(4-methoxy phenyl)-1,4,5-trimethyl	590632	
	piperizine	4837	
	2,6,6-trimethyl-1-cyclohexene-1-carboxaldehyde	9895	
	1-chloro-octadecane	18815	
	Methyl-4-(3-oxocyclohexyl)-butanoate	566170	

	Trispiro[4,2,4,2,4,2] heneicosane	566316	
	n-butyl benzylsulfonamide	5224617	
	6,10-dimethyl-2-undecanone	95495	[168]
	4-dodecene-1-ol	5362820	
	3,7,11,15-tetramethyl-	5281365	
	hexadecene-1-ol	40467768	
	Methyl cis-9-octadecenoate	5364509	
	Methyl palmitoleate	643801	
	n-hexadecanoic acid methyl ester	8181	
	Palmitic acid	985	
	Methyl cis,cis-9,12-octadecadienoate	5284421	
	Methyl cis-9-octadecenoate	5364509	
	Trans -1,3-trans-1,4-menthol	16666	
	Methyl stearate<br>Decylmercaptan	8201<br>8917	
	Methyl linolelaidate	5362793	
	Eicosanoic acid methyl ester	14259	
	4,8,12,16-tetramethylheptadecan-4-olide	567149	
	Gamma tocopherol	92729	
	Alpha tocopherol	14985	

	Tetracosanoic acid methyl ester	75546	
	Campesterol	173183	
	Stigmasterol	5280794	
	2-Methyl-1-octanol	102495	
	Pentanoic acid	7991	
	2-Ethyl-1-decanol	545566	
	Octadecanoic acid	5281	
73. Cassia auriculata	Dodecanoic acid	3893	[169]
	Hexadecanoic acid, ethyl ester	12366	
	8-Octadecanoic acid, methyl ester	8201	
	3-O-Methyl-d-glucose	8973	
	Desulphosinigrin	9601716	
	Neophytadiene	10446	
	Dotriacontane	11008	
	Phytol	5280435	
	Eicosyl isopropyl ether	91691499	
	dl-.alpha.-Tocopherol	2116	
	alpha.-Tocopherol-.beta.-D-mannoside	597057	

	Hexatriacontane	12412	
	Tetracosane	12592	
	Squalene	638072	
	Ergost-5-en-3-ol, (3.beta.)-	5283637	
	Cholest-23-ene, (5.beta.)-	21159906	
	4-Hydroxybenzoic acid, 2TMS derivative	519811	
	Cyclodecasiloxane, eicosamethyl	519601	
	Glycine, N-allyloxycarbonyl-, pent-10-enyl ester	91691244	
	5-Oxo-7-[4-(trifluoromethyl)phenyl]-4H,6H,7H-[1,2]thiazolo[4,5-b]pyridine	51138558	
	Stigmasterin	5280794	
	Stigmasterin	5280794	
	Octadecane, 3-ethyl-5-(2-ethylbutyl)-	292285	
	Hexadecane	11006	
	1-Octadecanol	8221	
	Heptadecane	12398	
	Pentadecane,2,6,10,14-tetramethyl-	15979	
	Octadecane	11635	
	Nonadecane	12401	
	Eicosane	8222	

	1-Eicosene	18936	
	Heneicosane	12403	
	Docosane	12405	
	1-Docosene	74138	
	Phenol,2,4-bis(1,1-dimethylethyl)-	93344	
	Tricosane	12534	
	Hentriacontane	12410	
	Hexadecanoic acid,butyl ester	86004092	
	n-Tetracosanol-1	10472	
	Pentacosane	12406	
	Phthalic acid, hept-4-yl isobutyl ester	91720280	
	Phthalic acid, butyl-2-pentyl ester	249800	
	Heptacosane	11636	
	Dibutyl phthalate	3026	
	1-Hexacosene	29303	
	12-Oxotricyclo [5.3.1.1(2,6)]dodeca-3,8-diene,11-acetoxy-4,5,9-trichloro	540683	
	Nonacosane	12409	
	17-Pentatriacontene	5365022	
	l-(+)-Ascorbic acid 2,6-dihexadecanoate	54722209	[170]
	i-Propyl 11,12-methylene-octadecanoate	91692516	

	Hexadecanoic acid	985	
	Bis(2-ethylhexyl)phthalate	8343	
	Octadecane,-3-ethyl-5-(2-ethylbutyl)-	292285	
	2-Oxiranemethanol, α-(1-methylethyl)-3-[1-(trimethylsilyloxy)pentyl]-	554431	
	D-Pinitol, pentakis(trimethylsilyl) ether	14534973	
	1,5-Anhydro-D-sorbitol, tetrakis(trimethylsilyl) ether	529471	
	Lactulose, octakis(trimethylsilyl) ether (isomer 1)	525319	
	Myo-Inositol, 1,2,3,4,5,6-hexakis-O-(trimethylsilyl)-	520232	
	D-(-)- Erythrofuranose, tris(trimethylsilyl) ether (isomer 2)	553108	
	D-(+)-Glucuronic acid γ-lactone, tris(trimethylsilyl) ether, methyloxime	91696698	
	Sedoheptulose, o-methyloxime, hexakis-O-(trimethylsilyl)-	9604182	
	Inositol, 1,2,3,4,5,6-hexakis-O-(trimethylsilyl)-, scyllo-	520232	
	D-(-)-Tagatose, pentakis(trimethylsilyl) ether	519639	
	D-(+)-Turanose, octakis(trimethylsilyl)ether	527544	
	9,10-Anthracenedione, 2-methyl-1,6-bis[(trimethylsilyl)oxy]-	633412	
	D-(-)-Ribofuranose, tetrakis(trimethylsilyl)ether(isomer 1)	523970	
	D-(-)-Ribofuranose, tetrakis(trimethylsilyl) ether (isomer 2)	523970	
	bis[2-Trimethylsiloxy]ethyl sulfone	553715	

	Resorcinol	5054	
	Glycerin	753	
	Thymine	1135	
	4H-Pyran-4-one,2,3-dihydro-3,5-dihydroxy-6- methyl	119838	
	Benzaldehyde,4 methyl-	15294	
	2-Propenoic acid, 4-methylpentyl ester	78823	
	Resorcinol	5054	
	Sucrose	5988	
	β-D-Glucopyranoside, methyl	445238	
	3-O-Methyl-d-glucose	8973	
	1,2-Benzenedicarboxylic acid,bis (2-methylpropyl)ester	6782	
	Benzenamine,2,3,4,5,6-pentamethyl	596025	
	n-Hexadecanoic acid	985	[171]
	Hexadecanoic acid, ethyl ester	12366	
	1-Tridecyne	117754	
	13-Oxabicyclo[10.1.0] tridecane	1549216	
	Phytol	5280435	
	1-E,11,Z-13-Octadecatriene	5365585	
	13-Octadecenal,(Z)-	5364497	
	1 Octadecanoic acid	5281	

	1,2,3,4-Tetrahydroisoquinolin-6-ol-1-carboxylic acid	590430	
	α- Tocopherol	14985	
74. Cassia fistula	1H-Indene, octahydro-, cis-	643587	[172]
	9-Heptadecanol	136435	
	Behenic alcohol	8215	
	Cyclohexane, butyl-	15506	
	Cyclohexene, 1-butyl-	137878	
	Decane, 3-methyl-	92239	
	Cyclohexane, 1-methyl-3-(1-methylethenyl)-,cis-	90470519	
	3-Hexanol, 5-methyl-	12186	
	Acetaldehyde isopentyl propyl acetal	53428151	
	Undecane	14257	
	1,3-Dioxane, 2-ethyl-5-methyl-	141976	
	Acetaldehyde butyl pentyl acetal	101588415	
	Acetaldehyde dipentyl acetal	83035	
	Cycloheptasiloxane, tetradecamethyl-	7874	
	Cyclooctasiloxane, hexadecamethyl-	11170	
	Cyclononasiloxane, octadecamethyl-	11172	
	Hexadecanoic acid	985	

	Tetracosamethyl-cyclododecasiloxane	167767	
	Oxacyclododecan-2-one	74409	
	Imidazole ,2-amino-5-[(2-carboxy)vinyl]-	5364104	
	D-Glucose , 6-O-±-D-galactopyranosyl-	71771812	
	2-Nonanone	13187	
	Eicosanoic acid ,phenylmethyl ester	562252	
	Phenol , 4-(2-propenyl)-	159137	
	Eugenol	3314	
	Caryophyllene	5281515	
	ß-copaene	21722369	
	±-acorenol	11972555	
	Spiro[5.5]undec-8-en-1-one	596233	
	Isoaromadendrene epoxide	534398	
	Tetraacetyl-d-xylonic nitrile	541568	[173]
	Benzyl Benzoate	2345	
	Phenethylamine ,3-benzyloxy-2-fluoroß-	560988	
	4a-Hydroxy-4-nitroperhydronaphthalen -1-one	580573	
	Dasycarpidan -1-methanol, acetate (ester)	101287780	
	Propanoic acid , 2-(3-acetoxy-4,4,14-trimethylandrost-8-en-17-yl)-	631957	

	Cis-13-Eicosenoic acid	5312518	
	Vitamin E	14985	
	2-Hydroxyethylhydrazine	8017	
	N-Hexadecanoic acid	985	
	Cyclotrisiloxane,hexamethyl	10914	
	Phytol	5280435	[174]
	Oleic acid	445639	
	Heptadecane, 2,6,10,14-tetramethyl-	29036	
	Octane, 2,6-dimethyl-	16319	
	Cyclohexanone, 2,3-dimethyl-	114508	
	Decane, 2,5,6-trimethyl-	112466	
	Nonane, 5-methyl-	27518	
	Nonane, 4-methyl-	28455	
	Nonane, 2-methyl-	13379	
	Benzene, 1-ethyl-3-methyl-	12100	
	Nonane, 3-methyl-	22202	
	Mesitylene	7947	
	Benzene, 1-ethyl-2-methyl-	11903	

	Decane<br>Benzene, 1,2,3-trimethyl-	15600<br>10686	
	Benzyl alcohol	244	
	Benzene, 1-methyl-3-propyl-	14092	
	Benzene, 1-methyl-4-propyl-	14095	
	Benzene, nitro-	7416	
	Tetradecane	12389	
	Naphthalene	931	
	Benzofuran, 2,3-dihydro-	10329	
	Resorcinol	5054	
	Hexadecane	11006	
	Phenol, 4-propyl-	12580	
	Cetene	12395	
	Pentadecane	12391	
	Isopropyl 2-ethylhexanoate	106701	
	5-Octadecene, (E)-	5364598	
	Nonadecane	12401	
	Hexacosane	12407	
	Trifluoroacetoxy hexadecane	522035	
	Heptadecane	12398	[175]
	Tetratetracontane	23494	

	17-Pentatriacontene	5365022	
	Sulfurous acid, butyl heptadecyl ester	6420807	
	Oxalic acid, allyl hexadecyl ester	6420236	
	2-Butenal, 2-ethyl-	5362897	
	1-Decanol, 2-hexyl-	95337	
	1-Heptanol, 2,4-diethyl-	549992	
	Octane, 2,5-dimethyl-	139988	
	Methyl-9,12,15-octadecatrienoate	5367462	
	Methyl octadecanoate	8201	
	Vitamin E (α-tocopherol)	86472	
	(Z)-Octadec-10-enyl acetate	5363223	[176]
	Hexamethylcyclotrisiloxane	10914	
	N-ethyl-1,3-dithioisoindoline	87520016	
	4-Nonylphenol	1752	
	Methyl hexadecanoate	8181	
	Phytol	5280435	
	n-Hexadecanoic acid (Palmitic acid)	985	
	2,6-Di-O-palmitoyl-L-ascorbic Acid	54722209	
	Heptadecanoic acid	10465	

	Hexadecanoic acid, ethyl ester (Ethyl palmitate)	12366	[177]
	Tetradecanoic acid, ethyl ester (Myristic acid)	31283	
	9,12,15-Octadecatrienoic acid, methyl ester (Linolenic acid, methyl ester)	9316	
	9,12-Octadecadienoic acid (cis-Linoleic acid)	5282798	
	9,12-Octadecadienoic acid (Linoleic acid methyl ester)	6439705	
	Oxacycloheptadec-8-en-2-one	5365703	
	9,12,15-Octadecatrienoic acid (alpha.-Linolenic acid)	86715130	
	9,12,15-Octadecatrienoic acid, ethyl ester (Meed acid)	8122	
	Vitamin E (alpha.-Tocopherol)	2734086	
	Beta.-Tocopherol	6857447	
	Gamma.-Tocopherol	92729	
	5-(Hydroxymethyl)-2-(dimethoxymethyl)furan	588127	
	Tetradecane	12389	
	3-Aminodibenzofuran	20061	
	2,4-Di-tert-butylphenol	7311	
	1-Octadecene	8217	
	1-Eicosene	18936	
	Methyl palmitate	8181	
	Palmitic acid	985	[178]

	Methyl linoleate	5284421	
	Methyl oleate	5364509	
	Methyl stearate	8201	
	Methyl linoleate	5284421	
	9-Octadecenamide/oleamide E	6197971	
	1-Nonanol	8914	
	methyl-14-methylpentadecanoate	21205	
	1,E-11,Z-13-octadecatriene	5365585	
	palmitic acid, methyl ester	8181	
	palmitic acid	985	
	9-octadecenal	5283381	
	methyl-11-octadecenoate	5364432	
	Methyllinolelaidate		[179]
	stearic acid	5281	
	oleic acid	445639	
	arachidic acid	10467	
	α-monopalmitin	14900	
	brassidic acid	5282772	
	capric acid methyl ester	8050	

	undecylenic acid	5634	
	hendecanoic acid	8180	
	lauric acid	3893	
	n-tridecanoic acid methyl ester	15608	
75. Catharanthus roseus	Hexadecanoic acid, methyl ester	8181	[180]
	n-Hexadecanoic acid	985	
	Hexadecanoic acid, ethyl ester	12366	
	9-Octadecenoic acid (Z)-methyl ester	5364509	
	1,2-Benzenedicarboxylic acid, diisooctyl ester	33934	
	Dodecanoic acid, methyl ester	8139	
	Methyl tetradecanoate	31284	
	3,7,11,15-Tetramethyl-2-hexadecen-1-ol	5366244	
	Phytol	5280435	
	4H-Pyran-4-one	7968	[181]
	n-Hexadecanoic acid	985	
	Phytol	5280435	
	4H-Pyran-4-one	7968	[182]
	Catechol/Resorcinol	19354071	
	1,2,3-Propanetriol, 1-acetate/acetin	33510	

	L-Glucose	10954115	
	Ascaridole epoxide	10545	
	Deoxyspergualin	91272	
	Sucrose	5988	
	D-fructose	2723872	
	D-allose	439507	
	Desulphosinigrin	9601716	
	1,2,3,5-Cyclohexanetetrol+	548226	
	Muco-Inositol	892	
	Hexadecanoic acid, methyl ester	8181	
	Pentadecanoic acid	13849	
	Phytol	5280435	
	9,12,15-Octadecatrienoic acid,methyl ester	9316	
	2,20-Cycloaspidospermidine-3-carboxylic acid, Vindolinine	78358491	
	9,12,15-Octadecatrienoic acid,	860	
	Octadecane,3-ethyl-5-(2-ethylbutyl)-	292285	
	Condyfolan, 14,19-didehydro-12-methoxy-, (14E)-	5372292	
	Phthalic acid, di(oct-3-yl) ester	15764573	
	4H-Pyran-4-one	7968	[183]

	Catechol/Resorcinol	19354071	
	1,2,3-Propanetriol, 1-acetate/acetin	33510	
	Chlorozotocin	451706	
	l-Gala-l-ido-octonic lactone	219893	
	Limonen-6-ol, pivalate	545235	
	Sucrose	5988	
	Desulfosinigrin	134160268	
	2-Pyrrolidinone, 1-butyl-	18984	
	3’,5’-Dimethoxyacetophenone	95997	
	Ethyl N-(o-anisyl) formimidate	601627	
	1,2,3,5-Cyclohexanetetrol	548226	
	Ethyl N-(o-anisyl) formimidate	601627	
	1,2,3,5-Cyclohexanetetrol	548226	
	Tetraacetyl-d-xylonic nitrile	541568	
	Myo-Inositol, 4-C-methyl-	244581	
	2-Pentyne-1,4-diol, 4- methyl-1-(2-thienyl)-	5151212	
	Psicofuranine	65086	
	Cis-Inositol	892	
	Muco-Inositol	892	
	Hexadecanoic acid, methyl ester	8181	

	Pentadecanoic acid	13849	
	3-Phenylbicyclo (3.2.2) nona-3,6-dien-2-one	610888	
	Dasycarpidan-1-methanol, acetate (ester	550072	
	9,12-Octadecadienoic acid (Z, Z)-, methyl ester	5284421	
	9-Octadecynoic acid	68167	
	Methyl 8,11,14-heptadecatrienoate	85978449	
	1,1’-Bicyclopropyl]-2-octanoic acid,2’-hexyl-, methyl ester	552098	
	9,12,15-Octadecatrienoic acid,	860	
	N-desmethyltramadol	198555	
	Prednisolone hemisuccinate	656804	
	Benzaldehyde	240	[184]
	Heptanal	8130	
	Linalool	6549	
	3-octen-2-one	5363229	
	(.+ -.)-Menthol	1254	
	Geraniol	637566	
	Decanoic acid	2969	
	Geranyl Acetone	1549778	
	γ-Eudesmol	6432005	
	Tetradecane	12389	

	α-Eudesmol	92762	
	Tetradecanoic acid	11005	
	Nonadecane	12401	
	Heneicosane	12403	
	Tricosane	12534	
	Octadecanoic acid	5281	
	Tetracosane	12592	
	Phytol	5280435	
	Tetradecanoic acid	11005	
	Palmitic acid	985	
	Ethyl ester of Hexadecanoic acid	12366	
	Methyl ester of 9,12- octadecadienoic acid Z,Z)-	5362793	
	Stearic acid	5281	
	Ethyl Oleate	5363269	
	Ethyl ester of Octadecanoic acid	8122	
	Methyl ester of Eicosanoic acid	14259	
	Neophytadiene	10446	
	9,12,15-Octadecatrienoic acid, methyl ester (CAS)	5319706	[185]
	Beta Ionol	5373729	

	2-Hexadecen-1-ol, 3,7,11,15-tetramethyl	5366244	
	Neophytadiene	10446	
	Patchouli alcohol	10955174	
	Lycopene 7	446925	
	Coronaridine	6426909	
	Hexadecanoic acid, methyl ester (CAS)	8181	
	9,12,15-Octadecatrienoic acid, methyl ester (CAS)	5319706	
	Piperidine, 3-phenyl-	107207	
	Vitamin E acetate	86472	
	Hexadecanoic acid, methyl ester (CAS)	8181	
	Ethyl linoleate	5282184	
	Neophytadiene	10446	
	Bicyclo[3.3.1]non-6-ene-3,9-dione	556259	
	Ar turmerone	160512	
	(Z)6-Pentadecen-1-ol	5365626	
	Vitamin E acetate	86472	
	Ar turmerone	160512	
	beta.-Ionol	5373729	
	Neophytadiene	10446	
	9,12,15-Octadecatrienoic acid, methyl ester, (Z,Z,Z)- (CAS)	5319706	

	Neophytadiene	10446	
	Farnesol	445070	
	Ar-Tumerone	558221	
	Alpha.-turmerone	14632996	
	Ethyl linoleate	5282184	
	Dodecanoic acid, 10-undecen-1-yl ester	543988	
	9,12,15-Octadecatrienoic acid, methyl ester, (Z,Z,Z)- (CAS)	5319706	
	Camphor (CAS)	2537	
	9,12,15-Octadecatrienoic acid, methyl ester, (Z,Z,Z)- (CAS)	5319706	
	4-Pyridinecarboxamide (CAS)	15074	
	Coronaridine	6426909	
	Citronellyl Acetate	9017	
	Neophytadiene	10446	
	Ar-Tumerone	558221	
	Alpha.-turmerone	14632996	
	Hexadecanoic acid, methyl ester (CAS)	8181	
	Citronellyl Valerate	61416	
	Neophytadiene	10446	
	Vitamin E acetate	86472	
	Ar-Tumerone	558221	

	Alpha.-turmerone	14632996	
	Hexadecanoic acid, methyl ester	8181	[186]
	n-Hexadecanoic acid	985	
	Octadecanoic acid, methyl ester	8201	
	9, 12, 15-Octadecatrienoic acid, methyl ester,	9316	
	1, 2-Benzenedicarboxylic acid, mono(2-ethylhexyl) ester	20393	
	Myristic acid	11005	[187]
	Palmitic acid	985	
	Adipic acid, bis(2-ethylhexyl) ester	7641	
	Stearic acid	5281	
	Tridecanoic acid, methyl ester	15608	
	Pentadecanoic acid	13849	
	Octadecanoic acid	5281	
	Heptadecanoic acid	10465	
	Eicosanoic acid	10467	
	Isopropyl palmitate	8907	
	6,9—Octadecadienoic acid, methyl ester	5365662	

	Oleic acid, Trimethylsilyl ester	5366433	
	Methyltetradecanoate	31284	[188]
	Vincoline	3032566	
	Pericyclivine	44224213	
	Vindoline	260535	
	B-Sitosterol	222284	
	Hexadecanoic acid	985	
	Oleic acid	445639	
	Tetracosane	12592	
	10-Heneicosene	5364553	[189]
	Camphene	6616	[190]
	Methyleugenol	7127	
	2-heptanol, 5-ethyl	29775	
	9, 12-octadecadienic acid	3931	
	n-Hexadecanoic acid (palmitic acid)	985	[191]
	Phytol	5280435	
	9,12,15-Octadecatrien-1-ol	5367327	
	2-Methylpentane	7892	[192]

76. Centaurium erythraea			
	3-Methylpentane *	7282	
	Hexane	8058	
	Ethyl acetate	8857	
	Methylcyclopentane	7296	
	Cyclohexane	8078	
	Pentanal	8063	
	Toluene	1140	
	Hexanal	6184	
	1,4-Dimethylbenzene *	70755	
	Heptanal	8130	
	α-Thujene	12444324	
	α-Pinene	6654	
	Propylbenzene (Isocumene)	7668	
	m-Ethyltoluene *	12100	
	1,2,4-Trimethylbenzene (Pseudocumene)	7247	
	Sabinene	18818	
	β-Pinene	14896	
	o-Ethyltoluene *	11903	
	2-Pentylfuran	19602	
	1,2,3-trimethylbenzene (Hemimellitene)	10686	

	p-Cymene	7463	
	Limonene	22311	
	γ-Terpinene	7461	
	α-Terpinolene	11463	
	Undecane	14257	
	Linalool	6549	
	Nonanal	31289	
	β-Thujone	261491	
	Camphor	2537	
	Menthone	26447	
	Isomenthone	6432469	
	Menthol	1254	
	Terpinen-4-ol	11230	
	Naphthalene	931	
	α-Terpineol	17100	
	Decanal	8175	
	Verbenone	29025	
	Bornyl acetate	93009	
	Safrole	5144	
	Menthyl acetate	27867	

	Tridecane	12388	
	α-Copaene	481539140	
	(E)-β-Damascenone	12389	
	Tetradecane<br>Longifolene<br>289151	12389	
	β-Caryophyllene	5281515	
	trans-β-Farnesene	5281517	
	Hexadecane	11006	
	Heptadecane	12398	
	Nonadecane	12401	
77. Chenopodium ambrosioides	α-phellandrene	198941074	[193]
	α-pinene	258567152	
	β-myrcene	348293176	
	gamma-terpinen	7461	
	D-limonene	440917	
	4-carene	530422	
	o-cymene	10703	
	p-cymen-8-ol	14529	
	4-carene	530422	
	α-terpinene	198978036	[194]

	p-cymene	7463	
	trans- β-ocimene	249959748	
	γ-terpinene	348276756	
	terpinolene	11463	
	ascaridol	10545	
	neral	643779	
	geraniol	637566	
	carvacrol	10364	
	isoascaridol	351031	
	myrcene	31253	[195]
	Alpha-terpinene	7462	
	p-cymene	7463	
	limonene	22311	
	terpinolene	11463	
	p-cymenene	62385	
	terpinen-4-ol	11230	
	p-cymen-8-ol	14529	
	Alpha-terpineol	17100	
	ascaridole	10545	
	thymol	6989	

	carvacrol	10364	
	isoascaridole	12314661	
	périllaldehyde	1548901	
	b-caryophyllene	5281515	
	α-Pinene	258567152	[196]
	β-Pinene	198976008	
	δ-4-Carene		
	α-Terpinene	198978036	
	ρ-Cymene	463932906	
	2-Ethylcyclohexanone	20474	
	γ-Terpinene	348276756	
	α,α-4-Trimethylbenzyl alcohol	319072553	
	p-Cymen-8-ol	14529	
	α-Terpineol<br>cis-Piperitol	175271399<br>85567	
	(Z)-Ascaridole	12308625	
	Piperitone	6987	
	Piperitone oxide	92998	
	Thymol	6989	
	Isoascaridole	12314661	
	Carvacrol	10364	

	Precocene II	12565	
	Elemicin	10248	
	Caryophyllene oxide	1742210	
	Allyltetramethoxybenzene	617233	
	Asarone	636822	
	Geranyl tiglate	5367785	
	Phytol	5280435	
	Alpha-Pinene	258567152	[197]
	Alpha-Terpinene	198978036	
	p-Cymene	463932906	
	Limonene	22311	
	-Phellandrene	7460	
	Alpha-Terpinene	198978036	
	Dehydro-p-cymene	62385	
	p-Mentha-1,8-diene	22311	
	Ascaridole	10545	
	Thymol	6989	
	Carvacrol	10364	
	Isoascaridole	12314661	
	p-Mentha-1,3,8-triene	176983	[198]

	α-Terpineol	175271399	
	(Z)-Ascaridole	12308625	
	Piperitone	6987	
	Carvacrol	10364	
	(Z)-Carvyl acetate	102024	
	α-Terpinene	198978036	
	p-Cymene	463932906	
	Benzyl alcohol	244	
	p-Cresol	2879	
	alpha-Terpinene	198978036	[199]
	para-Cymene	463932906	
	Pinocarvone	121719	
	Ascaridol<br>Dihydrocarvyl acetate	10545<br>30248	
	Piperitone oxide	92998	
	y -Terpinene	7461	
	Aritasone	50757403	
	Benzaldehyde	240	
	Limonene oxide	91496	
	Terpinolene	11463	

	3-Methoxysalicylic acid	70140	
	5-Isopropenyl-2-methyl-7-oxabicyclo[4.1.0]heptan-2-ol	565280	
	Cyclohexanol, 2-methyl-5-(1-methylethenyl)-<br>3,7,11,15-Tetramethyl-2-hexadecen-1-ol	12072	
	E-7-Tetradecenol	5362726	
	Tetradecanoic acid	11005	
	1-Hexadecanol	2682	
	Phytol	5280435	
	9,12-Octadecadienoic acid (Z,Z)-	5280450	
	Octadecanoic acid, ethyl ester	8122	
	Didodecyl phthalate	17082	
	Squalene	638072	
78. Cinnamomum zeylanicum	Epigallocatechin	72277	[200]
	Ascorbic acid	54670067	
	Chlorogenic acid	1794427	
	Fumaric acid	444972	
	Verbascoside	5281800	
	Orientin	5281675	
	Caffeic acid	689043	

	Luteolin-7-rutinoside	10461109	
	Rutin	5280805	
	Rosmarinic acid	5281792	
	Hyperoside	5281643	
	Ellagic acid	5281855	
	Quercitrin	5280459	
	Quercetin	5280343	
	Herniarin	10748	
	Salicylic acid	338	
	Naringenin	439246	
	Luteolin	5280445	
	Apigenin	5280443	
	Hispidulin	5281628	
	Isosakuranetin<br>Penduletin	160481<br>5320462	
	CAPE	5281787	
	Chrysin	5281607	
	Quillaic acid	101810	
	Caryophyllene oxide	1742210	
	P-cymene	7463	[201]

	Heptanoic acid	8094	
	Benzenepropanal	7707	
	Linalool	6549	
	Linalyl propionate	61098	
	Borneol	64685	
	Cis-cinnamaldehyde	6428995	
	Trans-cinnamaldehyde	637511	
	Eugenol	3314	
	Copaene	12303902	
	Cinnamyl acetate	5282110	
	Trans-caryophyllene	5281515	
	Cinnamic acid	444539	
	α-Humulene	5281520	
	o-Methoxycinnamaldehyde	641298	
	Ledene	10910653	
	Gurjunene	15560275	
	α-Cadinene	12306048	
	Spathulenol	92231	
	Clovene	10102	
	Humuladienone	101297706	

	Bisabolol	1549992	
	Benzyl benzoate	2345	
	Phenethyl benzoate	7194	
	Isocalamendiol	12302240	
	Butyl phthalate	3026	
	Hexadecanoic acid	985	
	Ferruginol	442027	
	Phytol	5280435	
	α-pinene	6654	[202]
	terpin-4-ol	94116	
	linalool acetate	8294	
	geraniol	637566	
	cinnamaldehyde(trans)	637511	
	eugenol	3314	
	β-elemene	6918391	
	methyl eugenol	7127	
	ethyl cinnamate<br>coumarin(3-methyl-)	637758<br>17130	
	γ-cadinene	6432404	
	δ-cadinene	10657	
	humulene epoxide	5352470	

	turmerone	14367555	
	α-Pinene	6654	[203]
	Camphene	6616	
	Benzaldehyde	240	
	α-Phellandrene	7460	
	p-Cymene	7463	
	β-Phellandrene	11142	
	Eucalyptol	2758	
	Linalool	6549	
	Benzenepropanal	7707	
	Cis-cinnamaldehyde	6428995	
	Saffrole	717338	
	Trans-cinnamaldehyde	637511	
	Eugenol	3314	
	α-Cubebene	442359	
	Caryophyllene	5281515	
	Coumarin (2H-1-Benzopyran-2-one)	323	
	Cinnamyl acetate (E)	5282110	
	α-Muurolene		

	trans-cadina-1(6),4-diene	10798255	
	Eugenyl acetate	7136	
	Benzyl benzoate	2345	
	n-Hexan-2-ol	12297	[204]
	Heptan-2-one	8051	
	Alpha-Thujene	17868	
	Alpha-Pinene	6654	
	Camphene	6616	
	Sabinene	18818	
	Beta-Pinene	14896	
	Myrcene	31253	
	Alpha-Phellandrene	7460	
	3-Carene	442461	
	Alpha-Terpinene	7462	
	p-Cymene	7463	
	(E)-ˇ-Ocimene	6429141	
	gamma-Terpinene	7461	
	cis-Linalool oxide (furanoid)	22310	
	Terpinolene	11463	
	trans-Linalool oxide (furanoid)	6432254	

	Linalool	6549	
	2-Phenyl ethyl alcohol	95542	
	Camphor	2537	
	Citronellal	7794	
	Borneol	64685	
	Terpinen-4-ol	11230	
	(Z)-Methylcinnamate	6383065	
	Methylchavicol	8815	
	(Z)-Cinnamaldehyde	6428995	
	Nerol	643820	
	Piperitone	6987	
	Safrole	5144	
	Eugenol	3314	
	(Z)-Cinnamyl acetate	5315912	
	Beta-Caryophyllene	5281515	
	(E)-ˇ-Farnesene	5281516	
	Eugenyl acetate	7136	
	Alpha -Selinene	10856614	
	Delta-Cadinene	441005	
	(E)-Nerolidol	5284507	

	Spathulenol	92231	
	Beta-Caryophyllene oxide<br>Humulene epoxide<br>5352470	1742210	
	T-Cadinol	160799	
	Alpha-Cadinol	10398656	
	Alpha-n-Hexyl cinnamaldehyde	7585	
	Geranyl benzoate	5353011	
	Phenylethyl-n-decanoate	112733	
79. Cistus ladanifer	3-Carene	26049	[205]
	a-Phellandrene	7460	
	2,2,6-Trimethylcyclohexanone	17000	
	3-Hexen-1-ol	5284503	
	2,6,6-Trimethyl-2-cyclohexenone	88332	
	Camphor	2537	
	Acetophenone	7410	
	(cis)-Verbenol	164888	
	Borneol	64685	
	Verbenone	29025	
	2-Phenylethanol	6054	

	Ledol	92812	
	Eugenol	3314	
	4-Hydroxy-3-methylacetophenone	70135	
	Tricyclene	79035	[206]
	α-Thujene	17868	
	α-Pinene	6654	
	Camphene	6616	
	Sabinene	18818	
	β-Pinene	440967	
	α-Phellandrene<br>α -Terpinene	11142<br>7462	
	p-Cymene	7463	
	Cyclohexanone, 2,2,6-trimethyl	12600930	
	γ-Terpinene	7461	
	Terpinolene	11463	
	Sabinaketone	92784	
	α -Campholene aldehyde	1252759	
	Camphore	230921	
	Borneol	64685	
	Terpineol-4	11230	

	P-Cymen-8-ol	14529	
	α-terpineol	17100	
	1-Methyladamantane	136607	
	Verbenone	29025	
	l-Bornyl acetate	93009	
	p-Mentha-1,4-dien-7-ol	519966	
	(+)-2-Carene	78249	
	α-Cubebene	442359	
	1S, Cis-Calamenene	6429077	
	δ-Cadinene	441005	
	Hexadecanoic acid	985	
	Diethyl phthalate	6781	
	Viridiflorol	11996452	
	Cyercene	6442290	
	Bis (2-ethylhexyl) phthalate	8343	
	tricyclene	79035	[207]
	alpha-thujene	17868	
	alpha-pinene	6654	
	camphene	6616	
	sabinene	18818	

	beta-pinene	14896	
	alpha-phellandrene	7460	
	alpha-terpinene	7462	
	p-cymene	7463	
	beta-phellandrene	11142	
	2,2,6-trimethylcyclohexanone	17000	
	2,6,6-trimethylcyclohex-2-enone	85661508	
	p-cymenene	62385	
	alpha-pinene oxide	91508	
	p-mentha-1,3,8-triene	176983	
	cis-rose oxide	1712087	
	beta-thujone	91456	
	beta-fenchol	6973643	
	alpha-campholenal	1252759	
	trans-rose oxide	7093102	
	camphor	2537	
	camphene hydrate	101680	
	isoborneol	6321405	
	pinocarvone	121719	
	borneol	64685	

	terpinen-4-ol	11230	
	a-terpineol	17100	
	myrtenol	10582	
	verbenone	29025	
	trans-carveol	94221	
	cuminaldehyde	326	
	carvone	7439	
	geraniol	637566	
	bornyl acetate	93009	
	thymol	6989	
	carvacrol	10364	
	cumin alcohol	325	
	phenylpropionic acid	107	
	alpha-cubebene<br>eugenol	442359<br>3314	
	alpha-ylangene	442409	
	alpha-copaene	19725	
	beta-cubebene	93081	
	isoitalicene	10987385	
	alpha-gurjunene	15560276	

	beta-caryophyllene	5281515	
	beta-gurjunene	6450812	
	alpha-patchoulene	521710	
	allo-aromadendrene	42608158	
	gamma-muurolene	12313020	
	valencene	9855795	
	ledene	10910653	
	alpha-muurolene	12306047	
	delta-cadinene	441005	
	alpha-calacorene	12302243	
	ledol	92812	
	spathulenol	92231	
	caryophyllene oxide	1742210	
	viridiflorol	11996452	
	guaiol	227829	
	1-epi-cubenol	519857	
	beta-eudesmol	91457	
	cadalene<br>beta-bisabolol	10225<br>12300146	
	octadecanol	8221	

	octadecanal	12533	
	Tridecane, 3-methylene-	519705	[208]
	Bornyl acetate	93009	
	2,2-Dimethyl-3-vinyl-bicyclo[2.2.1]heptane	572691	
	.alpha.-Cubebene	86609	
	Cyclohexane, 1,2,3-trimethyl-	15507	
	Cyclopropanemethanol, .alpha.,2-dimethyl-2-(4-methyl-3-pentenyl)-	549714	
	Bicyclo[3.1.1]heptan-3-one, 6,6-dimethyl-2-(2-methylpropyl)-	549953	
	Bicyclo[4.4.0]dec-1-ene, 2-isopropyl-5-methyl-9-methylene-	595137	
	Benzo[h]quinoline, 2,3,4-trimethyl-	613551	
	Bicyclo[7.2.0]undec-4-ene, 4,11,11-trimethyl-8-methylene	5452236	
	Ledol	92812	
	3-Cyclohexene-1-carboxaldehyde, 4-methyl-	111012	
	Longifolene	289151	
	Cadinene	3032853	
	Cadinol	6428423	
	Amorphene	12306046	
	Cycloheptyl isopropylphosphonofluoridate	581200	
	Laminitol	244581	

	Benzenamine, 2,5-dihydromethyl-	592756	
	Longifolenaldehyde	565584	
	Bicyclo[3.1.1]heptane, 2,6,6-trimethyl (1a, 2b, 5a	17755566	
	3-(3-Hydroxybicyclo[2.2.1]hept-2-ylidene)-2-methylpropionic acid, methyl ester	5373146	
	n-Hexadecanoic acid	985	
	Hexadecanoic acid, ethyl ester	12366	
	Heneicosane	12403	
	Phytol	5280435	
	9,12-Octadecadienoic acid, ethyl ester	5365672	
	9,12,15-Octadecatrienoic acid, ethyl ester, (Z,Z,Z)-	5367460	
	Octadecanoic acid, ethyl ester	8122	
	6-Octen-1-ol, 3,7-dimethyl-, acetate	9017	
	Methyl 18-methylnonadecanoate	530340	
	Heptadecanoic acid	10465	
	Heptadecanoic acid, ethyl ester	26397	
	2,6,10-Dodecatrien-1-ol, 3,7,11-trimethyl-	445070	
	Bicyclo[2.2.1]heptane, 2,2,3-trimethyl-, endo-	296894	
	1,10-Dimethyl-2-methylene-trans-decalin	572965	
	Nonadecanoic acid, ethyl ester	29008	
	Octacosane	12408	

	Triacontyl acetate	3084839	
80. Citrus limon	alpha-Humulene	5281520	[209]
	beta-Caryophyllene	5281515	
	methylene-1,6,10-dodecatriene	5281517	
	beta-Phellandrene	11142	
	Limonene-1,2-epoxide	441245	
	4-Ethylguaiacol	62465	
	2-Methoxy-4-vinylphenol	332	
	1-Furfurylpyrrole	15037	
	2-Propanamine	6363	
	(+)-Dihydrocarvone	24473	
	Hydroxyacetone	8299	
	L(-)-Carvone	439570	
	D(+)-Carvone	16724	
	Piperiton	107561	
	beta-Lonone	5282108	
	beta-Ionone	638014	
	alpha-Ionone	5282108	
	4-Methylaceto phenone	102059259	
	(E,E)-3,5-Octadien-2-one	5352876	

	6-Methylhept-5-en-one<br>3-Hydroxy-2,3-dihydromaltol	9862<br>119838	
	1r-.alpha.-Pinene	12172380	
	1s-.beta.-Pinene	440967	
	Sabinene	18818	
	1S)-(-)-(-)-Pinene	440967	
	alpha-Pinene	6654	
	beta-Pinene	14896	
	beta-Myrcene	31253	
	D-Limonene	440917	
	Z)-beta-Ocimene	5320250	
	Terpinolene	11463	
	Elemol	92138	
	Phytol	5280435	
	trans-Nerolidol	5284507	
	(+)-beta-Citronellol	101977	
	L-alpha-Terpineol	443162	
	2-heptanol	10976	
	(±)-trans-4-Thujanol	6326181	
	cis-Linaloloxide	529304	

	Dodecanal	8194	
	Nonanal	31289	
	Citronellal	7794	
	Citral	638011	
	Undecanal	8186	
	Octanal	454	
	Decanal	8175	
	Perillaldehyde	16441	
	(E)-2-Decenal	5283345	
	L-Perillaldehyde	2724159	
	alpha-Sinensal	5281534	
	Tridecanal	25311	
	Benzaldehyde	240	
	+)-Citronellal	443157	
	Acetic acid	176	
	Tridecanoic	12530	
	n-Decanoic	2969	
	Thymol	6989	
	Carvacrol	10364	
	Esters	2337	

	Linalyl acetate<br>Methyl	8294<br>4173	
	benzoate	242	
	Ethyl caprate	8048	
	Octyl acetate	8164	
	Geranyl acetate	1549026	
	Decyl acetate	8167	
	1-Decanol	8174	
	acetate	175	
	Methyl caprate	8050	
	Alcohols	17072	
	Terpinen-4-ol	11230	
	1-Octanol	957	
	Linalool	6549	
	Nerolidol	5284507	
	cistrans	91127	
	p-Menth-1-	442501	
	en-8-ol	8748	
	Nerol	643820	
	(Z)-Carveol	330573	

	(E)-Carveol	94221	
	α-Pinene	6654	[210]
	Sabinene	18818	
	cis-β-Ocimene	5320250	
	d-Limonene	440917	
	γ-Terpinene	7461	
	Terpinolene	11463	
	Linalool	6549	
	IsoPulegol	170833	
	Isothujol	10550 0	
	α-Terpineol	17100	
	cis-Geraniol	643820	
	Geranyl acetate	1549026	
	Geranial	638011	
	β-Elemene	6918391	
	β-Caryophyllene	1742210	
	α-Bergamotene	6429302	
	Valencene	9855795	

	α-Farnesene(isomer)	5281516	
	β-Pinene	6654	
	aldrin	12310947	[211]
	dicofol	8268	
	dieldrin	969491	
	dimethoate	3082	
	methyl parathion	4130	
	methyl chlorpyrifos	21803	
	chlordane	5993	
	methidathion	13709	
	beta- pinene	440967	[212]
	Limonene	22311	
	alpha-terpinene	7462	
	Cis-ocimine		
	Limonene	22311	
	Perillaldehyde	16441	
	Myrtanol	521314	
	Geranial	638011	
	Citronellic acid	10402	

	Thymol<br>Nerolic acid	6989<br>5312583	
	Neryl acetate	549025	
	Geranyl acetate	1549026<br>_	
	Geranyl	1549026	
	propionate	104745	
	beta- Selinene	442393	
	Caryophyllene	5281515	
	oxide	190217	
	delta- Cadinene	441005	
	Spathulenol	92231	
	Ledol	92812	
	Humulene epoxide	8209	
	Tetradecanol	8209	
	Hexadecanoic acid	985	
81. Citrus Xaurantium	α-Thujene	17868	[213]
	Heptanol	8129	
	Sabinene	18818	
	Myrcene	31253	

	Octanal	454	
	Limonene	22311	
	Benzyl formate	7708	
	Linalool	6549	
	Nonanal	31289	
	trans-p-Mentha-2,8-dien-1-ol	12618691	
	Nonanol	8914	
	α-Terpineol	17100	
	Decanal	8175	
	Octanol acetate	8164	
	Nerol	643820	
	Citronellyl formate	7778	
	2-Adamantanone	64151	
	Myrtenyl acetate	61262	
	trans-Carvyl acetate	81505	
	Z-β-Damascenone	62775	
	Linalool isobutanoate	6532	
	Daucene	177773	
	Hexenyl 2-methyl-2-pentenoate	20839647	
	(E)-β-Caryophyllene	5281515	

	α-trans-Bergamotene	6429302	

References:

1. Song, B., et al., *Geographic Differentiation of Essential Oil from Rhizome of Cultivated Atractylodes lancea by Using GC-MS and Chemical Pattern Recognition Analysis.* Molecules, 2023. **28**(5).
2. Liu, Q., et al., *Differentiation of essential oils in Atractylodes lancea and Atractylodes koreana by gas chromatography with mass spectrometry.* J Sep Sci, 2016. **39**(24): p. 4773-4780.
3. Zhang, Y.Y., et al., *Preparation, characterization, and bioactivity evaluation of oligosaccharides from Atractylodes lancea (Thunb.) DC.* Carbohydr Polym, 2022. **277**: p. 118854.
4. Guo, F.-Q., et al., *Comparison of the volatile compounds of Atractylodes medicinal plants by headspace solid-phase microextraction-gas chromatography–mass spectrometry.* Analytica Chimica Acta, 2006. **570**(1): p. 73-78.
5. Bolade, O.P., et al., *Dataset on phytochemical screening, FTIR and GC-MS characterisation of Azadirachta indica and Cymbopogon citratus as reducing and stabilising agents for nanoparticles synthesis.* Data Brief, 2018. **20**: p. 917-926.
6. Mir Najib Ullah, S.N., et al., *Detection of phytoconstituents present in Azadirachta indica L. seeds extract by GC-MS analysis.* Journal of the Indian Chemical Society, 2022. **99**(11).
7. Kaur, S., et al., *Antimicrobial and Anti-Inflammatory Activity of Low-Energy Assisted Nanohydrogel of Azadirachta indica Oil.* Gels, 2022. **8**(7).
8. Murthy, H.N., et al., *Phytochemicals and Biological Activity of Desert Date (Balanites aegyptiaca (L.) Delile).* Plants (Basel), 2020. **10**(1).
9. Al Ashaal, H.A., et al., *Phytochemical investigation and medicinal evaluation of fixed oil of Balanites aegyptiaca fruits (Balantiaceae).* J Ethnopharmacol, 2010. **127**(2): p. 495-501.
10. Dawidar, A.E., et al., *Molluscicidal activity of Balanites aegyptiaca against Monacha cartusiana.* Pharm Biol, 2012. **50**(10): p. 1326-9.
11. Jaheed, E., et al., *Evaluation of the curative effect of Balanites aegyptiaca fruits ethanolic extract on Haemonchosis experimentally induced in Egyptian Baladi goats: phytoanalytical, parasitological and hematological studies.* J Parasit Dis, 2019. **43**(4): p. 638-650.
12. Batiha, G.E., et al., *Phytochemical Screening and Antiprotozoal Effects of the Methanolic Berberis vulgaris and Acetonic Rhus coriaria Extracts.* Molecules, 2020. **25**(3).
13. Hosseini Hashemi, S.K., et al., *Identification of Wood and Bark Extractives in Indigenous Barberry (Berberis vulgaris).* Lignocellulose, 2016. **5**: p. 77-83.
14. El-Zahar, K.M., et al., *Antioxidant, Antibacterial, and Antifungal Activities of the Ethanolic Extract Obtained from Berberis vulgaris Roots and Leaves.* Molecules, 2022. **27**(18).
15. Xuan, T.D. and T.D. Khanh, *Chemistry and pharmacology of Bidens pilosa: an overview.* J Pharm Investig, 2016. **46**(2): p. 91-132.
16. Vukovic, N., et al., *Chemical Composition of the Essential oil ofBougainvillea spectabilisfrom Montenegro.* Journal of Essential Oil Bearing Plants, 2013. **16**(2): p. 212-215.

17. B, S. and A. Fauzia, *Phytochemical Analysis of Bougainvillea spectabilis by Using GCMS.* International Journal of Zoological Investigations, 2021. **7**(2): p. 751-755.
18. Ferdous, A., et al., *The leaves of Bougainvillea spectabilis suppressed inflammation and nociception in vivo through the modulation of glutamatergic, cGMP, and ATP-sensitive K(+) channel pathways.* J Ethnopharmacol, 2020. **261**: p. 113148.
19. Sharma, A., P.K. Rai, and S. Prasad, *GC–MS detection and determination of major volatile compounds in Brassica juncea L. leaves and seeds.* Microchemical Journal, 2018. **138**: p. 488-493.
20. Zhao, D., J. Tang, and X. Ding, *Analysis of volatile components during potherb mustard (Brassica juncea, Coss.) pickle fermentation using SPME–GC-MS.* LWT - Food Science and Technology, 2007. **40**(3): p. 439-447.
21. Oyebode, O., et al., *In vitro and computational studies of the antioxidant and anti-diabetic properties of Bridelia ferruginea.* J Biomol Struct Dyn, 2022. **40**(9): p. 3989-4003.
22. Gurgur, E., et al., *Bridelia ferruginea dye-synthesized zinc oxide nanoparticles and its nitrogen and sulphur doped as a photoanode in photovoltaic cell fabrication.* Bulletin of Materials Science, 2022. **45**(3).
23. Afolayan, M., et al., *Chemical and biological studies on Bridelia ferruginea grown in Nigeria.* Nat Prod Res, 2019. **33**(2): p. 287-291.
24. Omolaso, B.O., et al., *Antidiarrheal activity of Bridelia ferruginea bark methanolic extract involves modulation ATPases in mice and inhibition of muscarinic acetylcholine receptor (M3) and prostaglandin E2 receptor 3 (EP(3)) in silico.* J Complement Integr Med, 2021.
25. Shahsavari, N., et al., *Antioxidant activity and chemical characterization of essential oil of Bunium persicum.* Plant Foods Hum Nutr, 2008. **63**(4): p. 183-8.
26. Miyazawa, M., et al., *Characteristic odor components of essential oil fromCaesalpinia decapetala.* Journal of Essential Oil Research, 2012. **24**(5): p. 441-446.
27. Wei, X.H., et al., *Chemical constituents of Caesalpinia decapetala (Roth) Alston.* Molecules, 2013. **18**(1): p. 1325-36.
28. Chalchat, J.C., R.P. Garry, and A. Michet, *Chemical composition of essential oil of Calendula officinalis L. (pot marigold).* Flavour and Fragrance Journal, 1991. **6**(3): p. 189-192.
29. Danielski, L., et al., *Marigold (Calendula officinalis L.) oleoresin: Solubility in SC-CO2 and composition profile.* Chemical Engineering and Processing: Process Intensification, 2007. **46**(2): p. 99-106.
30. Guo, Y., et al., *De novo transcriptome combined with spectrophotometry and gas chromatography-mass spectrometer (GC-MS) reveals differentially expressed genes during accumulation of secondary metabolites in purple-leaf tea (Camellia sinensis cv Hongyafoshou).* The Journal of Horticultural Science and Biotechnology, 2018. **94**(3): p. 349-367.
31. Lin, Y., et al., *Aroma Identification and Classification in 18 Kinds of Teas (Camellia sinensis) by Sensory Evaluation, HS-SPME-GC-IMS/GC x GC-MS, and Chemometrics.* Foods, 2023. **12**(13).
32. Hope, O., I.E. Bright, and A.I. Alagbonsi, *GC-MS biocomponents characterization and antibacterial potency of ethanolic crude extracts of Camellia sinensis.* SAGE Open Med, 2022. **10**: p. 20503121221116859.
33. Forero, M.D., C.E. Quijano, and J.A. Pino, *Volatile compounds of chile pepper (Capsicum annuumL. var.glabriusculum) at two ripening stages.* Flavour and Fragrance Journal, 2009. **24**(1): p. 25-30.
34. Lieb, V.M., et al., *GC-MS profiling, descriptive sensory analysis, and consumer acceptance of Costa Rican papaya (Carica papaya L.) fruit purees.* Food Chem, 2018. **248**: p. 238-246.

35. Sani, M.S.A., et al., *Effects of Coated Capillary Column, Derivatization, and Temperature Programming on the Identification of Carica papaya Seed Extract Composition Using GC/MS Analysis.* Journal of Analysis and Testing, 2020. **4**(1): p. 23-34.
36. Chua, L.Y.W., et al., *Characterisation of the Convective Hot-Air Drying and Vacuum Microwave Drying of Cassia alata: Antioxidant Activity, Essential Oil Volatile Composition and Quality Studies.* Molecules, 2019. **24**(8).
37. Agnaniet, H., et al., *Aromatic Plants of Tropical Central Africa. Part XLVI. Essential Oil Constituents ofCassia alata(L.) from Gabon.* Journal of Essential Oil Research, 2005. **17**(4): p. 410-412.
38. Saha, K., R.Z. Proma, and N. Khan, *Phytochemical Screening of Plant Extracts and GC-MS Analysis of n-Hexane Extract of the Leaves of Cassia alata Linn.* The Journal of Phytopharmacology, 2020. **9**(5): p. 342-347.
39. Meena, A.K., et al., *Evaluation for substitution of stem bark with small branches of Cassia fistula Linn for traditional medicinal uses: A comparative chemical profiling studies by HPLC, LC-MS, GC-MS.* Heliyon, 2022. **8**(8): p. e10251.
40. Tzakou, O., A. Loukis, and A. Said, *Essential Oil from the Flowers and Leaves ofCassia fistulaL.* Journal of Essential Oil Research, 2007. **19**(4): p. 360-361.
41. Ferdosi, M.F., A. Javaid, and I.H. Khan, *Phytochemical profile of n-hexane flower extract of Cassia fistula L.* Bangladesh Journal of Botany, 2022. **51**(2): p. 393-399.
42. Chen, Q., et al., *Metabolomics Characterization of Two Apocynaceae Plants, Catharanthus roseus and Vinca minor, Using GC-MS and LC-MS Methods in Combination.* Molecules, 2017. **22**(6).
43. Rani, J., et al., *Identification and Assessment of Therapeutic Phytoconstituents of Catharanthus roseus through GC-MS Analysis.* Separations, 2023. **10**(6).
44. De Pinho, P.G., et al., *Volatile composition of Catharanthus roseus (L.) G. Don using solid-phase microextraction and gas chromatography/mass spectrometry.* J Pharm Biomed Anal, 2009. **49**(3): p. 674-85.
45. Brun, G., et al., *Volatile Components of Catharanthus roseus (L.) G. Don (Apocynaceae).* Flavour and Fragrance Journal, 2001. **16**(2): p. 116-119.
46. Rivera Mondragón, A., et al., *Isolation and identification of flavonoids, saponins and two new flavolignans from Cecropia obtusifolia leaves collected in Panama.* Planta Medica International Open, 2017. **4**(S 01): p. Mo-PO-140.
47. Singh, R. and K.K. Chahal, *Cichorium intybus from India: GC-MS Profiling, Phenolic Content and in vitro Antioxidant Capacity of Sequential Soxhlet Extracted Roasted Roots.* Brazilian Archives of Biology and Technology, 2019. **62**.
48. Haghi, G., et al., *Chemical Composition of Essential Oil of Aerial Parts ofCichorium intybusL. from Iran.* Journal of Essential Oil Bearing Plants, 2012. **15**(2): p. 213-216.
49. Rustaiyan, A., et al., *Composition of the Essential Oils ofAnthemis HyalinaDC.,Achillea NobilisL. andCichorium intybusL. Three Asteraceae Herbs Growing Wild in Iran.* Journal of Essential Oil Bearing Plants, 2011. **14**(4): p. 472-480.
50. Mutlu, M., et al., *Comprehensive Metabolite Profiling of Cinnamon (Cinnamomum zeylanicum) Leaf Oil Using LC-HR/MS, GC/MS, and GC-FID: Determination of Antiglaucoma, Antioxidant, Anticholinergic, and Antidiabetic Profiles.* Life (Basel), 2023. **13**(1).
51. Jayaprakasha, G.K., L.J. Rao, and K.K. Sakariah, *Chemical Composition of Volatile Oil from Cinnamomum zeylanicum Buds.* Zeitschrift für Naturforschung C, 2002. **57**(11-12): p. 990-993.

[52] Stanojevic LP, Marjanovic-Balaban ZR, Kalaba VD, Stanojevic JS, Cvetkovic DJ. Chemical Composition, Antioxidant and Antimicrobial Activity of Chamomile Flowers Essential Oil (Matricaria chamomilla L.).

Journal of Essential Oil-Bearing Plants 2016;19:2017–28. https://doi.org/10.1080/0972060X.2016.1224689.

[53] Braca A, Siciliano T, D'Arrigo M, Germanò MP. Chemical composition and antimicrobial activity of Momordica charantia seed essential oil. Fitoterapia 2008;79:123–5. https://doi.org/10.1016/j.fitote.2007.11.002.

[54] Aini DM, Ratnasari BD, Hendry Z. Chemical constituents of ripe Momordica charantia by Gas Chromatography Mass Spectrometry (GCMS). INSOLOGI: Jurnal Sains Dan Teknologi 2022;1:189–94. https://doi.org/10.55123/insologi.v1i3.359.

[55] Fernando LN, Grn IU. Headspace - SPME analysis of volatiles of the ridge gourd (Luffa acutangula) and bitter gourd (Momordica charantia) flowers. Flavour Fragr J 2001;16:289–93. https://doi.org/10.1002/ffj.999.

[56] Ramalingam R, Palanisamy S, Mohanraj AK, Durisamy S, Rajasekaran N. Chemical Profiling of Momordica charantia L. Seed Essential Oil and Its Antimicrobial Activity. Journal of Essential Oil-Bearing Plants 2020;23:390–6. https://doi.org/10.1080/0972060X.2020.1741451.

[57] Nibret E, Wink M. Trypanocidal and antileukaemic effects of the essential oils of Hagenia abyssinica, Leonotis ocymifolia, Moringa stenopetala, and their main individual constituents. Phytomedicine 2010;17:911–20. https://doi.org/10.1016/j.phymed.2010.02.009.

[58] Manilal A, Sabu KR, Shewangizaw M, Aklilu A, Seid M, Merdikios B, et al. In vitro antibacterial activity of medicinal plants against biofilm-forming methicillin-resistant Staphylococcus aureus: efficacy of Moringa stenopetala and Rosmarinus officinalis extracts. Heliyon 2020;6. https://doi.org/10.1016/j.heliyon.2020.e03303.

[59] Dey P, Saha MR, Chowdhuri SR, Sen A, Sarkar MP, Haldar B, et al. Assessment of anti-diabetic activity of an ethnopharmacological plant Nerium oleander through alloxan induced diabetes in mice. J Ethnopharmacol 2015;161:128–37. https://doi.org/10.1016/j.jep.2014.12.012.

[60] Hase GJ, Deshmukh KK, Pokharkar RD, Gaje TR, Phatanagre ND. Phytochemical Studies on Nerium oleander L. Using GC-MS. International Journal of Pharmacognosy and Phytochemical Research 2017;9. https://doi.org/10.25258/phyto.v9i6.8195.

[61] Tadesse S, Messele B, Seyoum A, Mazumder A, Bucar F, Asres K. Essential Oil of *Otostegia integrifolia* Benth: Composition, Antimicrobial and Antioxidant Activities. Ethiopian Pharmaceutical Journal 2013;29. https://doi.org/10.4314/epj.v29i2.1.

[62] Buchbauer G, Jirovetz L. Volatile constituents of the essential oil of Passiflora incarnata L. Journal of Essential Oil Research 1992;4:329–34. https://doi.org/10.1080/10412905.1992.9698081.

[63] Kurkcuoglu M, Baser KHC, Vural M. Composition of the essential oil of Pastinaca sativa L. subsp. urens (Req. ex Godron) celak. Chem Nat Compd 2006;42:114–5. https://doi.org/10.1007/s10600-006-0053-2.

[64] Kviesis J, Kļimenkovs I, Arbidans L, Podjava A, Kļaviņš M, Liepiņš E. Evaluation of furanocoumarins from seeds of the wild parsnip (Pastinaca sativa L. s.l.). J Chromatogr B Analyt Technol Biomed Life Sci 2019;1105:54–66. https://doi.org/10.1016/j.jchromb.2018.12.012.

[65] Meresa A, Gemechu W, Basha H, Fekadu N, Teka F, Ashebir R, et al. American Journal of Advanced Drug Delivery American Journal of Advanced Drug Delivery http://www.imedpub.com/advanced-drug-delivery/ Herbal Medicines for the Management of Diabetic Mellitus in Ethiopia and Eretria including their Phytochemical Constituents. n.d.

[67] Elangovan M, Dhanarajan M, Elangovan I, Elangovan Research scholar M. DETERMINATION OF BIOACTIVE COMPOUNDS FROM THE PETROLEUM ETHER LEAF EXTRACT OF MORINGA OLEIFERA AND PHYLLANTHUS EMBLICA USING GC-MS ANALYSIS. Malliga Elangovan et al World Journal of Pharmaceutical Research World Journal of Pharmaceutical Research SJIF Impact Factor 5 2015;4:1284–98.

[68] Ethnicity and Scientific validation of West Bengal Amla (Phyllanthus emblica L.) with special reference to GC-MS screening n.d. https://doi.org/10.13140/RG.2.1.3922.3440.

[69] Farzaneh V, Gominho J, Pereira H, Carvalho IS. Screening of the Antioxidant and Enzyme Inhibition Potentials of Portuguese Pimpinella anisum L. Seeds by GC-MS. Food Anal Methods 2018;11:2645–56. https://doi.org/10.1007/s12161-018-1250-x.

[70] Derwich E, Manar A, Benziane Z, Boukir A. GC/MS Analysis and In vitro Antibacterial Activity of the Essential Oil Isolated from Leaf of Pistacia lentiscus Growing in Morocoo. World Appl Sci J 2010;8:1267–76.

[71] Piccolella S, Nocera P, Carillo P, Woodrow P, Greco V, Manti L, et al. An apolar Pistacia lentiscus L. leaf extract: GC-MS metabolic profiling and evaluation of cytotoxicity and apoptosis inducing effects on SH-SY5Y and SK-N-BE(2)C cell lines. Food and Chemical Toxicology 2016;95:64–74. https://doi.org/10.1016/j.fct.2016.06.028.

[72] Chaabani E, Abert Vian M, Bott R, Ginies C, Defoort C, Ksouri R, et al. Extraction of aromas from Pistacia lentiscus L. leaves using alternative solvents: COSMO-RS-assisted solvent screening and GC-MS metabolites profiling. Separation Science and Technology (Philadelphia) 2020;55:716–27. https://doi.org/10.1080/01496395.2019.1574821.

[73] V R, AR N. Phytochemical and chromatographic analysis of flavanoid fraction isolated from methanoloc extract of Pterocarpus marsupium. The Journal of Phytopharmacology 2022;11:79–88. https://doi.org/10.31254/phyto.2022.11205.

[74] Yadav S, Gupta RK, Nand P. Formulation and phytochemicals characterization of polyherbal (Tinospora cordifolia, Gymnema sylvestre, Pterocarpus marsupium and Acacia arabica) antidiabetic compressed tablet lozenges. ~ 244 ~ Journal of Pharmacognosy and Phytochemistry 2015;4:244–53.

[75] Lingappa Hugar A, Londonkar RL, Hugar AL. GC-MS profiling of bioactive components from aqueous extract of Pterocarpus marsupium. 2017.

[76] Aruna LH, Kanjikar AP, Londonkar RL. Bioactive compounds investigation from methanol bark extract of Pterocarpus marsupium using GC-MS analysis. International Journal of Pharmaceutical Quality Assurance 2017;8:104–10. https://doi.org/10.25258/ijpqa.v8i03.9571.

[77] El Ouariachi EM, Tomi P, Bouyanzer A, Hammouti B, Desjobert JM, Costa J, et al. Chemical composition and antioxidant activity of essential oils and solvent extracts of Ptychotis verticillata from Morocco. Food and Chemical Toxicology 2011;49:533–6. https://doi.org/10.1016/j.fct.2010.11.019.

[78] Bnouham M, Benalla W, Asehraou A, Berrabah M. Antibacterial activity of essential oil from <i>Ptychotis verticillata</i> Spatula DD - Peer Reviewed Journal on Complementary Medicine and Drug Discovery 2012;2:69. https://doi.org/10.5455/spatula.20120119070423.

[79] Li R, Yang JJ, Song XZ, Wang YF, Corlett RT, Xu YK, et al. Chemical composition and the cytotoxic, antimicrobial, and anti-inflammatory activities of the fruit peel essential oil from Spondias pinnata (anacardiaceae) in Xishuangbanna, Southwest China. Molecules 2020;25. https://doi.org/10.3390/molecules25020343.

[79] Sameh S, Al-Sayed E, Labib RM, Singab ANB. Comparative metabolic profiling of essential oils from Spondias pinnata (Linn. F.)Kurz and characterization of their antibacterial activities. Ind Crops Prod 2019;137:468–74. https://doi.org/10.1016/j.indcrop.2019.05.060.

[80] Rohloff J, Mordal R, Dragland S. Chemotypical Variation of Tansy (Tanacetum vulgare L.) from 40 Different Locations in Norway. J Agric Food Chem 2004;52:1742–8. https://doi.org/10.1021/jf0352430.

[81] Sinha A. PHYTO-CHEMICAL STUDIES OF METHANOL EXTRACTS OF TINOSPORA CORDIFOLIA STEM BY GC-MS. World J Pharm Res 2017:1319–26. https://doi.org/10.20959/wjpr20174-8205.

[82] Rathi D, Balasubramanian PL. Phytochemical Compound Analysis of Tinospora Cordifolia By GC-MS Method. vol. 5. 2018.

[83] Akbari S, Abdurahman NH, Yunus RM, Alara OR, Abayomi OO. Extraction, characterization and antioxidant activity of fenugreek (Trigonella-Foenum Graecum) seed oil. Mater Sci Energy Technol 2019;2:349–55. https://doi.org/10.1016/j.mset.2018.12.001.

[84] Ashraf SA, Khan MA, Awadelkareem AM, Tajuddin S, Ahmad MF, Hussain T. GC-MS analysis of commercially available allium sativum and trigonella foenum-graecum essential oils and their antimicrobial activities. J Pure Appl Microbiol 2019;13:2545–52. https://doi.org/10.22207/JPAM.13.4.69.

[85] Keskes H, Belhadj S, Jlail L, El Feki A, Sayadi S, Allouche N. LC–MS–MS and GC–MS analyses of biologically active extracts of Tunisian Fenugreek (Trigonella foenum-graecum L.) Seeds. Journal of Food Measurement and Characterization 2018;12:209–20. https://doi.org/10.1007/s11694-017-9632-0.

[86] P. V. and A. K. Ajay, ‘Screening of potential GCMS derived antimigraine compound from the leaves of Abrus precatorius Linn to target “calcitonin gene related peptide” receptor using in silico analysis’, Food Science and Human Wellness, vol. 8, no. 1, pp. 34–39, Mar. 2019, doi: 10.1016/j.fshw.2019.01.001.

[87] K. Pavithra, V. V. S. Uddandrao, S. Mathavan, N. Gobeeswaran, S. Vadivukkarasi, and S. Ganapathy, ‘Identification of bioactive factors from Abrus precatorius by GC-MS, NMR and evaluation of its antioxidant activity’, in Materials Today: Proceedings, Elsevier Ltd, 2019, pp. 3518–3521. doi: 10.1016/j.matpr.2019.05.417.

[88] F. Al-Qurainy et al., ‘Genome Estimation and Phytochemical Compound Identification in the Leaves and Callus of Abrus precatorius: A Locally Endangered Plant from the Flora of Saudi Arabia’, Plants, vol. 11, no. 4, Feb. 2022, doi: 10.3390/plants11040567.

[89] O. Iyekowa and O. V. Ndubuisi, 'Phytochemical Constituents, Haematological Activities and GC-MS Analysis of Isolated Oil of Rosary Pea (Abrus precatorius) in Wistar Rat Fed with High Lipid Diet', Tanzania Journal of Science, vol. 48, no. 4, pp. 863–874, Dec. 2022, doi: 10.4314/tjs.v48i4.13.

[90] S. Yadav, R. K. Gupta, and P. Nand, 'Formulation and phytochemicals characterization of polyherbal (Tinospora cordifolia, Gymnema sylvestre, Pterocarpus marsupium and Acacia arabica) antidiabetic compressed tablet lozenges', ~ 244 ~ Journal of Pharmacognosy and Phytochemistry, vol. 4, no. 2, pp. 244–253, 2015.

[91] R. Ali et al., 'Antileishmanial Evaluation of Bark Methanolic Extract of Acacia nilotica: In Vitro and in Silico Studies', ACS Omega, vol. 6, no. 12, pp. 8548–8560, Mar. 2021, doi: 10.1021/acsomega.1c00366.

[92] R. Kumari, R. C. Mishra, R. Sheoran, and J. P. Yadav, 'Fractionation of antimicrobial compounds from acacia nilotica twig extract against oral pathogens', Biointerface Res Appl Chem, vol. 10, no. 6, pp. 7097–7105, Dec. 2020, doi: 10.33263/BRIAC106.70977105.

[93] A. Abdalla and A. A. Ahmed, 'Proximate analysis, mineral contents, and GC-MS analysis of leaves, twigs, and thorns of Acacia Etbaica Schweinf', ~ 8 ~ Journal of Medicinal Plants Studies, vol. 11, no. 2, pp. 8–11, 2023, [Online]. Available: https://www.plantsjournal.com

[94] S. Kurian, L. Joseph, and V. S. Josekumar, 'Phytochemical evaluation, GC-MS analysis and antimicrobial activity of the leaves petiole of Artocarpus heterophyllus Lam', Asian J Pharm Pharmacol, vol. 4, no. 3, pp. 280–287, 2018, doi: 10.31024/ajpp.2018.4.3.7.

[95] I. Yani, B. Tambunan, E. Siringo-Ringo, M. Julianti Butar-Butar, and K. Gurning, 'GC-MS analysis of bioactive compounds and antibacterial activity of nangka leaves (Artocarpus heterophyllus Lam)', doi: 10.3897/pharma.

[96] B. O. Ajiboye et al., 'Inhibitory effect on key enzymes relevant to acute type-2 diabetes and antioxidative activity of ethanolic extract of Artocarpus heterophyllus stem bark', Journal of Acute Disease, vol. 5, no. 5, pp. 423–429, Sep. 2016, doi: 10.1016/j.joad.2016.08.011.

[97] S. Mickymaray, M. S. Al Aboody, P. K. Rath, P. Annamalai, and T. Nooruddin, 'Screening and antibacterial efficacy of selected Indian medicinal plants', Asian Pac J Trop Biomed, vol. 6, no. 3, pp. 185–191, 2016, doi: 10.1016/j.apjtb.2015.12.005.

[98] Y. Mariswamy, W. E. Gnanaraj, J. M. Antonisamy, A. A. Adaikalam, and V. Jamesraj, 'Please cite this article in press as Yamunadevi Mariswamy et.al. GC-MS Studies on Methanolic Extracts of Aerva lanata L', Indo American Journal of Pharmaceutical Research, vol. 2013, no. 3, p. 3, 2013, [Online]. Available: http://www.iajpr.com/index.php/en/www.iajpr.com

[99] A. K. Meena *et al.*, "Evolution of Pharmacological activity with Molecular Docking of active constituents present in roots and small branches of Aegle Marmelos: A comparative study using HPLC, GC–MS, LC-MS," *Phytomedicine Plus*, vol. 2, no. 1, Feb. 2022, doi: 10.1016/j.phyplu.2021.100210.

[100] W. Ahmad *et al.*, "Aegle marmelos leaf extract phytochemical analysis, cytotoxicity, in vitro antioxidant and antidiabetic activities," *Plants*, vol. 10, no. 12, Dec. 2021, doi: 10.3390/plants10122573.

[101] D. A. Ukeh, M. A. Birkett, J. A. Pickett, A. S. Bowman, and A. Jennifer Mordue, “Repellent activity of alligator pepper, Aframomum melegueta, and ginger, Zingiber officinale, against the maize weevil, Sitophilus zeamais,” *Phytochemistry*, vol. 70, no. 6, pp. 751–758, Apr. 2009, doi: 10.1016/j.phytochem.2009.03.012.

[102] A. Cs, A. Okechukwu, N. Ci, and A. Chukwuma, “Evaluation of the phytochemical and GC-MS analysis of the aqueous seed extract of Aframomum melegueta,” *~ 101 ~ Journal of Pharmacognosy and Phytochemistry*, vol. 6, no. 2, pp. 101–104, 2017.

[103] E. S. Madivoli, P. G. Kareru, E. G. Maina, A. O. Nyabola, S. I. Wanakai, and J. O. Nyang’au, “Biosynthesis of iron nanoparticles using Ageratum conyzoides extracts, their antimicrobial and photocatalytic activity,” *SN Appl Sci*, vol. 1, no. 5, May 2019, doi: 10.1007/s42452-019-0511-7.

[104] S. Bañaras, A. Javaid, and I. H. Khan, “Bioassays Guided Fractionation of Ageratum conyzoides Extract for the Identification of Natural Antifungal Compounds against Macrophomina phaseolina,” *Int J Agric Biol*, vol. 25, no. 4, pp. 761–767, 2021, doi: 10.17957/IJAB/15.1727.

[105] M. Abubakar and R. Majinda, “GC-MS Analysis and Preliminary Antimicrobial Activity of Albizia adianthifolia (Schumach) and Pterocarpus angolensis (DC),” *Medicines*, vol. 3, no. 1, p. 3, Jan. 2016, doi: 10.3390/medicines3010003.

[106] O. O. Aiyelaagbe, A. E. Oyewole, and A. I. Oladosu, “The chemistry and cytotoxicity effects of essential oils of leaves and flowers of albizia lebbeck (L.) benth (fabaceae) growing in Nigeria,” *Journal of Essential Oil-Bearing Plants*, vol. 13, no. 5, pp. 644–649, 2010, doi: 10.1080/0972060X.2010.10643875.

[107] A. Millet, E. Lamy, D. Jonas, F. Stintzing, V. Mersch-Sundermann, and I. Merfort, “Fermentation enhances the biological activity of Allium cepa bulb extracts,” *J Agric Food Chem*, vol. 60, no. 9, pp. 2148–2156, Mar. 2012, doi: 10.1021/jf2041643.

[108] M. S. Islam, H. Choi, and D. T. Loots, “Effects of dietary onion (Allium cepa L.) in a high-fat diet streptozotocin-induced diabetes rodent model,” *Ann Nutr Metab*, vol. 53, no. 1, pp. 6–12, Oct. 2008, doi: 10.1159/000152868.

[109] N. C. J. P. Lekshmi, S. Viveka, M. B. Viswanathan, G. Manivannan, and T. M. Shobi, “GC-MS Characterization of Volatile Odorous Compounds in Allium Cepa.”

[110] T. I. Adelusi *et al.*, “GCMS fingerprints and phenolic extracts of Allium sativum inhibit key enzymes associated with type 2 diabetes,” *J Taibah Univ Med Sci*, vol. 18, no. 2, pp. 337–347, Apr. 2023, doi: 10.1016/j.jtumed.2022.09.011.

[111] N. C. J. P. Lekshmi, S. Viveka, M. B. Viswanathan, and T. M. Shobi, “GC-MS CHARACTERIZATION OF ORGANIC SULPHUR COMPOUNDS AND OTHER VOLATILE ODOROUS COMPOUNDS FROM ALLIUM SATIVUM,” 2015. [Online]. Available: www.massbank.jp/jsp/Dispatcher.jsp

[112] S. Dziri, H. Casabianca, B. Hanchi, and K. Hosni, “Composition of garlic essential oil (Allium sativum L.) as influenced by drying method,” *Journal of Essential Oil Research*, vol. 26, no. 2, pp. 91–96, Mar. 2014, doi: 10.1080/10412905.2013.868329.

[113] P. T. V Lakshmi and P. Rajalakshmi, “IDENTIFICATION OF PHYTO-COMPONENTS AND ITS BIOLOGICAL ACTIVITIES OF ALOE VERA THROUGH THE GAS CHROMATOGRAPHY-MASS SPECTROMETRY,” 2011. [Online]. Available: http://www.irjponline.com

[114] P. Kushwaha, M. Shrivastava, D. Pandey, K. Verma, and L. Dwivedi, “The phytochemical profiling of Aloe vera through GC-MS and compounds activity validation at NCBI for industrial value addition of the plant,” *South Asian Journal of Experimental Biology*, vol. 13, no. 1, pp. 20–32, Mar. 2023, doi: 10.38150/sajeb.13(1).p20-32.

[115] I. Hussain, S. Khan, M. I. Khan, I. U. Rehman, and M. Ahmed, “Investigation of fatty acid composition of ammi majus seed oil by gas chromatography mass spectrometry,” *Journal of the Chinese Chemical Society*, vol. 59, no. 5, pp. 655–658, May 2012, doi: 10.1002/jccs.201100477.

[116] P. S. Kaboodi, A. A. Moghadamnia, D. Bakhshi, and A. A. Sefidgar, “A study of phytochemical properties of various extracts of Ammi majus fruit using GC-MS technique,” 2017.

[117] J. G. S. Maia, E. H. A. Andrade, and M. D. G. B. Zoghbi, “Volatile Constituents of the Leaves, Fruits and Flowers of Cashew (Anacardium occidentale L.),” *Journal of Food Composition and Analysis*, vol. 13, no. 3, pp. 227–232, Jun. 2000, doi: 10.1006/jfca.2000.0894.

[118] J. K. Adesanwo, S. B. Ogundele, D. A. Akinpelu, and A. G. McDonald, “Chemical Analyses, Antimicrobial and Antioxidant Activities of Extracts from Cola nitida Seed,” J. Explor. Res. Pharmacol., vol. 2, no. 3, pp. 67–77, 2017, doi: 10.14218/jerp.2017.00015.

[119] D. Yin, R. Xue, Y. Li, M. Zhu, and D. Li, “Valorization of Coptis chinensis extraction residue via slow pyrolysis for the production of bioactive wood vinegar,” Biomass Convers. Biorefinery, no. 0123456789, 2023, doi: 10.1007/s13399-023-03890-x.

[120] X. Yue et al., “Molecules and functions of Cornus officinalis bark volatiles,” Emirates J. Food Agric., vol. 30, no. 10, pp. 828–838, 2018, doi: 10.9755/ejfa.2018.v30.i10.1836.

[121] A. Meresa et al., “Correspondence Asfaw Meresa Ethno medicinal uses, phytochemistry and anti-malarial effect of Croton macrostachyus (Bisana): A review,” ~ 79 ~ J. Med. Plants Stud., vol. 7, no. 2, pp. 79–88, 2019.

[122] J. U. Chowdhury, N. C. Nandi, M. Nazrul, I. Bhuiyan, and M. Hosnay Mobarok, “Essential Oil Constituents of The Rhizomes of Two Types of Curcuma longa of Bangladesh,” Bangladesh J. Sci. Ind. Res, vol. 43, no. 2, pp. 259–266, 2008, [Online]. Available: www.babglajol.info

[123] S. Kaushik, S. Kaushik, R. Kumar, L. Dar, and J. P. Yadav, “In-vitro and in silico activity of Cyamopsis tetragonoloba (Gaur) L. supercritical extract against the dengue-2 virus,” VirusDisease, vol. 31, no. 4, pp. 470–478, 2020, doi: 10.1007/s13337-020-00624-9.

[124] A. Abdel-Moneim, O. M. Ahmed, S. M. Abd El-Twab, M. Y. Zaky, and L. N. Bakry, “Prophylactic effects of Cynara scolymus L. leaf and flower hydroethanolic extracts against diethylnitrosamine/acetylaminoflourene-induced lung cancer in Wistar rats,” Environ. Sci. Pollut. Res., vol. 28, no. 32, pp. 43515–43527, 2021, doi: 10.1007/s11356-021-13391-x.

[125] I. Ali et al., “Chemical analysis of Dalbergia sissoo (Roxb.) pod oil by (GC-MS) / GC-FID and evaluation of antioxidant potential,” Pak. J. Pharm. Sci., vol. 32, no. 5, pp. 2175–2181, 2019.

[126] A. El Bazaoui, M. Ahmed Bellimam, and A. Soulaymani, “Nine new tropane alkaloids from Datura stramonium L. identified by GC/MS,” Fitoterapia, vol. 82, no. 2, pp. 193–197, 2011, doi: 10.1016/j.fitote.2010.09.010.

[127] C. Acharya, “Ethnicity and Scientific validation of West Bengal Amla (Phyllanthus emblica L.) with special reference to GC-MS screening”, doi: 10.13140/RG.2.1.3922.3440.

[128] A. Henmi, M. Shoji, M. Nomura, and T. Inoue, “Fatty acid composition and applications of eriobotrya japonica seed oil,” J. Oleo Sci., vol. 68, no. 7, pp. 599–606, 2019, doi: 10.5650/jos.ess18178.

[129] R. Yusnaini et al., “Ethanolic Extract from Limonia acidissima L. Fruit Attenuates Serum Uric Acid Level via URAT1 in Potassium Oxonate-Induced Hyperuricemic Rats,” Pharmaceuticals, vol. 16, no. 3, pp. 1–13, 2023, doi: 10.3390/ph16030419.

[130] N. Shahsavari, M. Barzegar, M.A. Sahari, H. Naghdibadi, Antioxidant activity and chemical characterization of essential oil of Bunium persicum, Plant Foods for Human Nutrition. 63 (2008) 183–188. https://doi.org/10.1007/s11130-008-0091-y.

[131] F. Sharififar, N. Yassa, P.J. Pharm Sci, V. Mozaffarian, Bioactivity of major components from the seeds of Bunium Persicum (Boiss) 2010. https://www.researchgate.net/publication/44690953.

[132] T. Sekine, M. Sugano, A. Majid, Y. Fujii, Antifungal effects of volatile compounds from black zira (Bunium persicum) and other spices and herbs, J Chem Ecol. 33 (2007) 2123–2132. https://doi.org/10.1007/s10886-007-9374-2.

[133] A. Jamshidi, M. Azizi, M. Azizzadeh, M. Hashemi, Modeling the growth of Staphylococcus aureus as affected by black zira (Bunium persicum) essential oil, temperature, pH and inoculum levels, 2014. https://www.researchgate.net/publication/270659621.

[134] M. Miyazawa, T. Nagata, H. Nakahashi, T. Takahashi, Characteristic odor components of essential oil from Caesalpinia decapetala, Journal of Essential Oil Research. 24 (2012) 441–446. https://doi.org/10.1080/10412905.2012.703475.

[135] C. Martel, N. Rojas, M. Marín, R. Avilés, E. Neira, J. Santiago, Caesalpinia spinosa (Caesalpiniaceae) leaves: anatomy, histochemistry, and secondary metabolites, Revista Brasileira de Botanica. 37 (2014) 167–174. https://doi.org/10.1007/s40415-014-0059-0.

[136] R. Dhyea, A. Jalill, GC-MS Analysis of Calendula officinals and cytotoxicity effects of its flower crude extract on human epidermoid larynx carcinoma (HEP-2), 2014. www.wjpps.com.

[137] L. Danielski, L.M.A.S. Campos, L.F.V. Bresciani, H. Hense, R.A. Yunes, S.R.S. Ferreira, Marigold (Calendula officinalis L.) oleoresin: Solubility in SC-CO2 and composition profile, Chemical Engineering and Processing: Process Intensification. 46 (2007) 99–106. https://doi.org/10.1016/j.cep.2006.05.004.

[138] A. Raal, A. Orav, J. Nesterovitsch, K. Maidla, Analysis of Carotenoids, Flavonoids and Essential Oil of Calendula officinalis Cultivars Growing in Estonia, n.d.

[139] V. Kaškoniene, P. Kaškonas, M. Jalinskaite, A. Maruška, Chemical composition and chemometric analysis of variation in essential oils of Calendula officinalis L. during vegetation stages, Chromatographia. 73 (2011). https://doi.org/10.1007/s10337-011-1910-0.

[140] I. O. Caamal-Herrera, Tomás J. Madera-Santana, Tomás J. Madera-Santana, A. Azamar, Identification of volatile compounds in hydro-alcoholic extracts of Calendula officinalis L. flowers and Mimosae tenuiflorae bark using GC/MS, International Journal of Applied Research in Natural Products.

[141] O. Tamut, K.P. Singh, A.P. Raina, Namita, S. Panwar, P. Kumar, P.K. Verma, Composition and yield variation of essential oils from French marigold (Tagetes patula L.) genotypes using GC-MS, Indian Journal of Horticulture. 74 (2017) 97–102. https://doi.org/10.5958/0974-0112.2017.00022.6.

[142] A. Asghari, M.A.T. Ghanbary✉, M. Bakhshi, V. Babaeizad, Bioactive potential and GC-MS fingerprinting of extracts from endophytic fungi associated with seeds of some medicinal plants, Mycol Iran. 10 (2023) 55–67. https://doi.org/10.22043/MI.2023.360789.1242.

[143] Y. shuai Wang, M. zhe Fang, S. dao Zheng, J.G. Cho, T.H. Yi, Identification of Chinese green tea (Camellia sinensis) marker metabolites using GC/MS and UPLC-QTOF/MS, Food Sci Biotechnol. 30 (2021) 1293–1301. https://doi.org/10.1007/s10068-021-00970-4.

[144] C. ning Nie, X. xue Zhong, L. He, Y. Gao, X. Zhang, C. ming Wang, X. Du, Comparison of different aroma-active compounds of Sichuan Dark brick tea (Camellia sinensis) and Sichuan Fuzhuan brick tea using gas chromatography–mass spectrometry (GC–MS) and aroma descriptive profile tests, European Food Research and Technology. 245 (2019) 1963–1979. https://doi.org/10.1007/s00217-019-03304-1.

[145] S. Pradhan, R.C. Dubey, GC–MS analysis and molecular docking of bioactive compounds of Camellia sinensis and Camellia assamica, Arch Microbiol. 203 (2021) 2501–2510. https://doi.org/10.1007/s00203-021-02209-6.

[146] J. Lin, Y. Dai, Y.N. Guo, H.R. Xu, X.C. Wang, Volatile profile analysis and quality prediction of Longjing tea (Camellia sinensis) by HS-SPME/GC-MS, J Zhejiang Univ Sci B. 13 (2012) 972–980. https://doi.org/10.1631/jzus.B1200086.

[147] S.D. Lv, Y.S. Wu, Y.Z. Song, J.S. Zhou, M. Lian, C. Wang, L. Liu, Q.X. Meng, Multivariate Analysis Based on GC-MS Fingerprint and Volatile Composition for the Quality Evaluation of Pu-Erh Green Tea, Food Anal Methods. 8 (2015) 321–333. https://doi.org/10.1007/s12161-014-9900-0.

[148] Y.Q. Yang, H.X. Yin, H.B. Yuan, Y.W. Jiang, C.W. Dong, Y.L. Deng, Characterization of the volatile components in green tea by IRAE- HS- SPME/GC- MS combined with multivariate analysis, PLoS One. 13 (2018). https://doi.org/10.1371/journal.pone.0193393.

[149] H. Jasim, M. Altameme, GC-MS and FTIR analysis Phytocomponents on different parts of Capparis spinosa L. (Capparidaceae) in Iraq, (n.d.). www.jchps.com.

[150] R.O. Bakr, M.H. El Bishbishy, Profile of bioactive compounds of Capparis spinosa var.Aegyptiaca growing in Egypt, Revista Brasileira de Farmacognosia. 26 (2016) 514–520. https://doi.org/10.1016/j.bjp.2016.04.001.

[151] I. Rajhi, F. Hernandez-Ramos, M. Abderrabba, M.T. Ben Dhia, S. Ayadi, J. Labidi, Antioxidant, antifungal and phytochemical investigations of capparis spinosa L., Agriculture (Switzerland). 11 (2021). https://doi.org/10.3390/agriculture11101025.

[152] M.F.J. Al-Khafagi, D.Y. Mohammed, Study Antibacterial Activity of Crude Capparis spinosa L. Extracts Against Helicobacter pylori Infection and Determine Their Bioactive Compounds, Iraqi Journal of Science. 64 (2023) 503–512. https://doi.org/10.24996/ijs.2023.64.2.1.

[153] H.S. Sonbol, S.B. Hosawi, M.B. Hosawi, GC-MS analysis of the bioactive phytochemical compounds with anticancer activity in the Capparis cartilaginea fruit extracts, Journal of Advanced Pharmacy Education and Research. 13 (2023) 64–70. https://doi.org/10.51847/jggx9yy998.

[154] S. Reale, A. Biancolillo, C. Gasparrini, L. Di Martino, V. Di Cecco, A. Manzi, M. Di Santo, A.A. D'Archivio, Geographical discrimination of bell pepper (Capsicum annuum) spices by (HS)-SPME/GC-MS aroma profiling and chemometrics, Molecules. 26 (2021). https://doi.org/10.3390/molecules26206177.

[155] J.E. Lee, S.H. Jang, S.H. Hur, H.Y. Bang, I. kyung Bae, H.J. Kim, LC-MS/MS and GC-MS/MS cross-checking analysis method for 247 pesticide residues in sweet pepper (Capsicum annuum), Int J Food Prop. 24 (2021) 1758–1776. https://doi.org/10.1080/10942912.2021.1993251.

[156] A.C. Oulaï, K.M. Djè, K.P. Eba, A.A. Adima, E.J.P. Kouadio, Chemical composition, antioxidant and antimicrobial activities of Capsicum annuum var. annuum concentrated extract obtained by reverse osmosis, GSC Biological and Pharmaceutical Sciences. 5 (2018) 116–125. https://doi.org/10.30574/gscbps.2018.5.2.0123.

[157] A. V. Samrot, N. Shobana, R. Jenna, Antibacterial and Antioxidant Activity of Different Staged Ripened Fruit of Capsicum annuum and Its Green Synthesized Silver Nanoparticles, Bionanoscience. 8 (2018) 632–646. https://doi.org/10.1007/s12668-018-0521-8.

[158] M.S.A. Sani, J. Bakar, R.A. Rahman, F. Abas, Effects of Coated Capillary Column, Derivatization, and Temperature Programming on the Identification of Carica papaya Seed Extract Composition Using GC/MS Analysis, J Anal Test. 4 (2020) 23–34. https://doi.org/10.1007/s41664-020-00118-z.

[159] S.A. Ezekwe, P.C. Chikezie, GC-MS Analysis, Hypoglycemic Activity of Aqueous Root Extract of Carica papaya and Its Effects on Blood Lipid Profile and Hepatorenal Tissues Biomarkers of Diabetic Rats, J Diabetes Metab. 08 (2017). https://doi.org/10.4172/2155-6156.1000740.

[160] K.Y. Khaw, N.J.Y. Chear, S. Maran, K.Y. Yeong, Y.S. Ong, B.H. Goh, Butyrylcholinesterase inhibitory activity and GC-MS analysis of carica papaya leaves, Natural Product Sciences. 26 (2020) 165–170. https://doi.org/10.20307/nps.2020.26.2.165.

[161] J.O. Momoh, O.A. Damazio, O.M. Oyegbami, GC–MS Analysis and Antimalarial Activity of Methanolic Leaf Extract of Carica papaya against Plasmodium berghei NK65 Infection in Swiss Mice, Annu Res Rev Biol. (2020) 183–197. https://doi.org/10.9734/arrb/2020/v35i1230323.

[162] H.L. Al-Seadi, M.Z. Sabti, D.A. Taain, GC-MS Analysis of Papaya Leaf Extract (Carica Papaya L.), in: IOP Conf Ser Earth Environ Sci, IOP Publishing Ltd, 2021. https://doi.org/10.1088/1755-1315/910/1/012011.

[163] K. Saha, R.Z. Proma, N. Khan, Phytochemical Screening of Plant Extracts and GC-MS Analysis of n-Hexane Extract of the Leaves of Cassia alata Linn, The Journal of Phytopharmacology. 9 (2020) 342–347. https://doi.org/10.31254/phyto.2020.9509.

[164] O. Oluwasegun Victor, GC-MS Analysis of Phyto-components from the Leaves of Senna alata L, Journal of Plant Sciences (Science Publishing Group). 3 (2015) 133. https://doi.org/10.11648/j.jps.20150303.14.

[165] M.M.D. Mohammed, S.M. Mohamed, Phytochemical, cytotoxicity and antioxidant investigation of Cassia alata leaves growing in Egypt. Structural Elucidation of Natural Compounds: From Complexity to Simplicity View project, (n.d.). www.jipbs.com.

[166] I. Abubakar, A. Mann, J.T. Mathew, Phytochemical composition, antioxidant and anti-nutritional properties of root-bark and leaf methanol extracts of Senna alata L. grown in Nigeria, African Journal of Pure and Applied Chemistry. 9 (2015) 91–97. https://doi.org/10.5897/ajpac2015.0622.

[167] T. Ananthi, K. Subalakshmi, GC-MS analysis of stem bark extracts of Senna alata (L.), Available Online Www.Jocpr.Com Journal of Chemical and Pharmaceutical Research. 8 (2016) 280–283. www.jocpr.com.

[168] E.A. Sisein, R.O. Faith, A.D. Charles, Phenolics and bio compounds detected in Senna alata extract using HPLC and GC-MS analysis respectively, 2022.

[169] P. Rajkumar, S. Selvaraj, D. Velmurugan, GC-MS characterization of the anti-diabetic compounds from the flowers of cassia auriculata (AVARAM): A structure based molecular docking studies Spectral Data Collections View project Computational physics View project, 2016. www.ijirset.com.

[170] V. Prabhu, K. Poonkodi, K. Pradeep, S. Buvaneswari, R. Mini, K. Vimaladevi, M. Anusuya, G. Sibi, Antidandruff activity of cassia auriculata and cassia alata through fatty acids mediated inhibition of malassezia furfur, Journal of Applied and Natural Science. 12 (2020) 532–540. https://doi.org/10.31018/jans.v12i4.2390.

[171] E. Ramasamy, S. Ganesan, Chemical Compounds Investigation of Cassia auriculata Leaves-A Potential Folklore Medicinal Plant, 2015. www.bepls.com.

[172] M.F.H. Ferdosi, A. Javaid, I.H. Khan, S. Ahmad, N. Shad, Analysis of F N-Butanol flower extract of Cassia Fistula through GC-MS and identification of antimicrobial compounds, Pakistan Journal of Phytopathology. 33 (2021) 103–107. https://doi.org/10.33866/PHYTOPATHOL.033.01.0661.

[173] M.J. Kadhim, G.J. Mohammed, I.H. Hameed, In vitro antibacterial, antifungal and phytochemical analysis of methanolic extract of fruit Cassia fistula, Oriental Journal of Chemistry. 32 (2016) 1329–1346. https://doi.org/10.13005/ojc/320307.

[174] J. Sujatha, S. Asokan, MEDFOOD'18 [1 st February 2018] National Conference on Phytochemicals in Medicinal Plants and Food Phytochemical analysis and Antioxidant effect of hexane extract of Cassia fistula using FT-IR and GC-MS analysis, INTERNATIONAL JOURNAL OF PHARMACEUTICS & DRUG ANALYSIS. 6 (2018) 173–179. http://ijpda.com;

[175] M.F.H. Ferdosi, I.H. Khan, A. Javaid, bioactive components of ethyl acetate extract of Cassia fistula flowers, J Anim Plant Sci. 33 (2023). https://doi.org/10.36899/JAPS.2023.3.0643.

[176] P. Sugita, I. Irwanto, I. Bayu, G. Syahbirin, Study of compound from trengguli leaves methanolic extract (Cassia fistula), Asia Pacific Journal of Research. (2014).

[177] A. Ram, C.O. Ola-Davies, Toxicological studies of ethanol leaf extract of Cassia fistula on haematological and biochemical parameters of wistar albino rats, ~ 1407 ~ Journal of Pharmacognosy and Phytochemistry. 8 (2019) 1407–1412.

[178] Z. Peerzada, A.M. Kanhed, K.B. Desai, Effects of active compounds from Cassia fistula on quorum sensing mediated virulence and biofilm formation in Pseudomonas aeruginosa, RSC Adv. 12 (2022) 15196–15214. https://doi.org/10.1039/d1ra08351a.

[179] S. HA, M. AG, A. II, O. AO, M. UA, Phytochemical screening and antibacterial activities of cassia fistula leaf extracts on some selected pathogens, J Pharmacogn Phytochem. 9 (2020) 1779–1783. https://doi.org/10.22271/phyto.2020.v9.i3ad.11574.

[180] C. Mohan, GC-MS analysis of phytochemicals and hypoglycemic effect of Catharanthus roseus in alloxan-induced diabetic rats Regulation of ROS defense system by Hybanthus enneaspermus in CCl4 induce cardiac damage View project study of Pedalium murex phytochemicals in the prevention of kidney stones and urinary tract infections View project, 2015. www.globalresearchonline.net.

[181] M. Kapoor, J. Rani, (Special Issue-1) 2 nd International Conference "Food Security, Nutrition and Sustainable Agriculture-Emerging Technologies" Qualitative and quantitative analysis of phytochemicals by GC-MS and antioxidant activity of Catharanthus roseus (L.) G. Don, ~ 378 ~ Journal of Pharmacognosy and Phytochemistry. (2019) 1.

[182] J. Rani, M. Kapoor, S.B. Dhull, G. Goksen, S. Jurić, Identification and Assessment of Therapeutic Phytoconstituents of Catharanthus roseus through GC-MS Analysis, Separations. 10 (2023). https://doi.org/10.3390/separations10060340.

[183] J. Rani, M. Kapoor, Gas Chromatography-Mass spectroscopic analysis and identification of bioactive constituents of Catharanthus roses and its antioxidant activity, 12 (2019). https://doi.org/10.22159/ajpcr.2019.v12i3.

[184] S. Aziz, K. Saha, N. Sultana, M. Khan, K. Nada, M. Afroze, 2 Comparative studies of Volatile Components of the essential Oil of leaves and flowers of Catharanthus roseus growing in Bangladesh by GC-MS analysis, 2015. www.ijpbr.in.

[185] S. Samiyarsih, N. Fitrianto, E. Proklamasiningsih, Juwarno, J.S. Muljowati, Phytochemical diversity and antimicrobial properties of methanol extract of several cultivars of catharanthus roseus using GC-MS, Biodiversitas. 21 (2020) 1332–1344. https://doi.org/10.13057/biodiv/d210409.

[186] K. Yashoda, K. Deegndra, S. Bimala, Antioxidant, PTP inhibition and A-Amylase inhibition property and GC-MS analysis of methanolic leaves extract of achyranthes aspera and Catrharanthus roseus of NEPAL, Int J Pharm Pharm Sci. (2021) 49–55. https://doi.org/10.22159/ijpps.2021v13i4.40490.

[187] S. Rani, V. Singh, M.K. Sharma, R. Sisodia, GC–MS based metabolite profiling of medicinal plant-Catharanthus roseus under cadmium stress, Plant Physiology Reports. 26 (2021) 491–502. https://doi.org/10.1007/s40502-021-00595-z.

[188] G. Saleem, V. Uttej, N. Reddy, K. Ayyappa, K. Prashanth Babu, N. Manoj, M. Nageri, In-silico evaluation of novel BCL-XL inhibitors and PASS prediction of bioactive compounds from Catharanthus roseus, International Journal of Advances in Engineering and Management (IJAEM). 4 (2022) 122. https://doi.org/10.35629/5252-0405122130.

[189] A. Shil, S. Mukherjee, B. Bishayi, M. Sikdar (Nee) Bhakta, A Comparison of Antibacterial Effects of Catharanthus roseus and Camellia sinensis (Black Tea) and Their Synergistic Effect along with Antibiotic

against Multiple Antibiotic Resistant Strains of Staphylococcus aureus, J Herbs Spices Med Plants. 27 (2021) 135–148. https://doi.org/10.1080/10496475.2020.1815921.

[190] S. P, S.S. Zinjarde, S.Y. Bhargava, A.R. Kumar, Potent α-amylase inhibitory activity of Indian Ayurvedic medicinal plants, BMC Complement Altern Med. 11 (2011). https://doi.org/10.1186/1472-6882-11-5.

[191] I. Nengah, K. Putra, N.S. Antara, N.M. Wartini, Antioxidant Capacity and Bioactives Content of Methanol Extract of Red Flowering Periwinkle (Catharanthus Roseus) Leaf Planted in Different Environmental Conditions, n.d.

[192] I. Jerković, D. Gašo-Sokač, H. Pavlović, Z. Marijanović, M. Gugić, I. Petrović, S. Kovač, Volatile organic compounds from Centaurium erythraea rafn (Croatia) and the antimicrobial potential of its essential oil, Molecules. 17 (2012) 2058–2072. https://doi.org/10.3390/molecules17022058.

[193] N. Ali, M. Nabi, M. Shoaib, I. Shah, G. Ahmed, Shakirullah, Ziauddin, S.W. Ali Shah, M. Ghias, S. Khan, W. Ali, GC/MS analysis, anti-leishmanial and relaxant activity of essential oil of Chenopodium ambrosioides (L.) from Malakand region, Pak J Pharm Sci. 34 (2021) 577–583. https://doi.org/10.36721/PJPS.2021.34.2.REG.577-583.1.

[194] K. Koba, G. Catherine, C. Raynaud, J.P. Chaumont, K. Sanda, N. Laurence, Chemical Composition and Cytotoxic Activity of Chenopodium ambrosioides L.Essential Oil from Togo, Bangladesh J. Sci. Ind. Res. 44(4), 435-440, 2009

[195] I.J. Biosci, G.A. Alitonou, P. Sessou, F.P. Tchobo, J.-P. Noudogbessi, F. Avlessi, B. Yehouenou, C. Menut, P. Villeneuve, D. Codjo, K. Sohounhloue, 58 Alitonou et al. Chemical composition and biological activities of essential oils of Chenopodium ambrosioides L. collected in two areas of Benin International Journal of Biosciences (IJB), 2 (2012) 58–66. http://www.innspub.net.

[196] C.Q. Bai, Z. Long Liu, Q. Zhi Liu, Nematicidal Constituents from the Essential Oil of Chenopodium Ambrosioides Aerial Parts, (n.d.). http://www.e-journals.net.

[197] M.S.G. Chekem, P.K. Lunga, J. de D. Tamokou, J.R. Kuiate, P. Tane, G. Vilarem, M. Cerny, Antifungal properties of Chenopodium ambrosioides essential oil against candida species, Pharmaceuticals. 3 (2010) 2900–2909. https://doi.org/10.3390/ph3092900.

[198] C.M. Jardim, G.N. Jham, O.D. Dhingra, M.M. Freire, Composition and antifungal activity of the essential oil of the brazilian Chenopodium ambrosioides L., J Chem Ecol. 34 (2008) 1213–1218. https://doi.org/10.1007/s10886-008-9526-z.

[199] L. Jirovetz, G. Buchbauer, W. Fleischhacker, V.K. Kaul, Analysis of the Essential Oil of the Leaves of the Medicinal Plant Chenopodium ambrosioides var. anthelminticum (L.) A. Gray from India*, 2000.

[200] M. Mutlu, Z. Bingol, E.M. Uc, E. Köksal, A.C. Goren, S.H. Alwasel, İ. Gulcin, Comprehensive Metabolite Profiling of Cinnamon (Cinnamomum zeylanicum) Leaf Oil Using LC-HR/MS, GC/MS, and GC-FID: Determination of Antiglaucoma, Antioxidant, Anticholinergic, and Antidiabetic Profiles, Life. 13 (2023). https://doi.org/10.3390/life13010136.

[201] Y. Li, D. Kong, X. Lin, Z. Xie, M. Bai, S. Huang, H. Nian, H. Wu, Quality Evaluation for Essential Oil of Cinnamomum verum Leaves at Different Growth Stages Based on GC–MS, FTIR and Microscopy, Food Anal Methods. 9 (2016) 202–212. https://doi.org/10.1007/s12161-015-0187-6.

[202] F. Kalantary, M. Barzegar, Z. Hamidi Esfahani, Control of Aspergillus flavus Growth in Tomato Paste by Cinnamomum zeylanicum and Origanum vulgare L. Essential Oils, 2014. www.jfoodpharmsci.com.

[203] S. Gotmare, E. Tambe, Identification of Chemical Constituents of Cinnamon Bark Oil by GCMS and Comparative Study Garnered from Five Different Countries, 2019.

[204] V.K. Raina, S.K. Srivastava, K.K. Aggarwal, S. Ramesh, S. Kumar, Essential oil composition of Cinnamomum zeylanicum Blume leaves from Little Andaman, India, Flavour Fragr J. 16 (2001) 374–376. https://doi.org/10.1002/ffj.1016.

[205] P.S. Ramalho, V.A.P. De Freitas, A. Macedo, G. Silva, A.M.S. Silva, Volatile components of Cistus ladanifer leaves, Flavour Fragr J. 14 (1999) 300–302. https://doi.org/10.1002/(SICI)1099-1026(199909/10)14:5<300::AID-FFJ830>3.0.CO;2-X.

[206] Z. H, E. M, A. F, T. A, W. J, S. M, E. A, Chemical composition and antioxidant activity of essential oil, various organic extracts of Cistus ladanifer and Cistus libanotis growing in Eastern Morocco, Afr J Biotechnol. 12 (2013) 5314–5320. https://doi.org/10.5897/ajb2013.12868.

[207] H. Greche, N. Mrabet, S. Zrira, M. Ismaïli-Alaoui, B. Benjilali, A. Boukir, The Volatiles of the leaf oil of cistus ladanifer l. Var. Albiflorus and labdanum extracts of moroccan origin and their antimicrobial Activities, Journal of Essential Oil Research. 21 (2009) 166–173. https://doi.org/10.1080/10412905.2009.9700140.

[208] K. Bouothmany , M. Bourhia , N. Aoussar, Leaf Extracts of Cistus ladanifer Exhibit Potent Antioxidant and Antiproliferative Activities against Liver, Prostate and Breast Cancer Cells, Appl. Sci. 2022, 12(17), 8603; https://doi.org/10.3390/app12178603.

[209] H. Qi, S. Ding, Z. Pan, X. Li, F. Fu, Characteristic volatile fingerprints and odor activity values in different citrus-tea by HS-GC-IMS and HS-SPME-GC-MS, Molecules. 25 (2020). https://doi.org/10.3390/molecules25246027.

[210] Z.W. Yoo, N.S. Kim, D.S. Lee, Comparative Analyses of the Flavors from Hallabong (Citrus sphaerocarpa) with Lemon, Orange and Grapefruit by SPTE and HS-SPME Combined with GC-MS, Bull Korean Chem Soc. 25 (2004) 271–279. https://doi.org/10.5012/bkcs.2004.25.2.271.

[211] A.A.R. Alves, M.J.C. Rezende, A.M.C. Hovell, H.R. Bizzo, A. Carolina, L. Oliveira, S. V Rodrigues, C.M. Rezende, Comparison between GC-MS-SIM and GC-ECD for the Determination of Residues of Organochlorine and Organophosphorus Pesticides in Brazilian Citrus Essential Oils, 2012.

[212] K. Tomer, N.K. Sethiya, A. Shete, V. Singh, Isolation and characterization of total volatile components from leaves of citrus limon, www.japtr.org.

[213] N.Q. Fadilah, A. Jittmittraphap, P. Leaungwutiwong, P. Pripdeevech, D. Dhanushka, C. Mahidol, S. Ruchirawat, P. Kittakoop, Virucidal Activity of Essential Oils From Citrus x aurantium L. Against Influenza A Virus H1N1: Limonene as a Potential Household Disinfectant Against Virus, Nat Prod Commun. 17 (2022). https://doi.org/10.1177/1934578X211072713.
